# Visible‐Light Switching of Metallosupramolecular Assemblies**

**DOI:** 10.1002/chem.202104461

**Published:** 2022-02-19

**Authors:** Aaron D. W. Kennedy, Ray G. DiNardi, Lucy L. Fillbrook, William A. Donald, Jonathon E. Beves

**Affiliations:** ^1^ School of Chemistry The University of New South Wales Sydney NSW 2052 Australia

**Keywords:** azobenzenes, molecular cages, photoswitches, self-assembly, supramolecular chemistry

## Abstract

A photoswitchable ligand and palladium(II) ions form a dynamic mixture of self‐assembled metallosupramolecular structures. The photoswitching ligand is an *ortho*‐fluoroazobenzene with appended pyridyl groups. Combining the *E*‐isomer with palladium(II) salts affords a double‐walled triangle with composition [Pd_3_L_6_]^6+^ and a distorted tetrahedron [Pd_4_L_8_]^8+^ (1 : 2 ratio at 298 K). Irradiation with 410 nm light generates a photostationary state with approximately 80 % of the *E‐*isomer of the ligand and results in the selective disassembly of the tetrahedron, the more thermodynamically stable structure, and the formation of the triangle, the more kinetically inert product. The triangle is then slowly transformed back into the tetrahedron over 2 days at 333 K. The *Z*‐isomer of the ligand does not form any well‐defined structures and has a thermal half‐life of 25 days at 298 K. This approach shows how a thermodynamically preferred self‐assembled structure can be reversibly pumped to a kinetic trap by small perturbations of the isomer distribution using non‐destructive visible light.

## Graphical abstract

**Visible‐light photoswitching**: A photoswitchable ligand and palladium(II) ions form a dynamic mixture of self‐assembled metallosupramolecular structures. Visible‐light photoswitching of the azobenzene‐based ligand reversibly pumps the system away from equilibrium towards the thermodynamically unstable structure.

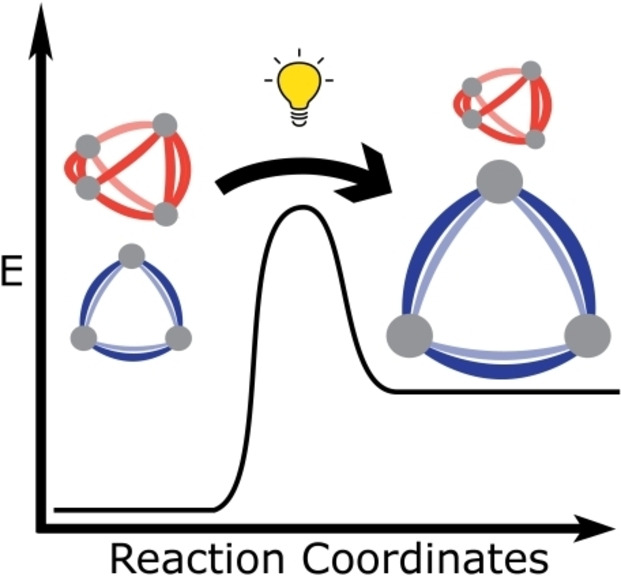

## Introduction

**Figure 1:**
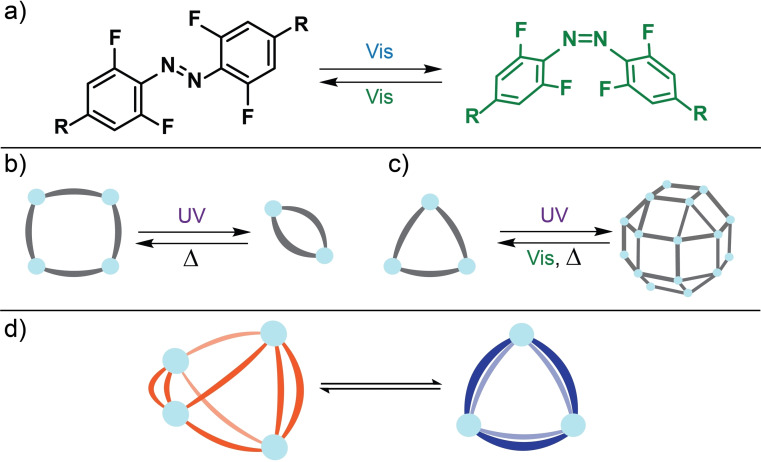
a) *ortho*‐Fluoroazobenzene switching induced by visible light. We will define the parent compound has *R*=H. Previous examples of light‐induced structural changes in metal‐based supramolecular structures include b) square‐triangle^[30]^ and c) triangle‐sphere transformations.^[27b]^ d) This work, showing light induced structural changes with both structures comprising the same ligand isomerization state.

The structure and function of self‐assembled species, such as molecular cages, can be controlled using stimuli‐responsive components. Different stimuli have been used to perturb metal‐template supramolecular assemblies^[1]^ including light,^[2]^ guest molecules,^[3]^ pH changes,^[4]^ competing ligands,^[5]^ and changes to solvent.^[6]^ Light, especially the visible spectrum,^[7]^ is appealing as it is easy to apply with high spatial and temporal resolution, and has the potential for highly specific targeting.^[8]^ Molecular photoswitches,^[9]^ can be isomerized reversibly by light, with each isomer having different geometries and electronic properties. These differences in properties have been used to control the properties of gels,^[10]^ polymer assemblies,^[11]^ or liquid crystals,^[12]^ and to perform functions including acting as light‐activated receptors^[13]^ or pharmaceuticals,^[14]^ or pumping systems away from thermodynamic equilibrium.^[15]^ The most studied photoswitches are those based on azobenzene,^[16]^ which can be isomerized between a stable *E*‐isomer and a metastable *Z*‐isomer. Azobenzene‐type molecules that operate effectively with visible light have been recently developed,[^9c^, ^17^] with one of the most successful modifications being the introduction of *ortho*‐fluoro substituents (Figure 1a).^[18]^ These *ortho*‐fluoroazobenzenes allow bidirectional visible‐light switching with thermal half‐lives that can exceed 2 years. They have been incorporated into MOFs^[18i]^ and discrete self‐assembled structures,^[18h]^ and have been used to control molecular folding^[18e]^ or the function of enzymes.^[18j]^

Conceptually, there are two approaches for combining photoswitches with discrete self‐assembled structures: encapsulation, or direct incorporation as part of the structure. The first strategy involves binding the photoactive unit inside a cavity, such as encapsulating azobenzene‐type compounds.^[19]^ Encapsulating a photoswitch can also restrict switching or perturb the balance of isomers.[^19b^, ^20^] Although there are many examples of large photoswitchable assemblies,^[21]^ such as micelles, vesicles, or liquid crystals^[12]^ formed with polymers,^[11]^ there are relatively few examples of photoswitches being self‐assembled into well‐defined, discrete structures. For example, pyridyl‐based ligands and palladium(II) were self‐assembled into a [Pd_12_L_24_]^24+^ molecular sphere with endohedral azobenzene groups^[2a]^ which could be switched with UV to increase the hydrophilicity of the sphere's cavity. Other examples of pyridyl‐functionalized photoswitches include [M_2_L_4_]^4+^ cages formed with stiff‐stilbenes and palladium(II),^[22]^ chiral [M_6_L_3_]^6+^ metallocycles formed from dithienylethene (DTEs)^[23]^ and platinum(II),^[24]^ and related ligands reacted with iron(II/III) to form [Fe_2_L_3_]^n+^ helicates.^[25]^

The first example of a molecular cage with functioning azobenzene‐type photoswitches as linkers used cyclotriguaiacylene units with three appended pyridyl‐azo‐phenyl photoswitches and iridium(III) complexes to form [Ir_3_L_2_]^3+^ cages.^[26]^ The flexible linkers allowed photoswitching to occur without disrupting the cage connectivity. Another example used a 3‐pyridyl functionalized azobenzene and palladium(II) to form M_2_L_4_ dimers that disassembled on irradiation.^[27]^ However, the most well‐studied photoswitchable cages are based on pyridyl‐functionalized DTE photoswitches assembled with palladium(II) ions reported by the Clever group.^[28]^ The difference in geometries has been exploited for selective guest uptake,[^28a^, ^28d^, ^28e^] and control over macromolecular properties when incorporated into gels.^[29]^

Photoswitching units can also modulate the connectivity of metallosupramolecular structures; however, this usually leads to the assembly of new non‐discrete structures.[^27^, ^30^] There are few reported examples of modulation between discrete metallosupramolecular structures, with some key examples represented in Figure 1b,c.[^28b^, ^31^] One example used azobenzene or stilbene based ligands to form [M_2_M′_2_L_4_]^8+^ (M=Pd, M′=Pd or Re) macrocycles where UV irradiation formed the smaller [M_2_L_2_]^4+^ species.[^31^, ^32^] Other examples use DTE‐based ligands.[^28b^, ^28e^] For one system, the open and closed isomers give rise to a double‐walled triangle (as the major component) and a cuboctahedral sphere, respectively.^[28b]^ A more recent example was able to eject one ligand from a Pd_2_L_4_ cage upon irradiation.^[28e]^ Despite these examples, there are no reports of metallosupramolecular structures which can be reversibly rearranged using visible light only.

Herein, we report a system of two discrete metallosupramolecular assemblies, formed from an *ortho*‐fluoroazobenzene ligand (Figure 1d). The system can be driven out‐of‐equilibrium with visible light due to the different kinetic labilities of the structures. To the best of our knowledge this is one of the only examples of light‐induced connectivity changes and the first example of all‐visible light switching between discrete structures.

## Results and Discussion

**Scheme 1:**
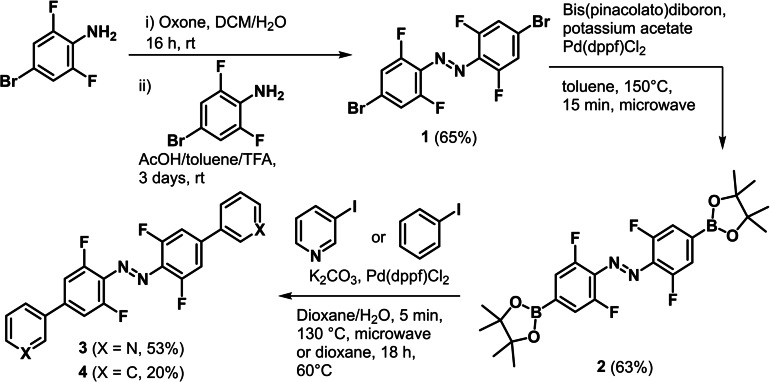
Synthesis of pyridyl‐appended *ortho*‐fluoroazobenzene **3**. a) i) Oxone, DCM/H_2_O (1 : 4), 16 h, RT. ii) 4‐Bromo‐2,6‐difluoroaniline, AcOH/toluene/TFA (6 : 6 : 1), 72 h, RT. b) Pd(dppf)Cl_2_, (BPin)_2_, KOAc, toluene, 150 °C (μW), 15 min. c) 3‐Iodopyridine, Pd(dppf)Cl_2_, K_2_CO_3_, 1,4‐dioxane/H_2_O (7 : 1), 130 °C (μW), 5 min; or iodobenzene, Pd(dppf)Cl_2_, K_2_CO_3_, 1,4‐dioxane, 60 °C (μW), 18 h. See the Supporting Information section 2 for details.

**Figure 2:**
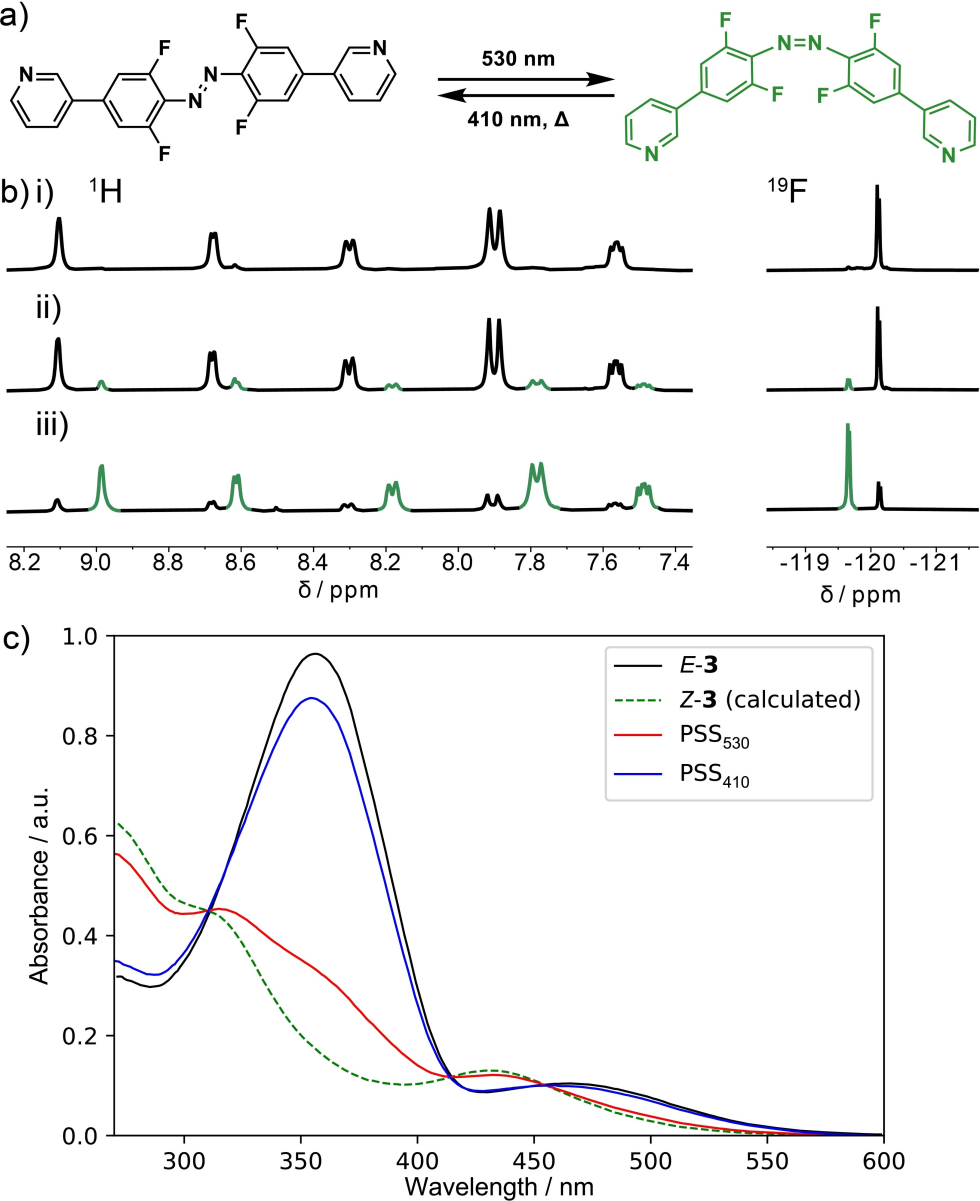
Photoswitching between *E*‐**3** and *Z*‐**3** monitored by b) ^1^H and ^19^F NMR spectroscopy (DMSO‐*d_6_*, 298 K) of photoswitch **3** after i) thermal equilibration, ii) irradiation at 410 nm (PSS_410_ contains 15 % *Z*‐**3**), iii) irradiation at 530 nm (PSS_530_ contains 80 % *Z*‐**3**). Isomer ratios were calculated from ^19^F NMR signal integrations. c) UV‐Vis absorption spectra at 298 K in DMSO. The spectrum of *Z‐* **3** was calculated using the isomer ratio determined by ^19^F NMR signal integrations of a sample irradiated at 530 nm.

We synthesized substituted *ortho*‐fluoroazobenzenes in moderate yield over three steps from commercially available 4‐bromo‐2,6‐difluoroaniline (see Supporting Information sections 1 and 2 for details).^[33]^ Compound **1** was obtained in 65 % yield using a methodology previously used to generate unsymmetrical azobenzene derivatives.[^18a^, ^18b^, ^34^] Boronic ester substituted *ortho*‐fluoroazobenzene **2** has been previously reported,^[18d]^ but use of microwave heating allowed us to reduce the reaction time to 15 minutes with a trivial work‐up that excluded chromatography. Suzuki coupling gave the photoswitchable ligand **3** (53 % yield) and the control compound, phenyl derivative **4** (20 % yield; Scheme 1). The second coupling reaction did not always reach completion despite the arylhalide being in excess, with the mono‐substituted product being identified and characterized (see Supporting Information sections 2.4 and 16 for details). This suggests the second coupling reaction is considerably more difficult than the first. The compounds were isolated as mixtures of the thermodynamically favored *E*‐isomer and the metastable *Z*‐isomer. Heating a solution of **3** in DMSO‐*d_6_* generated the pure *E*‐**3** isomer as observed by ^1^H and ^19^F NMR spectra (Figure 2b).^[35]^

The UV‐vis absorption of photoswitchable ligand *E*‐**3** (Figure 2c) extends into the visible, with a visible absorption maximum at 466 nm assigned as the n‐π* band and a band at 356 nm assigned to the π‐π* transition (in DMSO at 298 K). Both transitions are red‐shifted relative to the parent *ortho*‐fluoroazobenzene, which has an n‐π* transition at 460 nm and a π‐π* transition at 314 nm (in DMSO at 298 K).^[18a]^ The larger red‐shift of the n‐π* band compared to the π‐π* was also reported for 2,2′,6,6′‐tetrafluoro‐4,4′‐diacetamidoazobenzene,^[18a]^ suggesting this effect is due to substitution with electron donating groups.

Photoswitchable ligand **3** undergoes reversible photoswitching with visible light (Figure 2c). Irradiation of **3** with an LED centered at 530 nm generated a photostationary state comprising 80 % *Z*‐**3** (calculated from ^19^F NMR signal integrations, Figure 2b and Supporting Information section 4.1). Subsequent irradiation at 410 nm generated a new photostationary state comprising 85 % *E*‐**3**. The calculated absorption spectrum^[36]^ of *Z*‐**3** shows an n‐π* transition with an absorption maximum at 432 nm, slightly red‐shifted compared to unsubstituted or ester substituted *ortho*‐fluoroazobenzenes (λ_max_=417–421 nm).^[18a]^ The separation between the n‐π* bands for the two isomers of **3** (Δλ_n‐π*_=33 nm) is similar to that found for other *ortho*‐fluoroazobenzenes with electron‐donating groups in the *para* position,^[18a]^ but less than that for the parent *ortho*‐fluoroazobenzenes or examples with electron withdrawing groups (Δλ_n‐π*_=30 to 50 nm).^[18b]^ This poorer band separation reduces the selectivity of photoswitching. Photoswitchable ligand *Z*‐**3** has a thermal half‐life of ≈25 days at 298 K (thermal barrier of 110 kJmol^−1^, measured at 333 K in DMSO, see Supporting Information section 4.3). Photoswitch **4** has similar properties to photoswitchable ligand **3**. For example, photoswitch **4** has an n‐π* absorption band at 462 nm and a π‐π* band at 360 nm, and *Z*‐**4** has a thermal half‐life of ≈37 days at 298 K (see Supporting Information section 5 for details). Photoswitches **3** and **4** both have shorter half‐lives compared to the parent *ortho*‐fluoroazobenzene which has a half‐life of 700 days (thermal barrier of 117 kJmol^−1^, measured at 333–373 K in DMSO).^[18b]^

**Figure 3:**
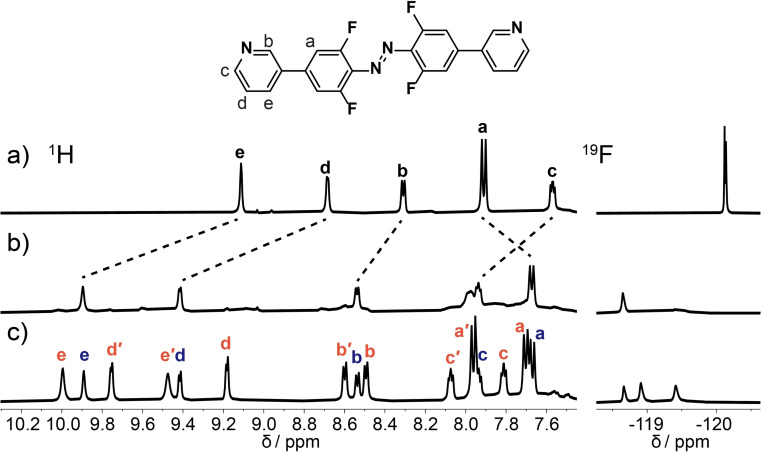
Formation of a mixture of [Pd_3_(**3**)_6_]^6+^ (blue) and [Pd_4_(**3**)_8_]^8+^ (red) in DMSO‐*d_6_* monitored by ^1^H and ^19^F NMR spectroscopy. ^1^H and ^19^F NMR spectra of *E*‐**3** in DMSO‐*d_6_* ([**3**]=19 mM) a) before addition of palladium, b) immediately after addition of [Pd(CH_3_CN)_4_](BF_4_)_2_ and c) after equilibration in the dark for 10 days. The signals for the [Pd_4_(**3**)_8_]^8+^ species denoted by a dash (′) correspond to the single‐bridged ligands. See Supporting Information section 10 for assignment for [Pd_3_(**3**)_6_]^6+^, Supporting Information section 11 for assignment for [Pd_4_(**3**)_8_]^8+^.

Having characterized photoswitchable ligand **3**, we next investigated its self‐assembly with palladium(II) ions. When [Pd(CH_3_CN)_4_](BF_4_)_2_ was added to *E*‐**3** in DMSO‐*d_6_* a new species was immediately formed as observed by ^1^H and ^19^F NMR spectroscopy (Figure 3b, Supporting Information section 6). Equilibration in the dark at room temperature over 10 days led to the formation of a new, lower symmetry, assembly comprising 69 % of the mixture (Figure 3c). This process also occurs within 10 min at 60 degrees, see Supporting Information section 6.

**Figure 4:**
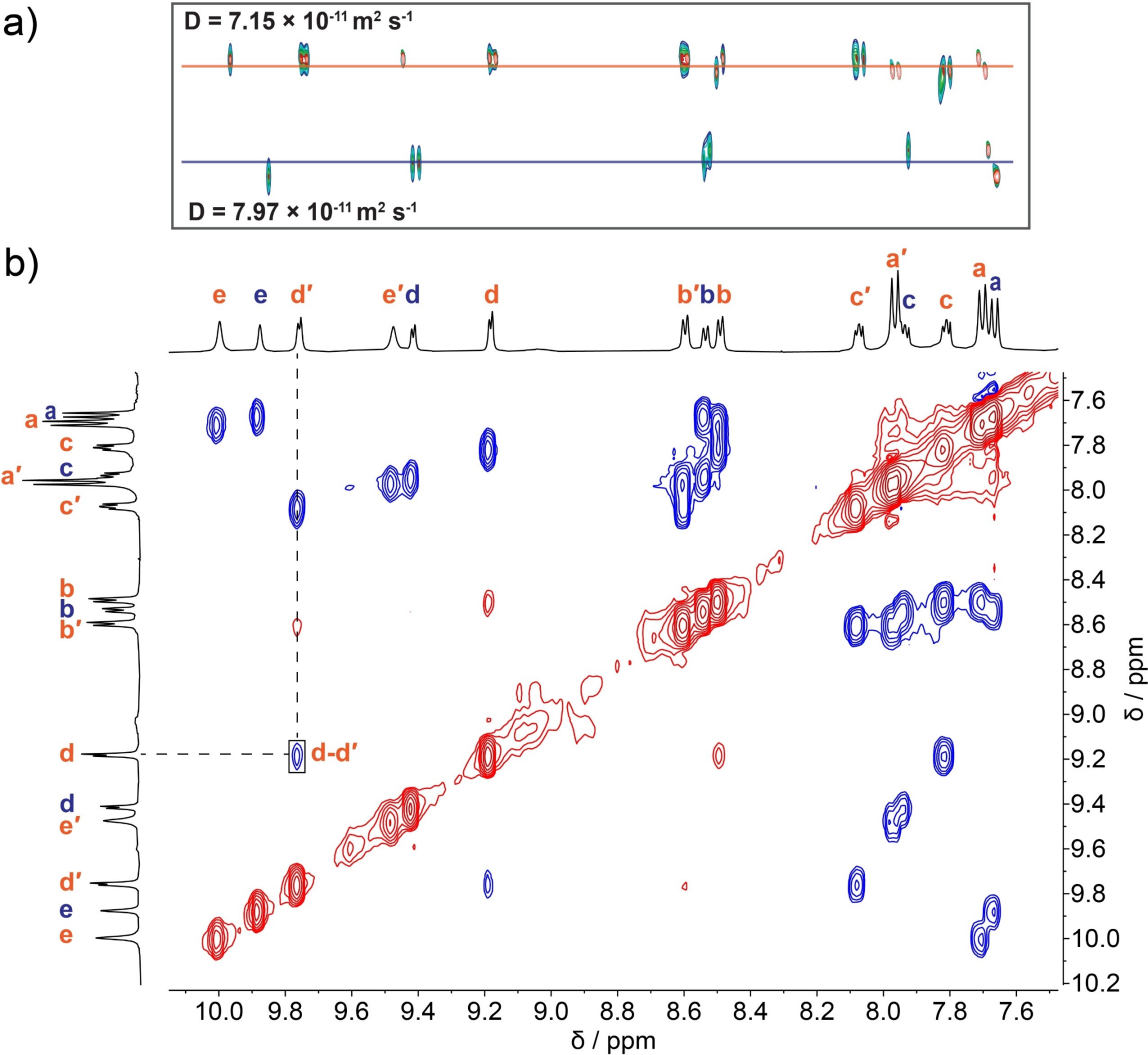
Identification of the ^1^H NMR signals for an equilibrated mixture of self‐assembled species, [Pd_3_(**3**)_6_]^6+^ and [Pd_4_(**3**)_8_]^8+^, in the dark. a) ^1^H DOSY spectrum (500 MHz, DMSO‐d_6_, 298 K) of a mixture of [Pd_3_(**3**)_6_]^6+^ and [Pd_4_(**3**)_8_]^8+^. Diffusion constants calculated based on fitting of the peak integrations (see Supporting Information section 8.2). b) ^1^H ROESY spectrum spectrum (600 MHz, DMSO‐*d*_6_, 298 K) with the through‐space interaction between peaks belonging to different ring systems highlighted.

Using ^1^H NMR diffusion (Figure 4a) and ROESY NMR (Figure 4b) data, we identified two separate species with the higher symmetry species having a smaller hydrodynamic diameter (27 vs. 31 Å). A similar reaction of ligand **3** with a more soluble palladium salt^[37]^ gave the same two structures albeit with a slightly different relative abundance, see Supporting Information section 7.

Characteristic downfield shifts of the ^1^H NMR signals for pyridyl protons indicate coordination to the metal ion (see Supporting Information section 8.1 for full details).^[38]^ The initially formed species has the same number of ^1^H NMR signals as the free ligand. The ^1^H NMR signal for H^a^ (see Figure 3 for atom labels) is shifted upfield by ≈0.2 ppm compared to the free ligand, consistent with shielding effects commonly seen for related structures.[^38b^, ^39^] The second species formed has lower symmetry with a doubling of all ligand signals (Figure 3c). In this case the signal of the H^e′^ proton is shifted significantly upfield (≈0.4 ppm) compared to the symmetric species. The ^19^F NMR spectrum confirms the reduced symmetry with two peaks observed for the lower symmetry species. The significant peak shifts observed in the NMR spectra did not allow unambiguous assignment of the *E*/*Z*‐isomerization state.

The UV‐vis absorption spectrum of the mixture was also unhelpful for assigning the *E* or *Z* isomer composition (see Supporting Information section 15.1). Therefore, a degradation experiment was performed. 4‐Dimethylaminopyridine (DMAP) was added to the equilibrated mixture in the dark which rapidly disassembled the structures to form exclusively *E*‐**3** and [Pd(DMAP)_4_](BF_4_)_2_ as seen by ^1^H NMR spectroscopy (See Supporting Information section 12). This degradation experiment indicates that the changes in the ^1^H NMR spectrum and UV‐visible absorption spectrum are due to the constrained local environment or distortions of the *E*‐**3** ligand imposed by the structure, rather than isomerization of the ligand.

**Figure 5:**
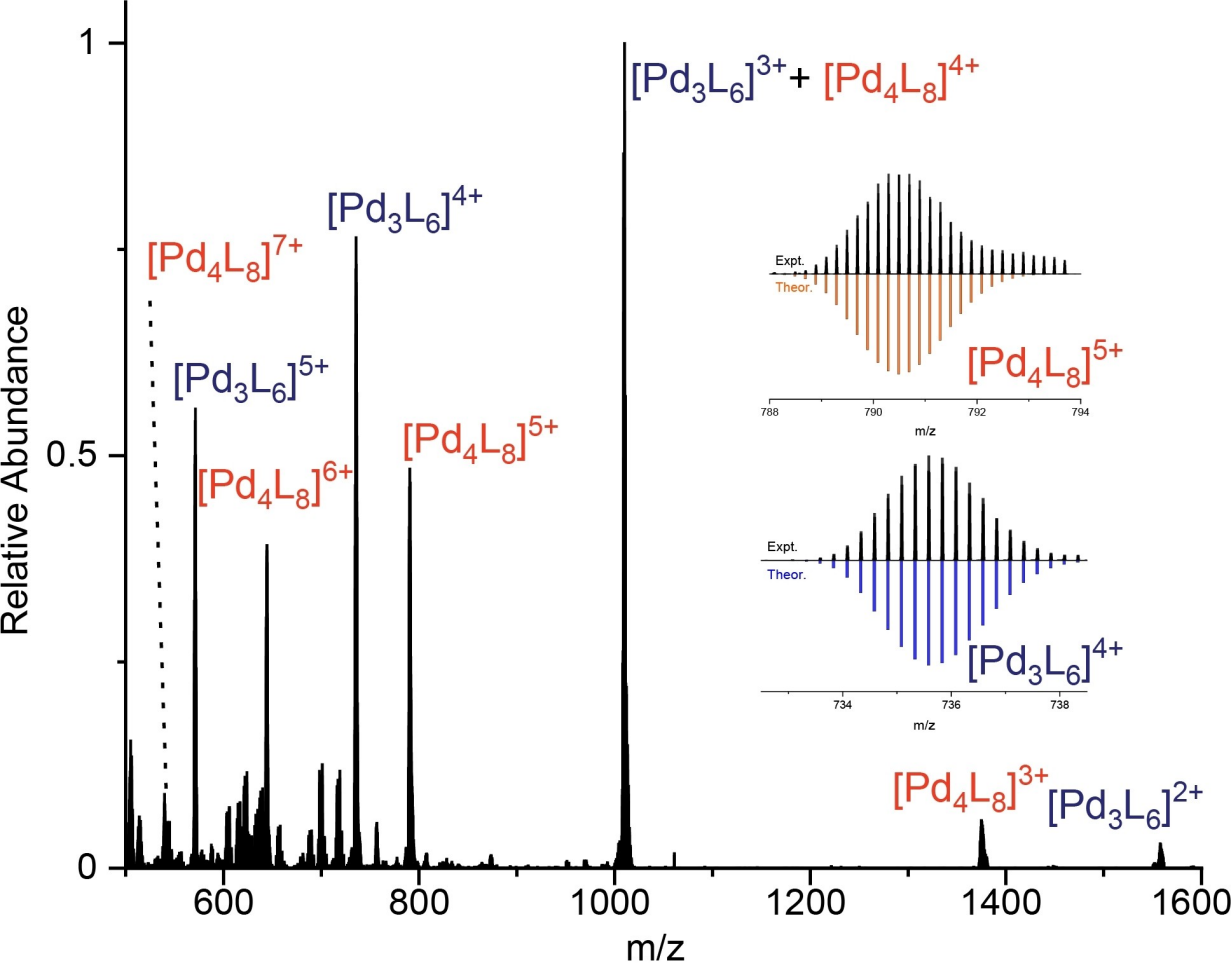
High‐resolution ESI‐MS of a mixture of [Pd_3_(**3**)_6_]^6+^ and [Pd_4_(**3**)_8_]^8+^. The inset shows two peaks for the self‐assembled species and their calculated isotope distribution. For other details, see the Supporting Information section 9.

**Figure 6:**
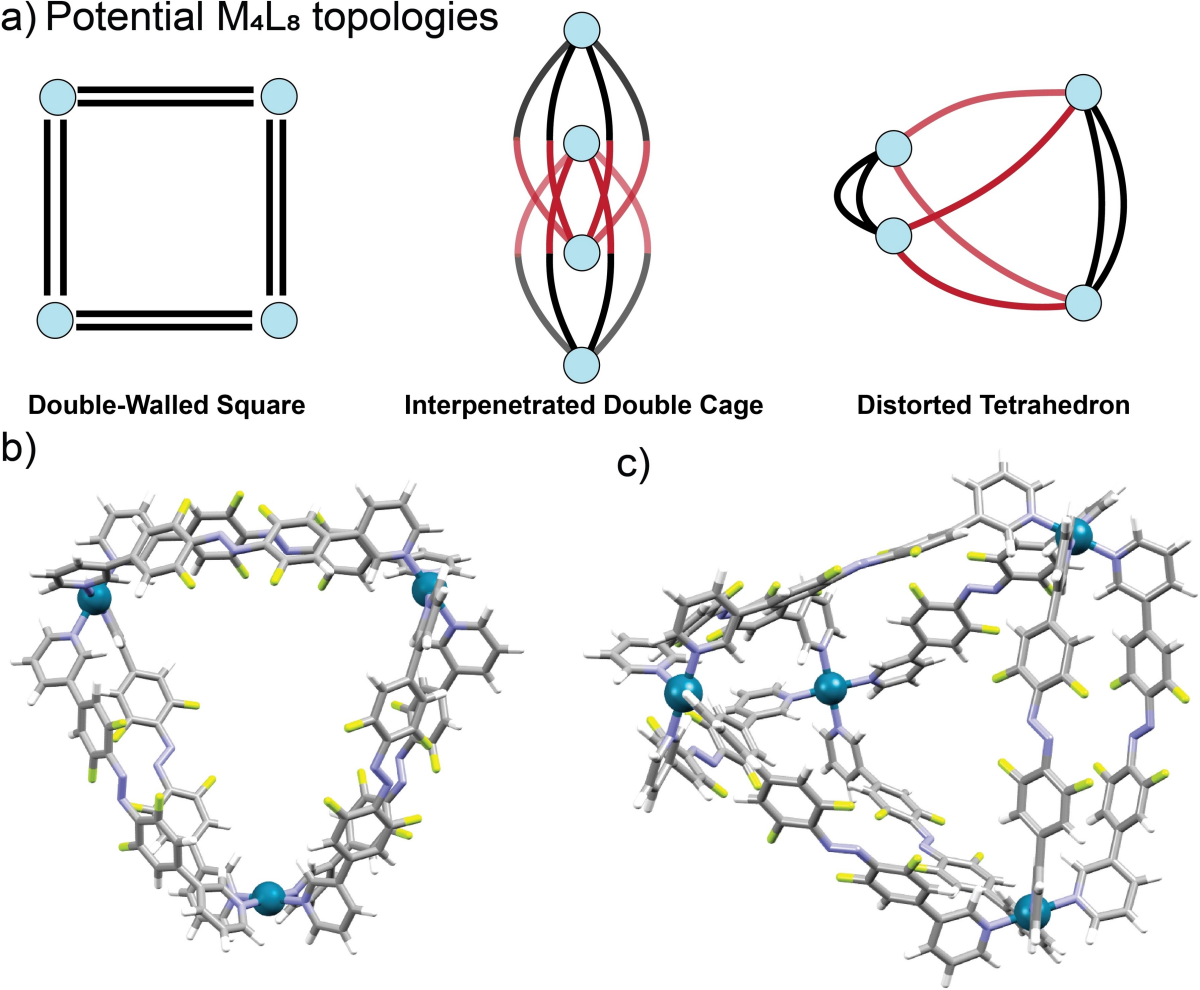
a) Possible topologies for the [Pd_4_(**3**)_8_]^8+^ composition. Blue spheres represent Pd^II^ ions and black and red edges represent non‐equivalent ligands. b) Proposed structure for the [Pd_3_(**3**)_6_]^6+^ assembly and c) Proposed structure for the [Pd_4_(**3**)_8_]^8+^ assembly.

High resolution electrospray ionization mass spectrometry (ESI‐MS) identified two major species, a [Pd_3_(**3**)_6_]^6+^ and a [Pd_4_(**3**)_8_]^8+^ assembly (Figure 5, Supporting Information section 9) with a range of charge states corresponding to sequential loss of BF_4_^−^ anions from these structures. The combination of NMR and MS data, together with preliminary molecular modelling, was used to propose structures for [Pd_3_(**3**)_6_]^6+^ and [Pd_4_(**3**)_8_]^8+^ (Figure 6b and 6c). For the [Pd_3_(**3**)_6_]^6+^ species, the NMR spectra indicates a highly symmetrical structure, which is assigned as a double‐walled triangle.^[40]^

Crude molecular modelling suggests the ligands are in close contact, consistent with the downfield shift of the pyridyl signals for the ligand in the ^1^H NMR spectrum. The modelled structure has a longest axis of 25 Å, which is in good agreement with the hydrodynamic diameter calculated from the NMR diffusion data (27 Å), especially given the significant anisotropy of the structure.

For the species with composition [Pd_4_(**3**)_8_]^8+^, several possibilities can be considered (Figure 6a): a double‐walled square,[^40b^, ^40f^] an interpenetrated double cage^[41]^ or a distorted tetrahedron.[^40a^, ^40h^, ^42^] The double‐walled square would nominally have *D*_4h_ symmetry with all pyridyl rings being equivalent. This is inconsistent with the observed number of signals in the NMR spectra. The interpenetrated double cage structure would show a doubling of the ^1^H and ^19^F NMR signals as observed. However, in previous reports of such topologies the transient formation of a [Pd_2_L_4_]^4+^ cage was observed in the ^1^H NMR spectrum and by ESI‐MS.^[41b]^ Such species were not observed for the current system and molecular modelling also suggests significant strain would be required in the [Pd_2_(**3**)_4_]^4+^ subunit. The structure is therefore proposed as a distorted tetrahedron with C_2v_ symmetry. Ligands with 3‐pyridyl groups bridged by phenyl[^40a^, ^42b^] or BINOL linkers^[42a]^ have been previously assembled into analogous distorted tetrahedra with palladium(II), but the structure remains rare.[^40a^, ^40h^, ^42a^, ^42b^, ^42d^] For [Pd_4_(**3**)_8_]^8+^ the groups of signals from the non‐equivalent ligands were assigned using 2D NMR techniques and by comparing to previously reported examples.^[40a]^ The local environment for the double‐bridged ligands is similar to that observed for the double‐walled triangle [Pd_3_(**3**)_6_]^6+^ species.

The ^1^H NMR spectrum also suggests the conformation of the single‐bridged ligands are similar to free *E*‐**3**. The molecular model suggests a longest axis (28 Å) in agreement with the calculated hydrodynamic diameter (31 Å) from the diffusion NMR data.

Variable temperature ^1^H NMR spectra (Supporting Information section 8.3) confirmed the two species were in a temperature‐sensitive equilibrium. Increasing the temperature to 353 K gave a mixture containing 63 % of the smaller [Pd_3_(**3**)_3_]^6+^ species. This is ascribed to entropic considerations, as proposed in other systems.^[43]^ The system initially remained out of equilibrium upon cooling to 298 K, reaching the original distribution after 18 h in the dark. This indicates that the double‐walled triangle acts as a kinetic trap for the system, consistent with the initial observations upon combination of *E*‐**3** and [Pd(CH_3_CN)_4_](BF_4_)_2_. The distribution change observed during these variable temperature NMR experiments was also consistent with that obtained when the self‐assembly was performed at 60 degrees (see Supporting Information section 6).

Having investigated the self‐assembly properties of *E*‐**3**, we next investigated the behavior of the *Z*‐**3** isomer. A sample of **3** was enriched to 80 % *Z*‐**3** by irradiation with 530 nm light, then combined with [Pd(CH_3_CN)_4_](BF_4_)_2_ in DMSO‐*d*_6_. The resulting poorly resolved ^1^H NMR spectrum suggests the formation of non‐distinct or polymeric products, which do not significantly resolve over time (see Supporting Information section 13). To understand the self‐assembly behavior, we next considered the binding affinity of the ligand for palladium(II) centers. To the best of our knowledge, and despite their widespread use in supramolecular self‐assembly, quantitative binding constants for simple pyridine derivatives to palladium(II) ions do not appear to be reported. However, the binding of 4‐dimethylaminopyridine (DMAP) to a palladium(II) diphosphine complex has been reported with a binding constant of *K*_a_=1.7×10^6^ M^−1^ in DMSO, with other simple pyridine derivatives being too weakly bound for their binding constants to be reported in that study.^[44]^ To study a single 1 : 1 binding event, we used a palladium(II) complex with a tridentate terpyridine ligand (ttpy=4′‐(*para*‐tolyl)‐2,2′ : 6′,2′′‐terpyridine), [Pd(ttpy)(DMSO)](BF_4_)_2_, which has a weakly bound solvent molecule that can be readily exchanged for the other ligands. We used 3‐methylpyridine as a simple monodentate ligand (see Supporting Information section 3 for synthetic details). Isothermal titration calorimetry (ITC) was used to measure the 1 : 1 binding constant (see Supporting Information section 14.1). The relative binding constant is 1.73×10^4^ mol^−1^ in DMSO, equivalent to a binding energy of just 24 kJ ⋅ mol^−1^ at 298 K. Variable temperature NMR studies were also consistent with the rate of ligand exchange being intermediate on the NMR timescale, with only broad signals observed for the ligand (see Supporting Information section 4.4). Similar ITC measurements with **3** and [Pd(ttpy)(DMSO)](BF_4_)_2_ indicated only weaker binding (*K*_a_≈1000), although solubility difficulties prevented quantitative measurements. Competitive binding experiments monitored by ^1^H NMR spectroscopy confirmed that **3** is nearly completely displaced from [Pd(ttpy)(DMSO)](BF_4_)_2_ when one equivalent of 3‐methylpyridine is added (see Supporting Information section 14.2), consistent with **3** being a surprisingly poor ligand for palladium(II). We also investigated the influence of palladium(II) ions on the photoswitching behavior of ligand **3**. After irradiating with 530 nm light, the thermal *Z*→*E* half‐life of ligand **3** in the dark was measured by UV‐vis absorption, (see Supporting Information section 4.3 and 4.4). No difference in thermal half‐life was found when 100 equivalents of [Pd(ttpy)(DMSO)](BF_4_)_2_ was added. A similar control experiment with photoswitch **4** also showed no change in switching properties when combined with palladium(II), see Supporting Information section 5.4. As ligand **3** has only weak affinity for palladium(II), its ability to assemble into discrete structures suggests that cooperativity is responsible for stabilizing the resulting self‐assembled structures.

**Figure 7:**
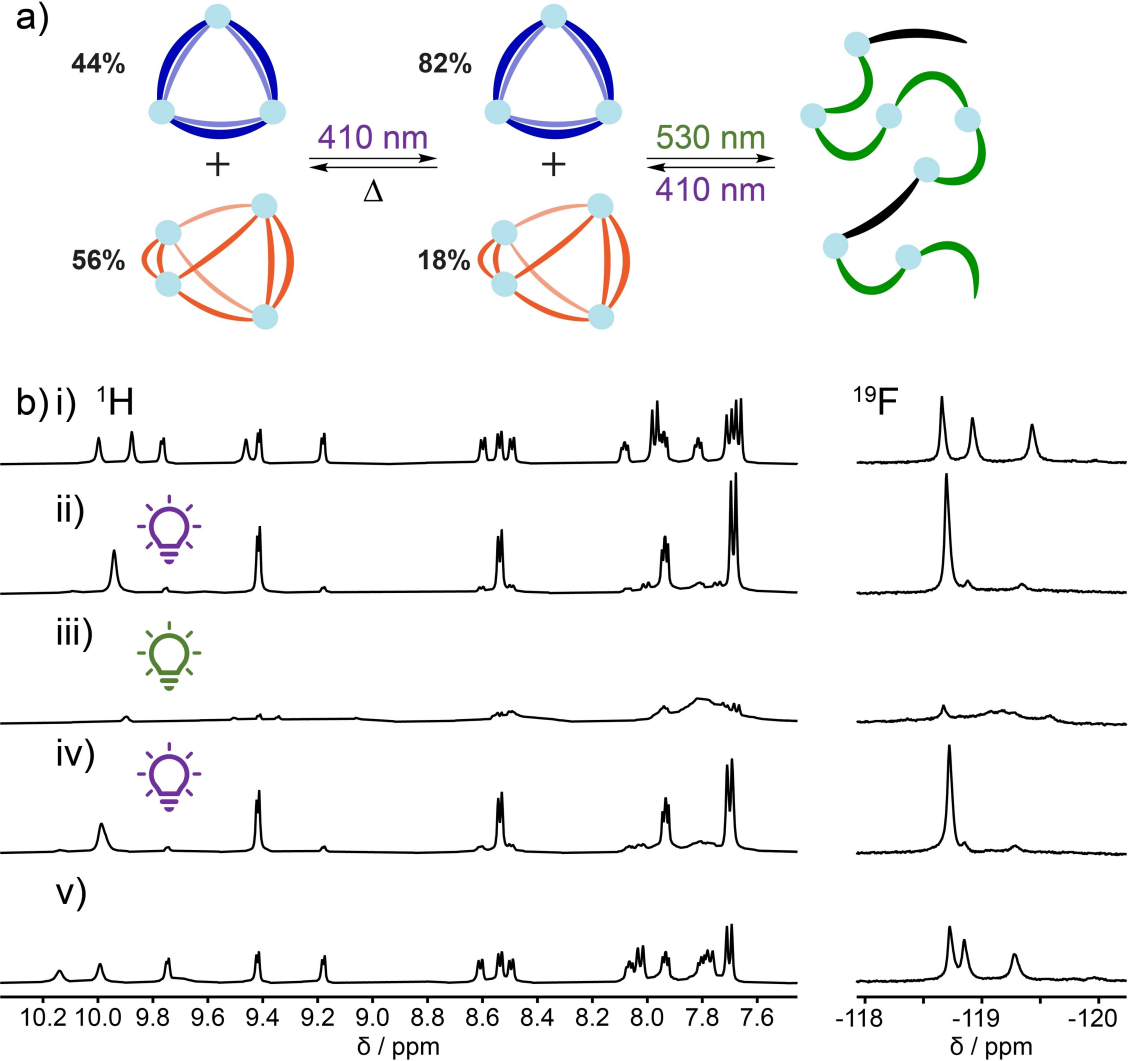
Response to light irradiation of a sample of [Pd_3_(**3**)_6_]^6+^ and [Pd_4_(**3**)_8_]^8+^ in DMSO‐*d*_6_ ([**3**]=19 mM). a) Cartoon representation of the structural changes induced by irradiation. b) ^1^H NMR and ^19^F spectra of i) a mixture of [Pd_3_(**3**)_6_]^6+^ and [Pd_4_(**3**)_8_]^8+^ in DMSO‐*d_6_* (44 % [Pd_3_(**3**)_6_]^6+^); ii) the same sample after irradiation with 410 nm light for 10 minutes (82 % [Pd_3_(**3**)_6_]^6+^); iii) the same sample after irradiation with 530 nm light for 10 minutes; iv) the same sample after irradiation with 410 nm light for 10 minutes again; and v) the same sample after 2 days of being heated at 60 °C followed by 6 h of equilibrating at room temperature.

The distribution between [Pd_3_(**3**)_6_]^6+^ and [Pd_4_(**3**)_8_]^8+^ can be pumped away from equilibrium using light, even though *Z*‐**3** did not self‐assemble into well‐defined structures with palladium(II) ions. After irradiating a mixture of [Pd_3_(**3**)_6_]^6+^ and [Pd_4_(**3**)_8_]^8+^ in DMSO‐*d_6_* with 410 nm light for 10 minutes, ^1^H and ^19^F NMR spectroscopy reveals a significant increase in the population of [Pd_3_(**3**)_6_]^6+^, while also showing the concomitant decrease of [Pd_4_(**3**)_8_]^8+^ (Figure 7b, ii). No new signals were observed, suggesting no new well‐defined self‐assembled species were formed. This observation was reaffirmed by high‐resolution ESI‐MS, where the relative population of [Pd_3_(**3**)_6_]^6+^ increased after irradiation with 410 nm light (see Supporting Information section 15). Irradiating the sample with 530 nm light for 10 minutes resulted in the disassembly of the two species as seen in the poorly defined ^1^H and ^19^F NMR spectra, suggesting the formation of polymeric species, or other low symmetry species (Figure 7b, iii). The large population of [Pd_3_(**3**)_6_]^6+^ could be recovered by irradiating the system again with 410 nm light for 10 minutes (Figure 7b, iv), demonstrating selective and reversible assembly and disassembly of the triangle species. After heating the sample at 60 °C for 2 days the original distribution was largely recovered ([Pd_3_(**3**)_6_]^6+^: [Pd_4_(**3**)_8_]^8+^=3 : 4, Figure 7b, v)), although some chemical shift changes and peak broadening had occurred. The broad peaks observed in the ^1^H and ^19^F NMR spectra after irradiation are consistent with the involvement of *Z*‐**3** within the self‐assembled species (Figure 7b, iii). This effect is far more pronounced within [Pd_4_(**3**)_8_]^8+^, supporting the notion that [Pd_4_(**3**)_8_]^8+^ is more flexible and able to accommodate the mismatched ligand whereas [Pd_3_(**3**)_6_]^6+^ is more rigid and well‐defined.

The selective disassembly of [Pd_4_(**3**)_8_]^8+^ can be rationalized by considering the composition of ligands, the rate of ligand exchange for each species, and the constraints imposed on the photoswitching of ligand **3** while assembled. Variable temperature NMR experiments confirm the structures are dynamic with exchange of ligands and solvent molecules, as is common for palladium(II)‐pyridyl assemblies (see Supporting Information section 8.3).^[45]^ If photoisomerization is suppressed within the self‐assembled structures, as observed for a DTE‐based cage,^[28b]^ ligand **3** can only isomerize after dissociating from palladium. There is no evidence to suggest isomerization occurs when the ligands are coordinated to palladium (see Supporting Information Figures S74–S78 for details). For the tetrahedron [Pd_4_(**3**)_8_]^8+^, an *E*‐**3** ligand can dissociate and photoisomerize, but the newly generated *Z*‐**3** ligand cannot reassemble into the same original structure. We propose that a metastable [Pd_4_(**3**)_7_]^8+^ structure is formed and the ligands rapidly rearrange to form the double‐walled triangle, [Pd_3_(**3**)_6_]^6+^. As [Pd_3_(**3**)_6_]^6+^ is more inert, any free *E*‐**3** in solution will be kinetically trapped as [Pd_3_(**3**)_6_]^6+^. As such, irradiation with 410 nm light continuously pumps the system out‐of‐equilibrium to favor the formation of the less thermodynamically preferred [Pd_3_(**3**)_6_]^6+^. The PSS generated when irradiated with 530 nm light comprises only 20 % *E*‐**3**, which appears too low to form a significant amount of [Pd_3_(**3**)_6_]^6+^. This finding is consistent with our experiments using a sample of enriched *Z*‐**3** and palladium(II) which also resulted in the same ill‐defined mixtures.

The observed behavior is surprising as it results from a relatively small change (∼20 %) in the isomer distribution caused by irradiating with 410 nm light. Typically, stimuli responsive architectures are designed to maximize the proportion of components that are switched. This work offers a different approach, where small changes in isomer distribution can be amplified to significant changes within the system, similar to the sergeants‐and‐soldiers concept^[46]^ in self‐sorting. To the best of our knowledge, this is the first example of a self‐assembled system where the configuration can be controlled using only visible light and the resultant distribution contains the same sub‐components as the equilibrium distribution.

## Conclusion

We have shown that building visible‐light switchable *o*‐fluoroazobenzenes into palladium(II)‐pyridyl self‐assemblies leads to visible‐light responsive systems. Irradiating with visible light reversibly redistributes the sub‐components, driving the system out‐of‐equilibrium to form the higher energy, but less labile, structure. Unlike previous examples, the distinct assemblies contain the same photoisomer of the ligand. This approach of pumping systems to metastable states exploits kinetic effects to amplify small changes in photoisomer distributions to generate large changes in structural distributions.

## Conflict of interest

The authors declare no conflict of interest.

1

## Supporting information

As a service to our authors and readers, this journal provides supporting information supplied by the authors. Such materials are peer reviewed and may be re‐organized for online delivery, but are not copy‐edited or typeset. Technical support issues arising from supporting information (other than missing files) should be addressed to the authors.

Supporting Information

## Data Availability

The data that support the findings of this study are openly available in ChemRxiv at 10.33774/chemrxiv‐2021‐rfd1m, reference number 1.
