# Supplementary material for: Visible‐Light Switching of Metallosupramolecular Assemblies
Source: Chemistry. 2022 Feb 19;28(16):e202104461. doi: 10.1002/chem.202104461 (PMC9302685; doi:10.1002/chem.202104461)
Supplement: Supplementary file 1 — Supporting Information [file CHEM-28-0-s001.pdf]

Chemistry–A European Journal

Supporting Information

Visible-Light Switching of Metallosupramolecular Assemblies

Aaron D. W. Kennedy, Ray G. DiNardi, Lucy L. Fillbrook, William A. Donald, and Jonathon E. Beves*

Table of Contents

1. General Experimental ... 4
1.1. General Experimental ... 4

2. Synthesis of ligands ... 5
2.1. Synthesis of **1** ... 6
2.2. Synthesis of **2** ... 7
2.3. NMR spectra of **2** in DMSO-d_6 ... 7
2.4. Synthesis of **3** ... 8
2.5. NMR spectra of **3** in DMSO-d_6 ... 9
2.6. 2D NMR spectra of compound *E*-**3** ... 10
2.6.1. Addition of base to **3** ... 12
2.7. Identification of by-product **S1** ... 13
2.8. NMR spectra of by-product **S1** ... 14
2.9. Single crystal X-ray structural data for **S1** ... 15
2.10. Synthesis of **4** ... 16
2.11. NMR spectra of compound **4** ... 16

3. Synthesis of palladium(II) complexes ... 18
3.1. Synthesis of $[Pd(3\text{-chloropyridine})_4](OTf)_2$ ... 18
3.2. Synthesis of $[Pd(3\text{-chloropyridine})_4](BArF)_2$ ... 19
3.3. Synthesis of $[Pd(ttpy)(MeCN)](BF_4)_2$ ... 19
3.3.1. 2D NMR spectra of $[Pd(ttpy)(MeCN)](BF_4)_2$ ... 20
3.4. Synthesis of [Pd(ttpy)(*E*-**3**)]$(BF_4)_2$ ... 22
3.5. 2D NMR spectra of [Pd(ttpy)(*E*-**3**)]$(BF_4)_2$ ... 22
3.6. Synthesis of $[Pd(ttpy)(3\text{-methylpyridine})](BF_4)_2$ ... 24
3.7. 2D NMR spectra of $[Pd(ttpy)(3\text{-methylpyridine})](BF_4)_2$ ... 24

4. Photoswitching of ligand 3 ... 26
4.1. ^{1}H and ^{19}F NMR spectra of photoswitching of **3** ... 26
4.2. UV-visible absorption spectra of ligand **3** ... 27
4.3. Determination of the thermal half-life of **3** by NMR spectroscopy ... 27
4.4. The effect of palladium(II) on the switching properties of ligand **3** ... 28

5. Photoswitching of compound 4 ... 31
5.1. ^{1}H and ^{19}F NMR spectra of photoswitching of **4** ... 31
5.2. UV-visible absorption spectra of ligand **4** ... 32
5.3. Determination of the thermal half-life of **4** by UV-vis spectroscopy ... 32
5.4. The effect of palladium(II) on the switching properties of **4** ... 33

6. Formation of self-assembled products with ligand *E*-3 ... 36

7. Formation of a mixture of $[Pd_3(3)_6](BAr_F)_6$ and $[Pd_4(3)_8](BAr_F)_8$ ... 38

8. NMR characterization of $[Pd_3(3)_6](BF_4)_6$ and $[Pd_4(3)_8](BF_4)_8$ 39
8.1. ^1H, ^{13}C and ^{19}F NMR Spectra 39
8.2. ^1H diffusion NMR spectroscopy of a mixture of $[Pd_3(\mathbf{3})_6]^{6+}$ and $[Pd_4(\mathbf{3})_8]^{8+}$ 43
8.3. Variable temperature ^1H NMR data of $[Pd_3(\mathbf{3})_6]^{6+}$ and $[Pd_4(\mathbf{3})_8]^{8+}$ in DMSO-d_6 45
9. ESI-MS characterization of $[Pd_3(3)_6](BF_4)_6$ and $[Pd_4(3)_8](BF_4)_8$ 46
9.1. ESI-MS isotope patterns of $[Pd_3(\mathbf{3})_6]^{6+}$ 46
9.2. ESI-MS isotope patterns of $[Pd_4(\mathbf{3})_8]^{8+}$ 47
10. Assigned characterisation of $[Pd_3(3)_6]^{6+}$ 48
10.1. HR-ESI-MS for $([Pd_3(\mathbf{3})_6](BF_4)_{(6-n)})^{n+}$ 48
11. Assigned characterisation of $[Pd_4(3)_8]^{8+}$ 49
11.1. HR-ESI-MS for $([Pd_4(\mathbf{3})_8](BF_4)_{(8-n)})^{n+}$ 50
12. Controlled degradation of self-assembled species with DMAP 51
13. Formation of self-assembled products upon combination of $[Pd(CH_3CN)_4](BF_4)_2$ and *Z*-352
14. Binding measurements between palladium(II) and 3-pyridyl ligands 54
14.1. Determination of binding constants using ITC 54
14.2. Competitive binding between ligand **3** and 3-methylpyridine with palladium(II) 57
15. Photoswitching of self-assembled structures 58
15.1. Monitoring of a mixture of $[Pd_3(\mathbf{3})_6]^{6+}$ and $[Pd_4(\mathbf{3})_8]^{8+}$ using UV-vis spectroscopy 58
15.2. Monitoring of a mixture of $[Pd_3(\mathbf{3})_6]^{6+}$ and $[Pd_4(\mathbf{3})_8]^{8+}$ using NMR spectroscopy 59
15.3. Mass spectrometry data during photoswitching 62
16. References 63

1. General Experimental

1.1. General Experimental

All data for this project is freely available online on the ChemRxiv server: Kennedy, A.; DiNardi, R.; Fillbrook, L.; Donald, W.; Beves, J. ChemRxiv 2021, 10.33774/chemrxiv-2021-rfd1m. That content is a preprint and has not been peer-reviewed.

Due to a series of lockdowns related to the Covid-19 outbreak in Sydney during 2021, our access to the laboratory was severely limited. For this reason, some of the reaction times are long (3 days, 10 days etc) as it was not possible to conduct experiments on consecutive days. We believe most of these experiments are complete within 1 day, but report the longer times we have actually conducted our measurements.

Reagents and solvents were purchased from Sigma-Aldrich, Merck, Chem Supply, Combi-Blocks or Alfa Aeser, and were used without purification unless stated otherwise.

NMR spectroscopy was performed using a Bruker Avance III 400 with a Prodigy CryoProbe, a Bruker Avance III 500, a Bruker Avance III 600 or a Bruker Avance III HD 600 with a TCI CryoProbe. Samples were prepared using either DMSO-d_6 or $CDCl_3$, purchased from Cambridge Isotope Laboratories, Inc. $CDCl_3$ was stored over K_2CO_3 to remove traces of acid. All chemical shifts were calibrated against residual solvent signals. All coupling constants (*J*) are reported in Hertz. Signals in the NMR spectra are reported as broad (br), singlet (s), doublets (d), triplets (t), quartets (q), quintets (qu), sextets (sx), septets (sept), or unclear multiplets (m). NMR spectra were processed with MestReNova 12.0.0 software. All NMR data are assigned unambiguously, except where specified.

UV-Vis experiments were performed on an Agilent Cary 60 Bio UV-Visible Spectrophotometer equipped with a customized Cary Single Cell Peltier Accessory, keeping the samples at 25 °C unless stated otherwise. The cell holder was modified to allow for irradiation perpendicular to the direction of measurement, as previously described.[4] The LED was mounted on a heat sink positioned 4 cm away from the cell and the samples were stirred to ensure homogeneity. The LED emissions were measured using an Ocean Optics HR4000 high-resolution spectrometer.

NMR irradiations were performed *ex situ* using the LEDs described below, unless stated otherwise. The LED was mounted on a heat sink positioned 4 cm from the NMR tube and spectra collected until a photostationary state was achieved.

ITC experiments were performed using the TAM IV Isothermal Microcalorimeter (TA Instruments) thermostat system equipped with the TAM Nanocalorimeter. Injections were performed using the TAM dual syringe pump. Thermograph data were processed using NanoAnalyse software (TA Instruments).

High-resolution mass spectrometry (HR-MS) experiments were performed on a hybrid linear quadrupole ion trap mass spectrometer (Thermo LTQ Orbitrap XL) equipped with an external nanospray ionization (NSI) source. Metal-templated structures experiments were performed using undiluted NMR samples in DMSO-d_6.

Computational models of the self-assembled structures were optimized using the Merck Molecular Force Field (MMFF) method in Spartan Student v8 and are only topological representations.

Table S1 Specifications of the LEDs used in this work

LED Part Number (Supplier)	Dominant Wavelength		
	Minimum	Maximum	Typical
A00-UV410-48 (SemiLEDs)	410	420	410
LXML-PM01-0100 (LumiLEDs)	520	540	530
LXML-PX02-0000 (LumiLEDs)	566	569	567

2. Synthesis of ligands

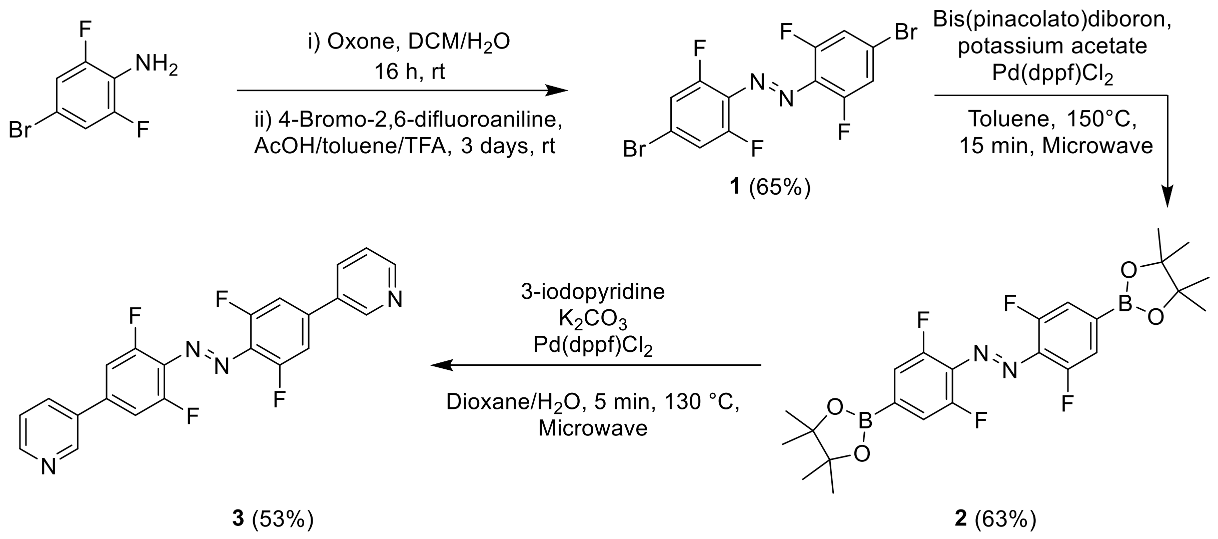

Scheme S1 Overall synthetic scheme for compound 3.

2.1. Synthesis of 1

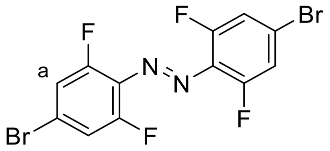

The title compound was synthesized by a modified literature procedure.[1] 4-Bromo-2,6-difluoroaniline (7.12 g, 34.2 mmol) dissolved in DCM (100 mL) was added to Oxone (42.3 g, 68.8 mmol) dissolved in H_2O (400 mL) and stirred vigorously for 16 h, over which time the organic layer turned dark green. The layers were separated and the organic layer washed with aq. HCl (1 M, 100 mL), saturated aq. $NaHCO_3$ (2 × 100 mL), H_2O (100 mL), brine (100 mL) and dried over $MgSO_4$. The solvent was removed under reduced pressure to give a greenish solid. This was dissolved in toluene (60 mL) and 4-bromo-2,6-difluoroaniline (7.12 g, 34.2 mmol) added. A mixture of acetic acid (60 mL) and trifluoroacetic acid (10 mL) was added and the mixture stirred for 3 days, giving a deep red solution, The solvent was removed under reduced pressure and the crude solid purified by recrystallisation from EtOAc:hexanes to give **1** as red needles (9.26 g, 65%). Spectral data was identical to that previously reported.[2]
^{1}H NMR (400 MHz, DMSO-*d₆*, 298 K) δ 7.79 (d, J = 8.6 Hz, 4H, H^a).
^{19}F NMR (376 MHz, DMSO-*d₆*, 298 K) δ -119.33 (d, J = 9.9 Hz).

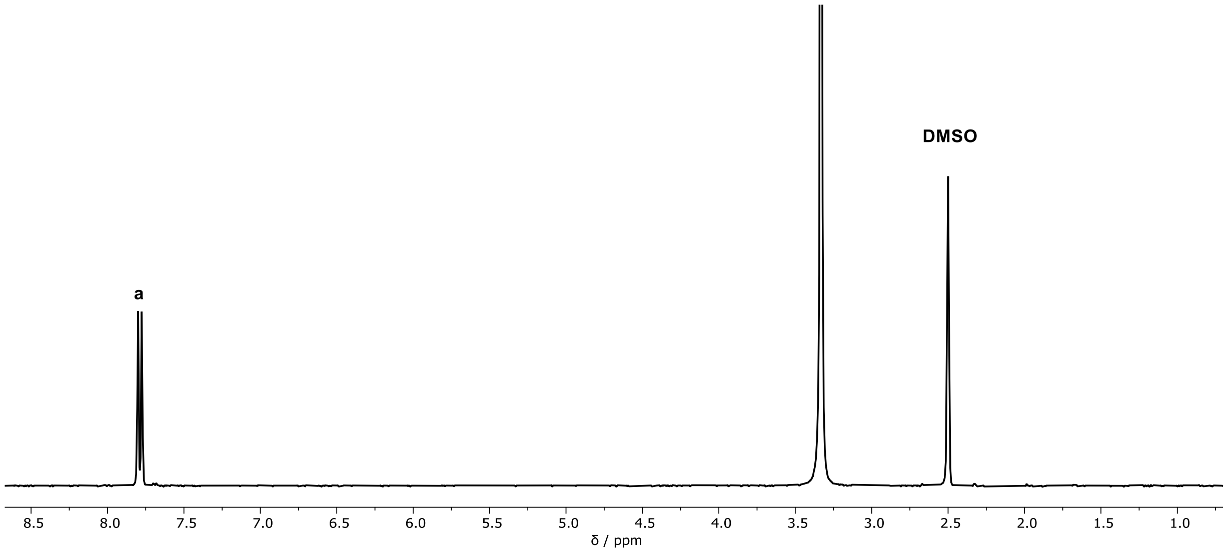

Figure S1 ^{1}H NMR (400 MHz, DMSO-d_6, 298 K) spectrum of **1**.

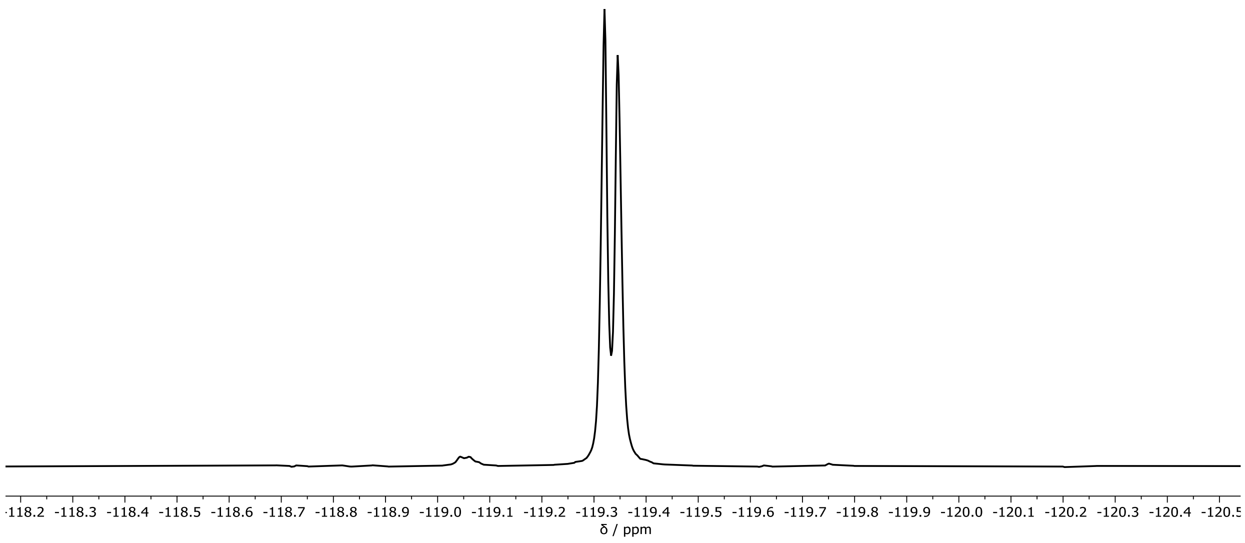

Figure S2 Partial ^{19}F NMR (377 MHz, DMSO-d_6, 298 K) spectrum of **1**.

2.2. Synthesis of 2

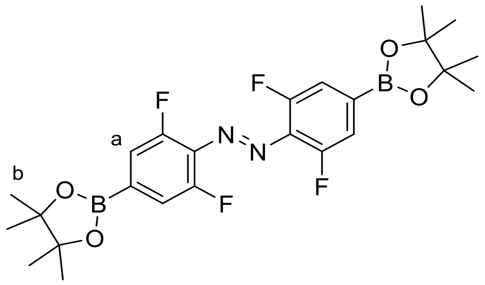

The title compound was synthesized by a modified literature procedure.[3] Compound **2** (500 mg, 1.21 mmol), bis(pinacolato)diboron (678 mg, 2.67 mmol) and potassium acetate (724 mg, 7.40 mmol) were combined in toluene (8 mL) and degassed with Ar for 30 minutes. Pd(dppf)Cl_2 (35 mg, 0.05 mmol) was added and the red suspension further degassed for 5 minutes. The mixture was heated in a microwave reactor at 150 °C for 15 minutes. EtOAc (50 mL) was added and the suspension filtered through Celite. The combined organics were washed with water (75 mL), brine (75 mL) and dried over $MgSO_4$. The solvent was removed under reduced pressure to give a red solid. The residue was triturated with cold MeCN (5 mL) to give **2** as a deep red solid (385 mg, 63%).
Spectral data was identical to that previously reported.[3]
^{1}H NMR (400 MHz, DMSO-*d6,* 298 K) δ 7.48 (d, *J* = 9.7 Hz, 4H), 1.33 (s, 24H)
^{19}F NMR (376 MHz, DMSO-*d6*, 298 K) δ -121.93 (d, *J* = 10.0 Hz).

2.3. NMR spectra of 2 in DMSO-d6

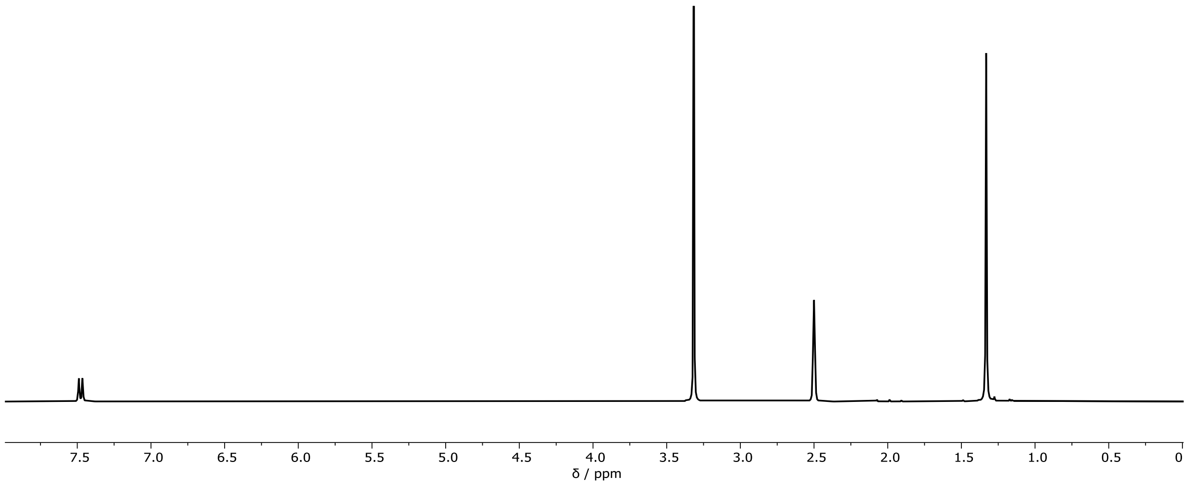

Figure S3 ^{1}H NMR (400 MHz, DMSO-*d6*, 298 K) spectrum of **2**.

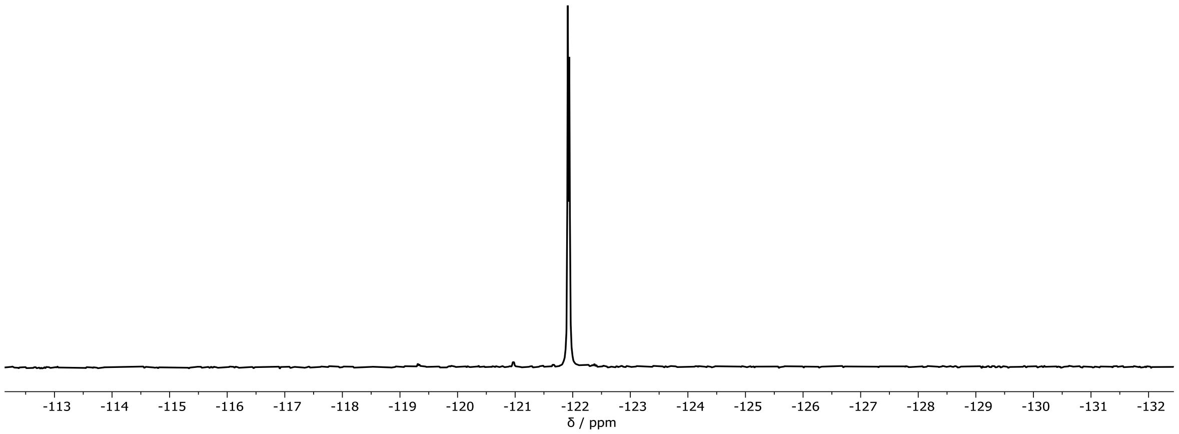

Figure S4 ^{19}F NMR (376 MHz, DMSO-*d₆*, 298 K) spectrum of **2.**

2.4. Synthesis of 3

Compound **2** (150 mg, 0.30 mmol) and 3-iodopyridine (410 mg, 2.0 mmol) were dissolved in dioxane (10 mL) in a microwave tube. K_2CO_3 (105 mg, 0.76 mmol) dissolved in H_2O (2 mL) was added. The suspension was degassed with Ar for 20 minutes. Pd(dppf)Cl_2 (13 mg, 0.01 mmol) was added, and the suspension was further degassed for 5 minutes. The mixture was heated in a microwave reactor at 130 °C for 5 minutes giving a deep red solution. The solution was filtered through Celite to removed insoluble impurities. The Celite was washed with CH_2Cl_2 (100 mL) and the organic layer was washed with H_2O (2 × 50 mL), brine (50 mL) and dried over $MgSO_4$. The organic solution was filtered, and the solvent was removed under reduced pressure. The resulting solid was purified by being dissolved in CH_2Cl_2/MeCN (9:1). The more volatile CH_2Cl_2 was removed under reduced pressure before the solution was cooled to 0 ºC in an ice bath. The resulting precipitate was collected affording the **3** (as a mix of isomers) as a red solid (64 mg, 0.16 mmol, 53 %).

HR-ESI-MS *m/z* 409.1020 $[M+H]^+$ requires 409.1070.

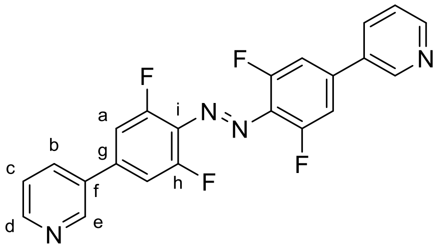

E-**3**

^{1}H NMR (400 MHz, DMSO-d_6) δ 9.11 (d, *J* = 2.4 Hz, 2H, H^e), 8.69 (dd, *J* = 4.7, 1.5 Hz, 2H, H^d), 8.31 (ddd, *J* = 8.2, 1.9, 1.9 Hz, 2H, H^b), 7.91 (d, J_{HF} = 11.0 Hz, 4H, H^a), 7.58 (dd, *J* = 8.1, 4.8 Hz, 2H, H^c).

^{19}F NMR (564.6 MHz, DMSO-d_6) δ -120.1 (d, J_{HF} = 11.8 Hz)

^{13}C NMR (151 MHz, DMSO-d_6) δ 155.3 (d, *J* = 259 Hz, C^h), 150.3 (C^d), 148.0 (C^e), 141.9 (C^g), 134.6 (C^b), 132.2 (C^f), 130.0 (C^i), 124.0 (C^c), 111.5 (d, *J* = 22 Hz, C^a).

UV-Vis (DMSO) : visible λ_{max}/nm 466 (ε = 2.1 × 10^3 M^{-1} cm^{-1})

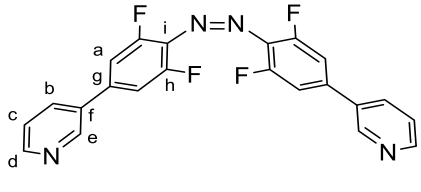

Z-**3**

^{1}H NMR (400 MHz, DMSO-d_6) δ 8.99 (d, *J* = 2.5 Hz, 2H, H^e), 8.62 (dd, *J* = 4.8, 1.5 Hz, 2H, H^d), 8.19 (ddd, *J* = 8.2, 1.9, 1.9 Hz, 2H, H^b), 7.79 (d, *J* = 9.9 Hz, 4H, H^a), 7.50 (dd, *J* = 8.1, 4.8 Hz, 2H, H^c).

^{19}F NMR (376 MHz, DMSO-d_6) δ -119.7 (d, J_{HF} = 9.6 Hz)

UV-Vis (DMSO): visible λ_{max}/nm 432 (ε = 2.8 × 10^3 M^{-1} cm^{-1})

2.5. NMR spectra of 3 in DMSO-d6

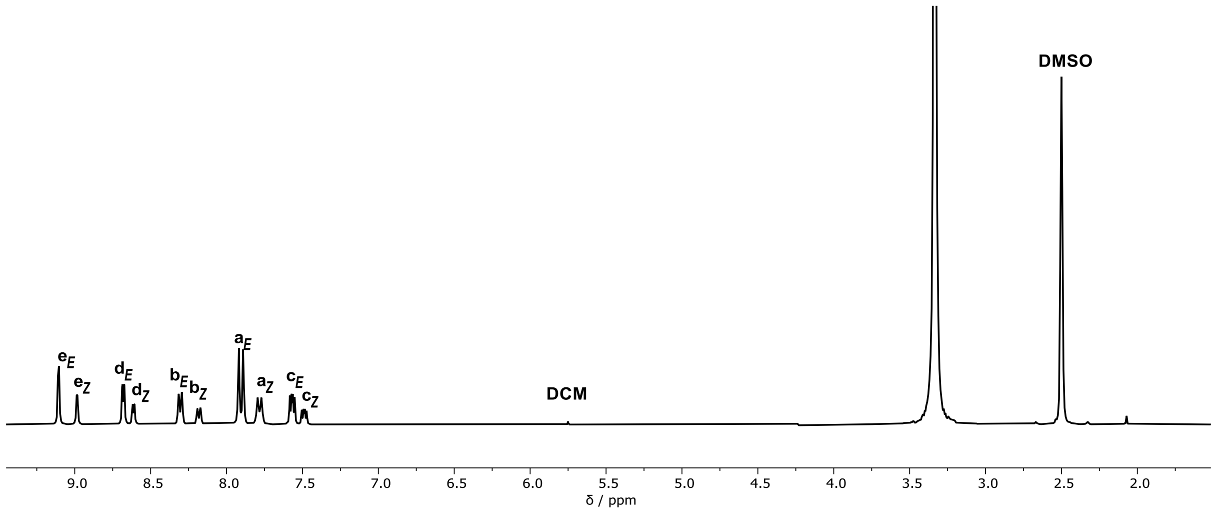

Figure S5 ^{1}H NMR (400 MHz, DMSO-d$_6$, 298 K) spectrum of a mixture of *E*-**3** and *Z*-**3**.

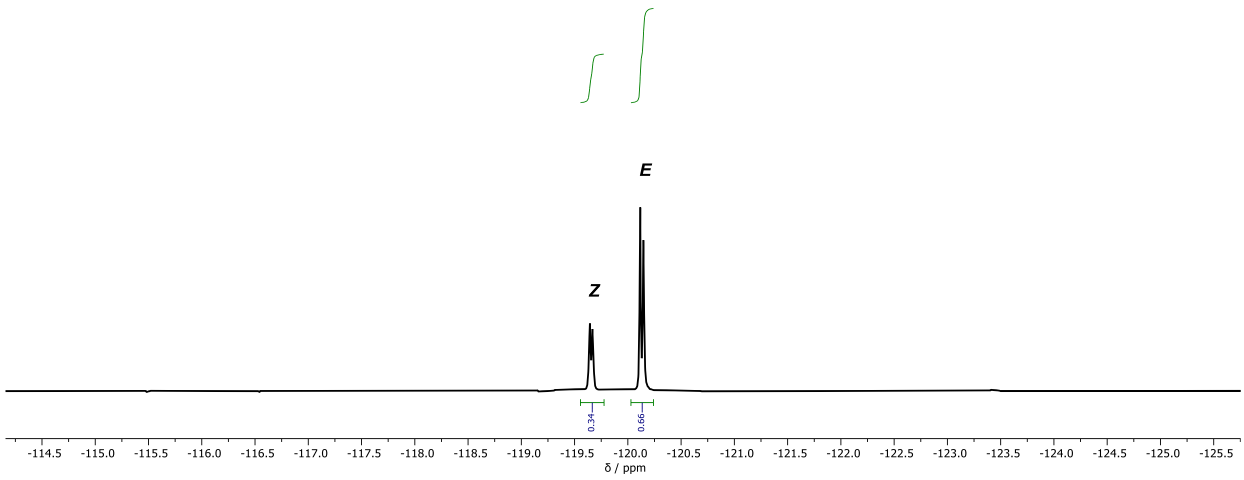

Figure S6 Partial ^{19}F NMR (376 MHz, DMSO-d$_6$, 298 K) spectrum of a of mixture of *E*-**3** and *Z*-**3**.

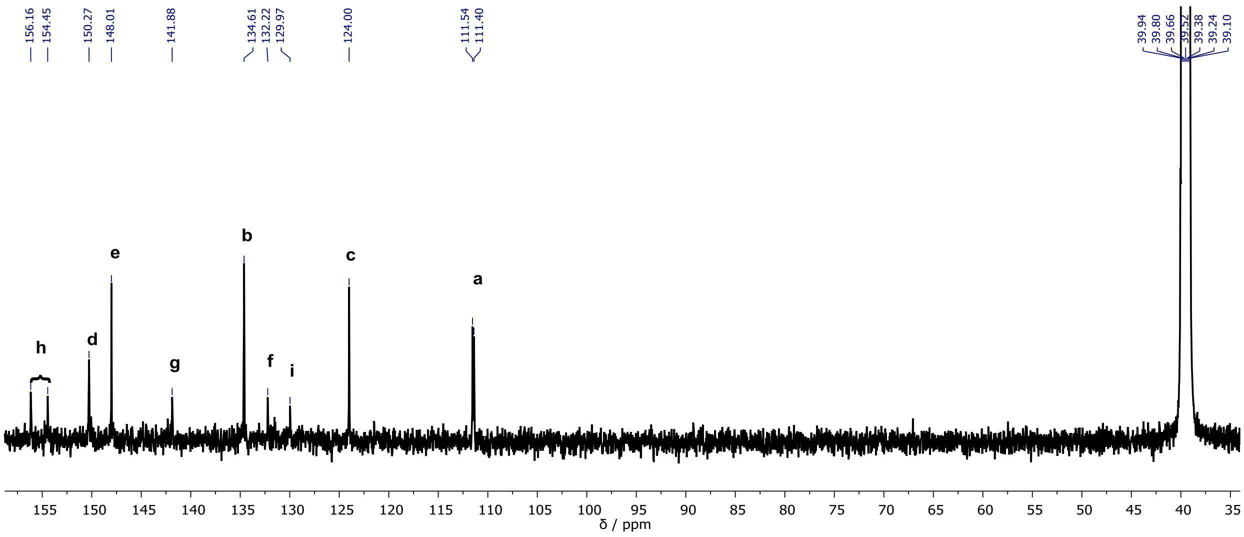

Figure S7 Partial ^{13}C{^{1}H} NMR (151 MHz, DMSO-d$_6$, 298 K) spectrum of *E*-**3**.

2.6. 2D NMR spectra of compound *E*-3

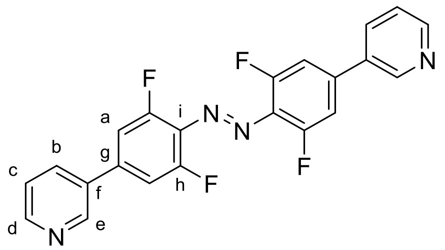

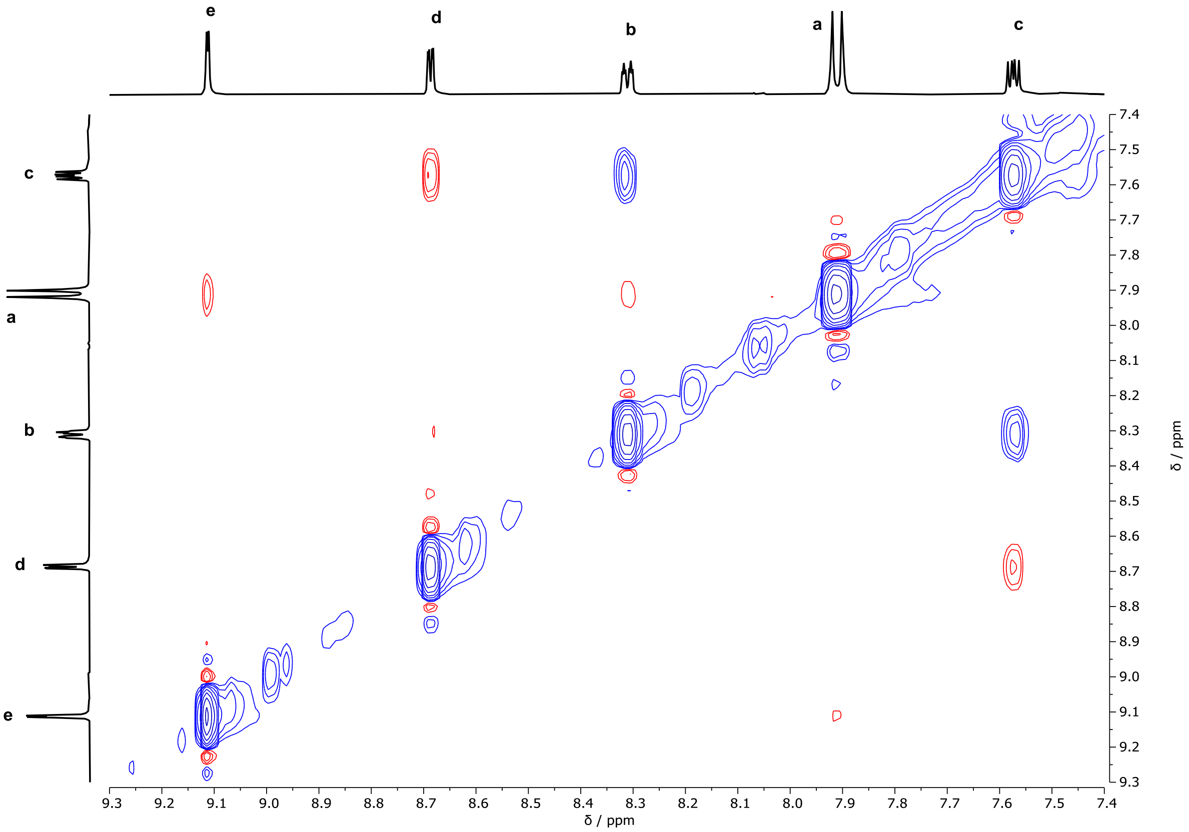

Figure S8 ^{1}H-^{1}H NOESY (600 MHz, DMSO-d_6, 298 K) spectrum of *E*-**3**.

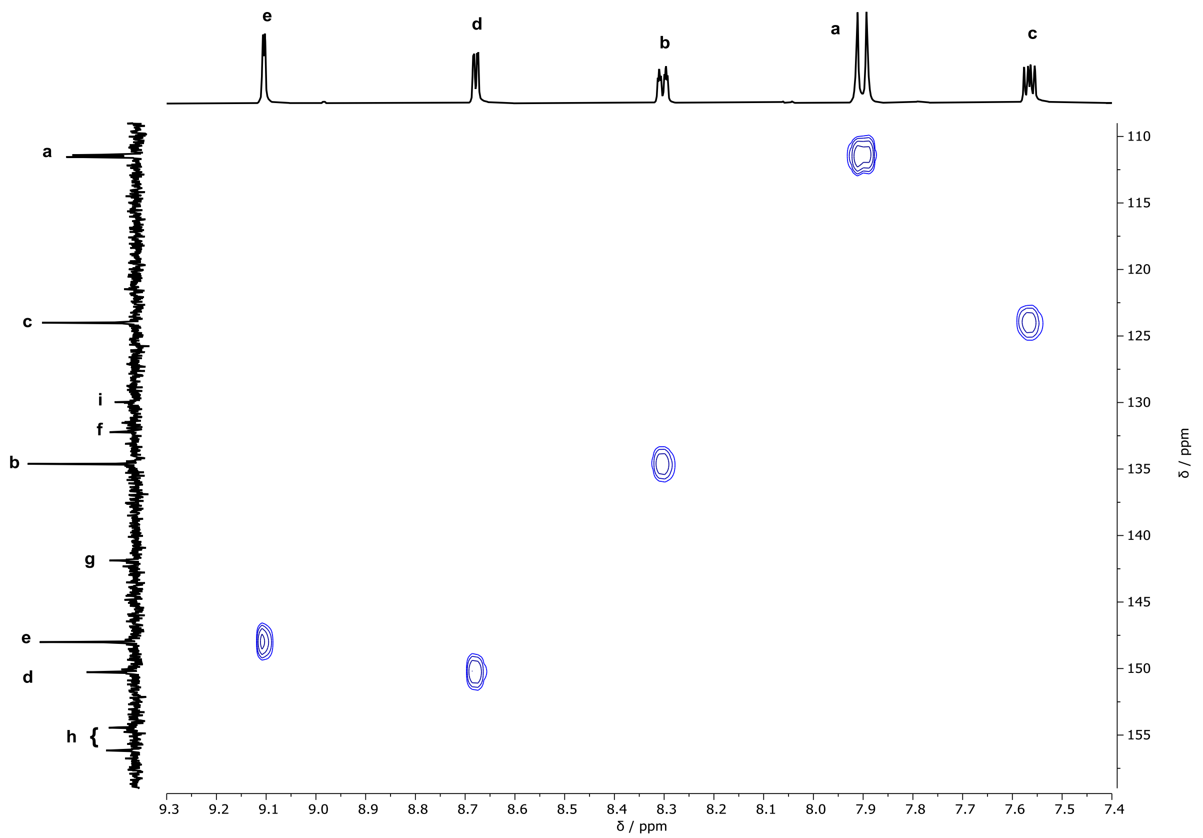

Figure S9 ^{1}H-^{13}C HSQC (600 MHz, DMSO-d_6, 298 K) spectrum of *E*-**3**.

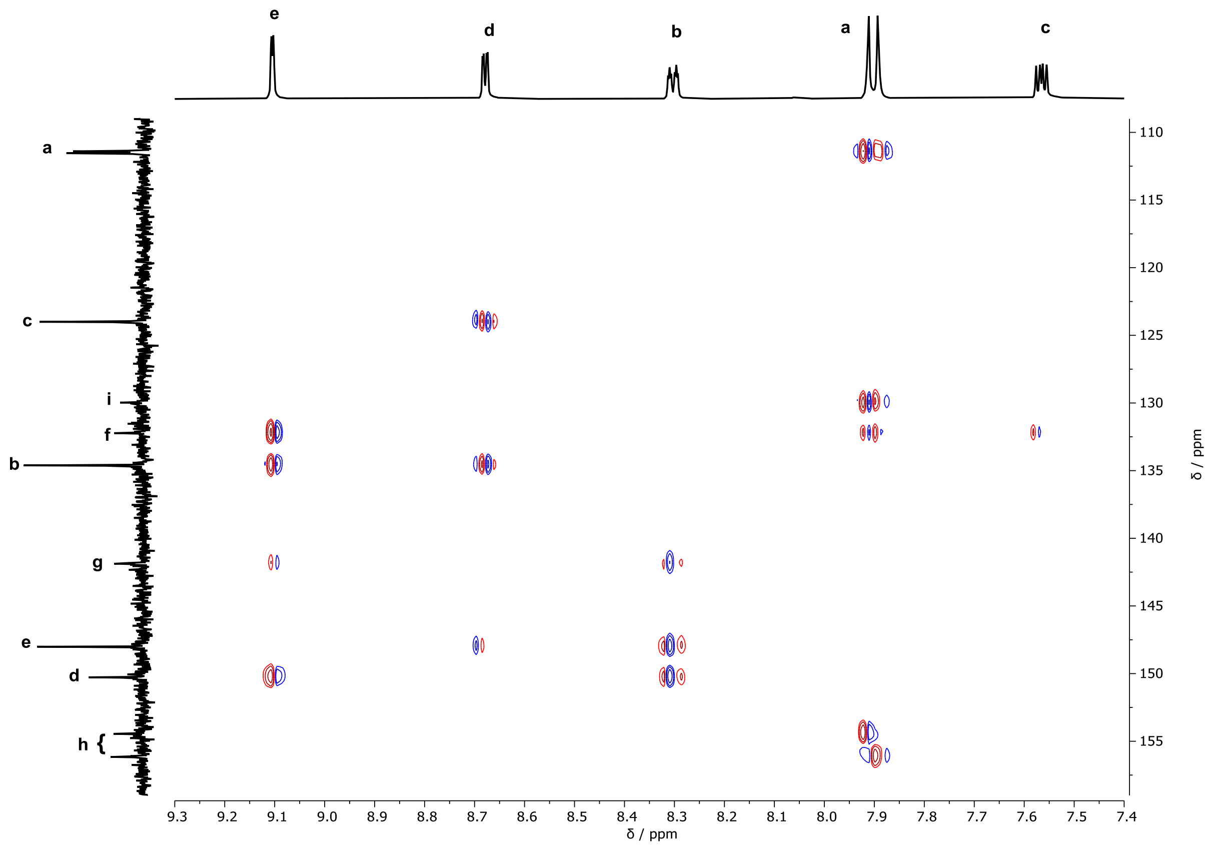

Figure S10 ^{1}H-^{13}C HMBC (600 MHz, DMSO-d_6, 298 K) spectrum of *E*-**3**.

2.6.1. *Addition of base to **3***

To see whether **3** was protonated upon isolation, an ^{1}H NMR spectrum was acquired before and after the addition of solid K_2CO_3 as a base to a sample of compound **3**. No change in the chemical shift for the NMR signals could be observed after the addition of K_2CO_3, indicating that product **3** is not isolated as a protonated species.

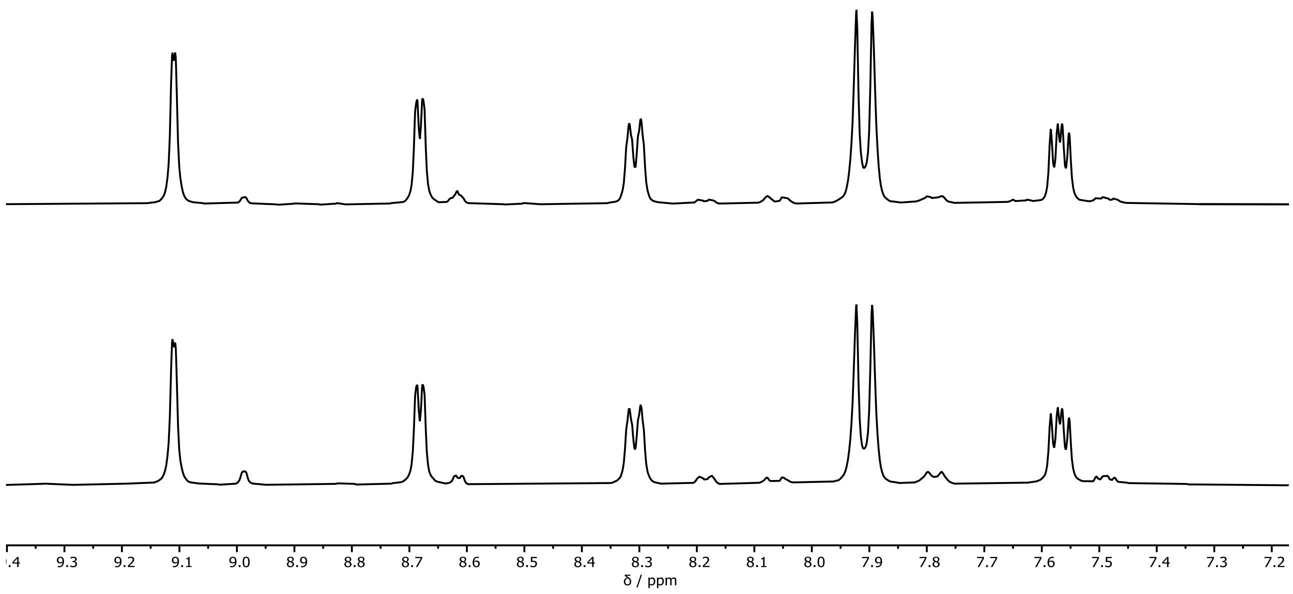

Figure S11 ^{1}H NMR (400 MHz, DMSO-d_6, 298 K) spectra of a) compound **3** and b) compound **3** + solid K_2CO_3.

2.7. Identification of by-product S1

While optimizing the synthesis of **3** the monosubstituted product shown in Scheme S2 was isolated in 31% yield. The structure was confirmed by single crystal X-ray diffraction, suggesting the second Suzuki coupling was unfavorable when using the pyridyl boronic acid.

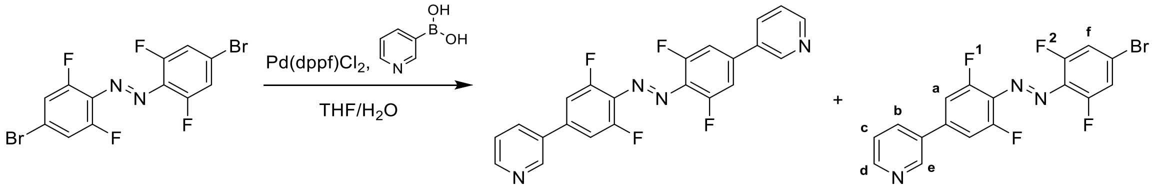

Scheme S2 Suzuki coupling of **1** to give the unsymmetric product **S1**.

Compound **1** (200 mg, 0.49 mmol) and pyridin-3-ylboronic acid (132 mg, 1.08 mmol) were combined in THF (14 mL). K_2CO_3 (114 mg, 1.08 mmol) dissolved in H_2O (1 mL) was added, and the suspension was degassed with Ar for 20 minutes. Pd(dppf)Cl_2 (63 mg, 0.09 mmol) was added, and the suspension was further degassed for 5 minutes. The mixture was heated at 70 °C for 36 hours giving a deep red solution. The solution was filtered through Celite and extracted with DCM (40 mL). The organic layer was washed with H_2O (2 × 50 mL), brine (50 mL) and dried over $MgSO_4$. The solvent was removed under reduced pressure and the crude product purified by column chromatography. The column was deactivated with 3 column volumes of 10% TEA in hexanes and the compound eluted using a gradient of 25% EtOAc/hexanes to 50% EtOAc/hexanes to give **S1** as a red solid (62 mg, 31%).

^{1}H NMR (400 MHz, $CDCl_3$) δ 8.90 (s, 1H, H^e), 8.70 (d, *J* = 4.8 Hz, 1H, H^d), 7.93 (ddd, *J* = 8.0, 1.9 Hz, 1H, H^b), 7.46 (dd, *J* = 8.0, 4.8 Hz, 1H, H^c), 7.34 – 7.27 (m, 4H, H^{a+f}).
^{19}F NMR (564.60 MHz, DMSO-*d*$_6$) δ -118.7 (d, J_{HF} = 8.6 Hz, F^2), -118.8 (d, J_{HF} = 10.3 Hz , F^1).

2.8. NMR spectra of by-product S1

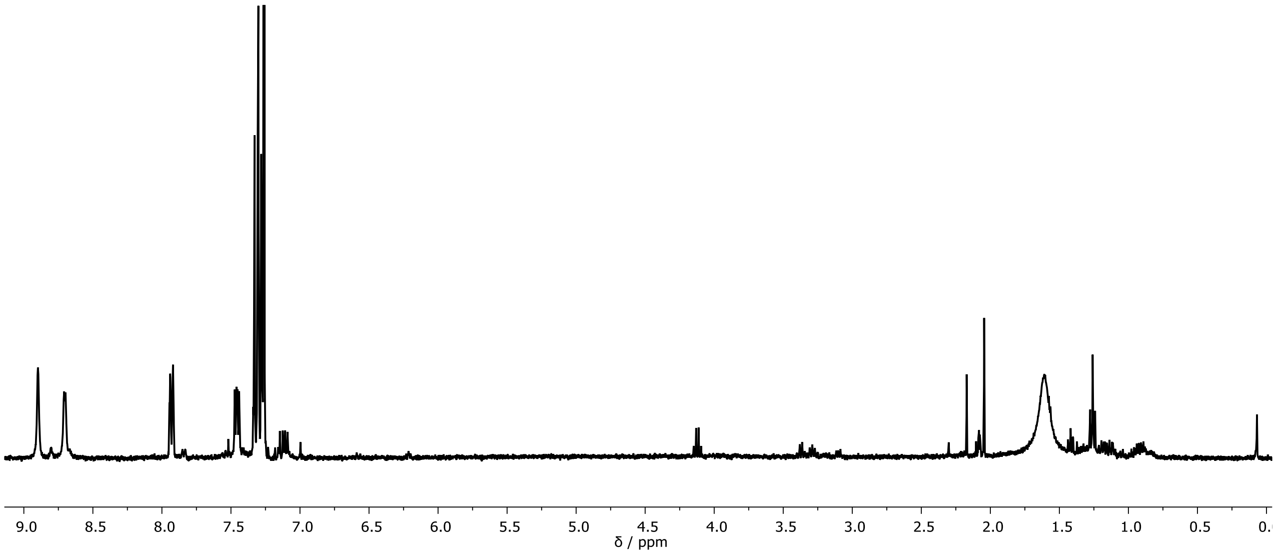

Figure S12 ^{1}H NMR spectrum (400 MHz, $CDCl_3$, 298 K) of crude product **S1**.

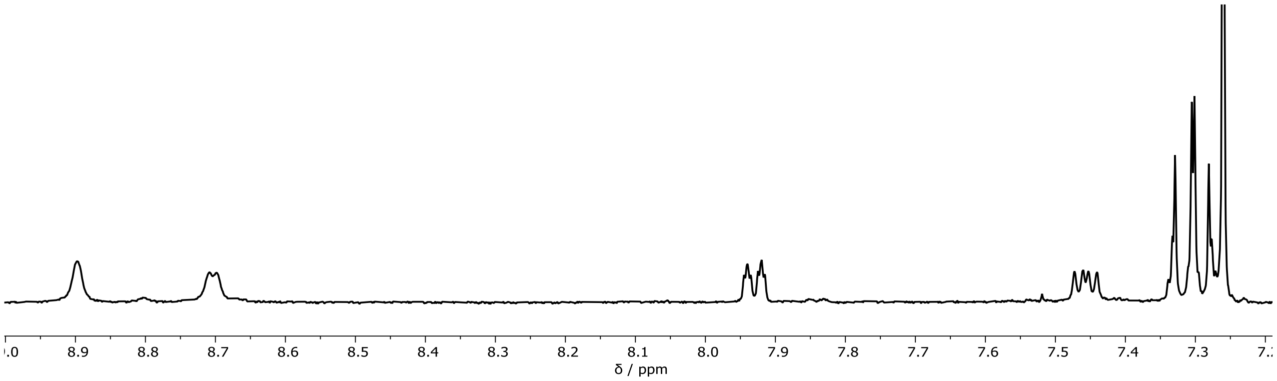

Figure S13 Partial ^{1}H NMR spectrum (400 MHz, $CDCl_3$, 298 K) of crude product **S1**.

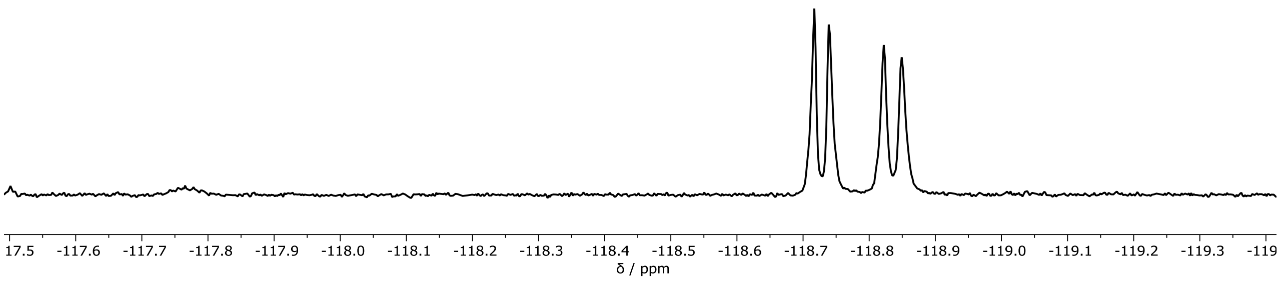

Figure S14 Partial ^{19}F NMR spectrum (376.50 MHz, $CDCl_3$, 298 K) of crude product **S1**.

2.9. Single crystal X-ray structural data for S1

All structures were deposited with the Cambridge Structural Database (CCDC 2091395)

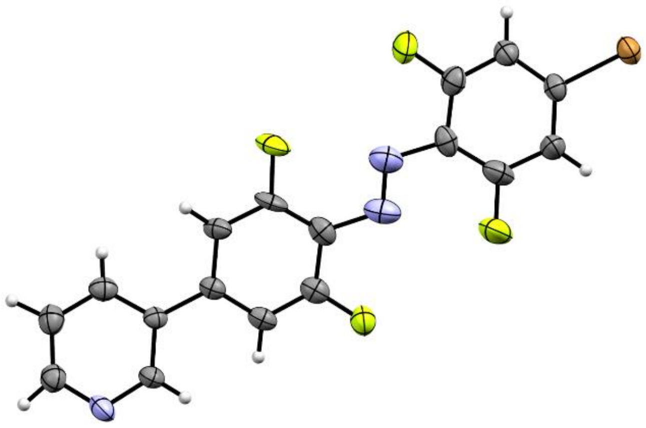

Figure S15 Single crystal X-ray structure of unsymmetric pyridine substituted derivative azobenzene **S1**. An ORTEP representation of the asymmetric unit in the X-ray crystal structure of **S1**. Thermal ellipsoids are drawn at 50% probability.

Pale yellow plate-like crystal of $C_{17}H_8BrF_4N_3$ were grown by slow evaporation of $CDCl_3$. A single crystal with dimensions of 0.013 × 0.045 × 0.254 mm was selected under the polarizing microscope (Leica M165Z). A MicroLoop (MiTeGen, USA) consisting of a thin polymer tip with a loop was used to pick up the single crystal, coated with NVH type immersion oil, which was then mounted on the goniometer using a cryo loop for intensity measurements and transferred to the cold nitrogen stream generated by an Oxford Cryostream 800 series. The X-ray diffraction measurements were carried out at 150 K on a Bruker D8 Quest Single Crystal diffractometer using Incoatec IµS 3.0 Microfocus Source with Mo-Kα radiation (λ = 0.710723 Å). Symmetry related absorption corrections using the program SADABS[4] were applied and the data were corrected for Lorentz and polarisation effects using Bruker APEX3 software.[5] The structure was solved with SHELXT[6] (intrinsic phasing) and the full-matrix least-square refinements were carried out using SHELXL-2014[6] through Olex2[7] suite of software. The non-hydrogen atoms were refined anisotropically.

Crystal Data for $C_{17}H_8BrF_4N_3$ (M =410.17 g/mol): triclinic, space group P-1 (no. 2), a = 7.3829(11) Å, b = 8.2163(12) Å, c = 13.213(2) Å, α = 93.791(5)°, β = 105.633(5)°, γ = 99.683(5)°, V = 755.6(2) Å^3, Z = 2, T = 150.0 K, μ(MoKα) = 2.770 mm^{-1}, $Dcalc$ = 1.803 g/cm^3, 8889 reflections measured (5.064° ≤ 2Θ ≤ 55.084°), 3431 unique (R_{int} = 0.0954, R_{sigma} = 0.1220) which were used in all calculations. The final R_1 was 0.0621 (I > 2σ(I)) and wR_2 was 0.1482 (all data).

Deposition numbers 2091395 (for compound **S1**) contains the supplementary crystallographic data for this paper. These data are provided free of charge by the joint Cambridge Crystallographic Data Centre and Fachinformationszentrum Karlsruhe Access Structures service.

2.10. Synthesis of 4

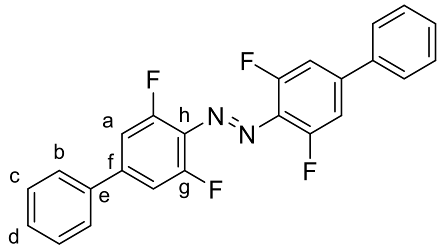

Compound **2** (150 mg, 0.30 mmol), iodobenzene (133 mg, 73 µL, 0.66 mmol) and K_2CO_3 (164 mg, 1.2 mmol) were combined in dioxane (8 mL) and the suspension was degassed with N_2 for 20 minutes. Pd(dppf)Cl_2 (15 mg, 0.018 mmol) was added and the suspension was further degassed for 5 minutes. The mixture was heated at 60 °C for 18 hours giving a deep red solution. The solution was extracted with $CHCl_3$ (40 mL) and the organic layer was washed with H_2O (2 × 25 mL), brine (25 mL) and dried over $MgSO_4$. The solvent was removed under reduced pressure and the crude product purified by column chromatography (SiO_2, Hexanes/EtOAc (9:1)) to give **4** as a red solid (23 mg, 20%).

^{1}H NMR (600 MHz, DMSO-*d$_6$*) δ 7.89 (d, *J* = 7.4 Hz, 4H, H^b), 7.77 (d, *J* = 11.7 Hz, 4H, H^a), 7.54 (dd, *J* = 7.4 Hz, 4H, H^c), 7.49 (dd, *J* = 7.3 Hz, 2H, H^d).

^{13}C NMR (151 MHz, DMSO-*d$_6$*) δ 155.8 (dd, *J* = 259, 6 Hz, C^g), 145.2 (C^f), 137.0 (C^e), 130.0 (C^{d+h}), 129.7 (C^c), 127.5 (C^b)z, 111.4 (d, *J* = 22 Hz)

^{19}F NMR (564.6 MHz, DMSO-*d$_6$*) δ -120.3 (d, *J* = 11.7 Hz).

UV-Vis (DMSO): visible λ_{max}/nm 462 (ε = 3.3 × 10^3 M^{-1} cm^{-1})

2.11. NMR spectra of compound 4

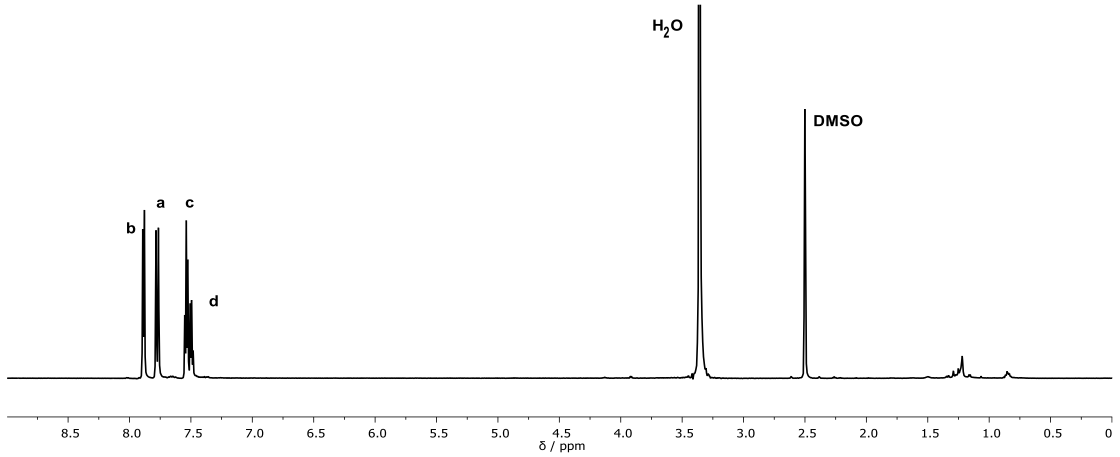

Figure S16 ^{1}H NMR (600 MHz, DMSO-*d$_6$*, 298 K) spectrum of *E*-**4**.

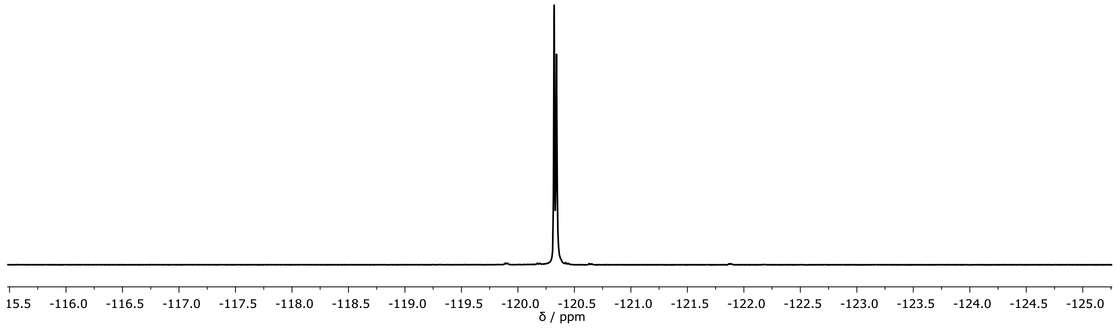

Figure S17 ^{19}F NMR (564.6 MHz, DMSO-*d$_6$*, 298 K) spectrum of *E*-**4**.

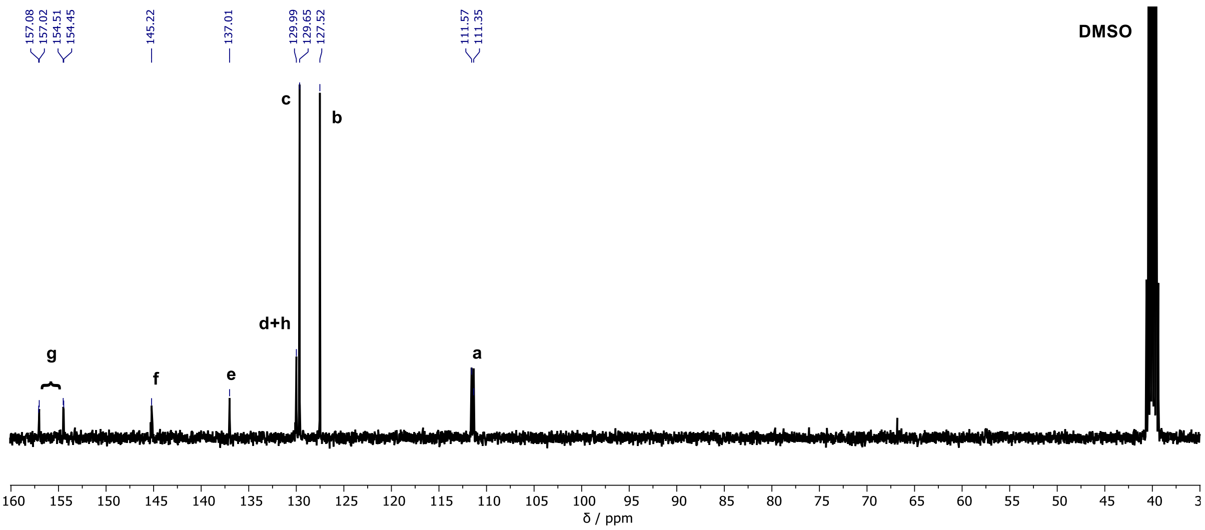

Figure S18 $^{13}C\{^{1}H\}$ NMR (100.6 MHz, DMSO-d_6, 298 K) spectrum of *E*-**4**.

3. Synthesis of palladium(II) complexes

3.1. Synthesis of [Pd(3-chloropyridine)$_4$](OTf)$_2$

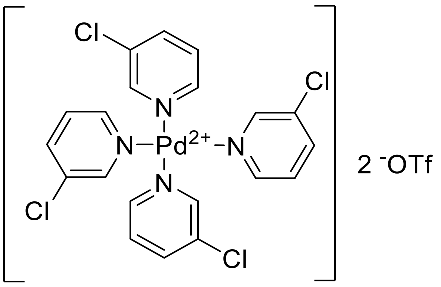

Title molecule was prepared following literature procedure.[8]

A solution of $PdCl_2$ (348 mg, 1.96 mmol) and AgOTf (1.06 g, 4.14 mmol) in CH_3CN (4 mL) was stirred at 80 °C for 2 h. The AgCl precipitate was removed by centrifugation, and the precipitate was washed with CH_3CN. The obtained solution was concentrated under reduced pressure. CH_3NO_2 (4 mL) and 3-chloropyridine (Py*) (1.79 g, 1.50 ml, 15.8 mmol) were added to the residue. The solution was stirred at 80 °C for 1 h and concentrated in vacuo. The residue was washed with a small amount of $CHCl_3$ to remove residual Py* and with water to remove residual AgOTf, and dried in vacuo to afford Pd(Py*)$_4$(OTf)$_2$ as a yellow solid (926 mg, 1.12 mmol, 58%). Spectral data was identical to that previously reported.[8]

^{1}H NMR (400 MHz, NO_2CD_3) δ 9.10 (d, J = 2.2 Hz, 4H), 9.01 (dd, J = 5.7, 1.2 Hz, 4H), 8.04 (ddd, J = 8.4, 2.2, 1.2 Hz, 4H), 7.59 (dd, J = 8.4, 5.7 Hz, 4H).

3.2. Synthesis of [Pd(3-chloropyridine)$_4$](BArF)$_2$

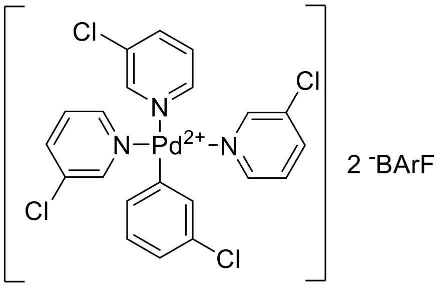

Title molecule was prepared following literature procedure.[9] (BArF = tetrakis[3,5-bis(trifluoromethyl)phenyl]borate).
[PdPy*$_4$](OTf)$_2$ (150 mg, 0.175 mmol) and NaBArF (311 mg, 0.351 mmol) were combined in a reaction flask and dry CH_2Cl_2 (30 mL) was added. The reaction mixture was sonicated for 10 min at rt and filtered to remove insoluble NaOTf. The filter was washed with CH_2Cl_2 (60 mL) and the filtrate was evaporated under reduced pressure to dryness to obtain [PdPy*$_4$](BArF)$_2$ as an off-white foam (305 mg, 0.133 mmol, 76%). Spectral data was identical to that previously reported.[9]

^{1}H NMR (400 MHz, NO_2CD_3) δ 8.89 (d, *J* = 2.2 Hz, 4H), 8.83 (dd, *J* = 5.8, 1.2 Hz, 4H), 8.07 (ddd, *J* = 8.4, 2.2, 1.2 Hz, 4H), 7.84 (dt, *J* = 5.1, 2.2 Hz, 16H), 7.67 (s, 8H), 7.59 (dd, *J* = 8.4, 5.7 Hz, 4H).

3.3. Synthesis of [Pd(ttpy)(MeCN)](BF$_4$)$_2$

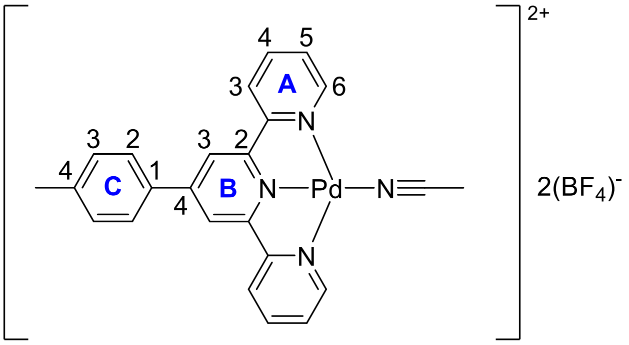

4'-(*p*-tolyl)-2,2':6',2''-terpyridine was prepared following a previously reported procedure.[10]
Tetrakis(acetonitrile)palladium(II) tetrafluoroborate (100 mg, 0.31 mmol) was added to a solution of 4'-(*p*-tolyl)-2,2':6',2''-terpyridine (137 mg, 0.31 mmol) in CH3CN (20 mL). The solution was stirred at r.t. for 24 h. The solution was concentrated under reduced pressure. Et2O was added to cause precipitation of the product. The suspension was filtered and the solid was collected and washed with Et2O, affording the product as an off-white solid (99.1 mg, 0.15 mmol, 50 %).
^{1}H NMR (500 MHz, DMSO) δ 8.99 (s, 2H, H^{B3}), 8.91 (d, *J* = 8.0 Hz, 2H, H^{A3}), 8.67 – 8.49 (m, 4H, H^{A4} + H^{A6}), 8.17 (d, *J* = 8.0 Hz, 2H, H^{C2}), 7.97 (ddd, *J* = 7.3, 5.5, 1.4 Hz, 2H, H^{A5}), 7.53 (d, *J* = 7.9 Hz, 2H, H^{C3}), 2.45 (s, 3H, -CH_3). 2.08 (s, 3H, NCH_3) ppm.
^{13}C{^{1}H} NMR (400 MHz, DMSO) δ 157.3 (C^{A2}), 155.3 (C^{B2}), 153.8 (C^{B4}), 150.5 (C^{A6}), 143.1 (C^{A4}), 142.4 (C^{C4}), 131.5 (C^{C1}), 130.1 (C^{C3}), 128.9 (C^{A5}), 128.0 (C^{C2}), 125.7 (C^{A3}), 121.1 (C^{B3}), 118.1 (CH_3*C*N), 21.0 (Ar-*C*H_3), 1.1 (*C*H_3CN) ppm.
HR-ESI-MS *m/z* 466.0129 [M+Cl]$^+$ requires 466.0145. (M = [Pd(ttpy)]$^{2+}$)

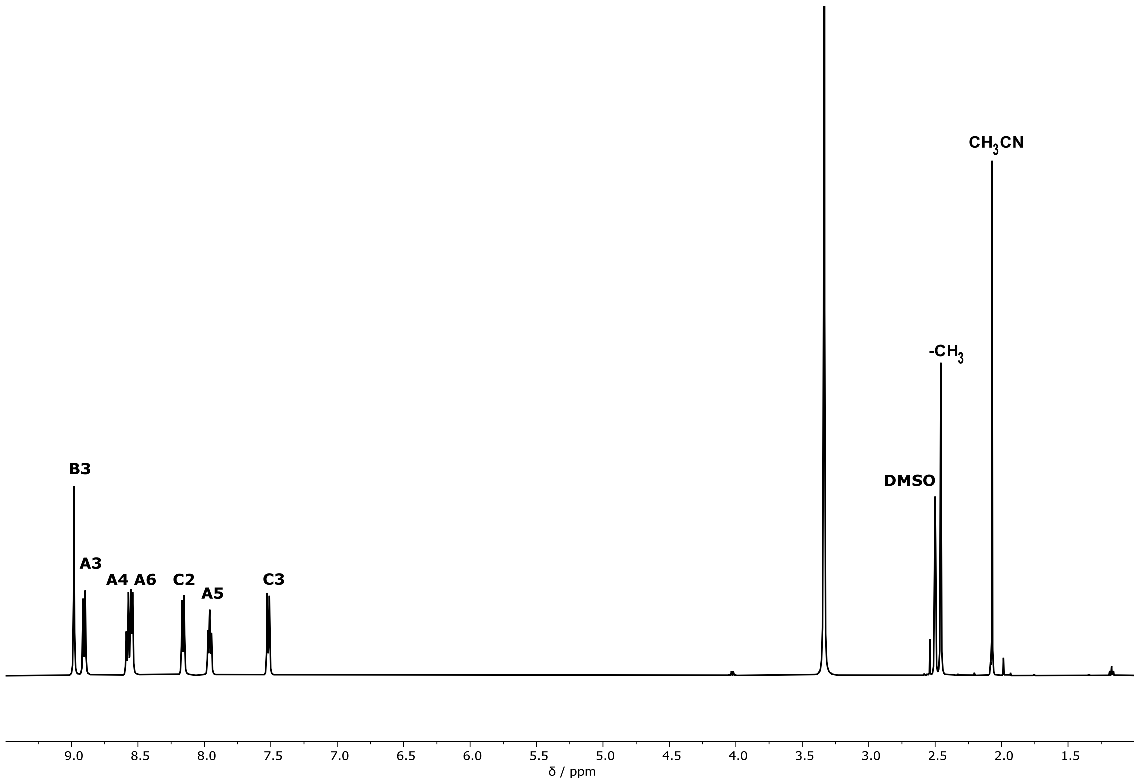

Figure S19 ^{1}H NMR spectrum (500 MHz, DMSO-*d*$_6$, 298 K) of [Pd(ttpy)(MeCN)]$(BF_4)_2$.

3.3.1. 2D NMR spectra of [Pd(ttpy)(MeCN)]$(BF_4)_2$

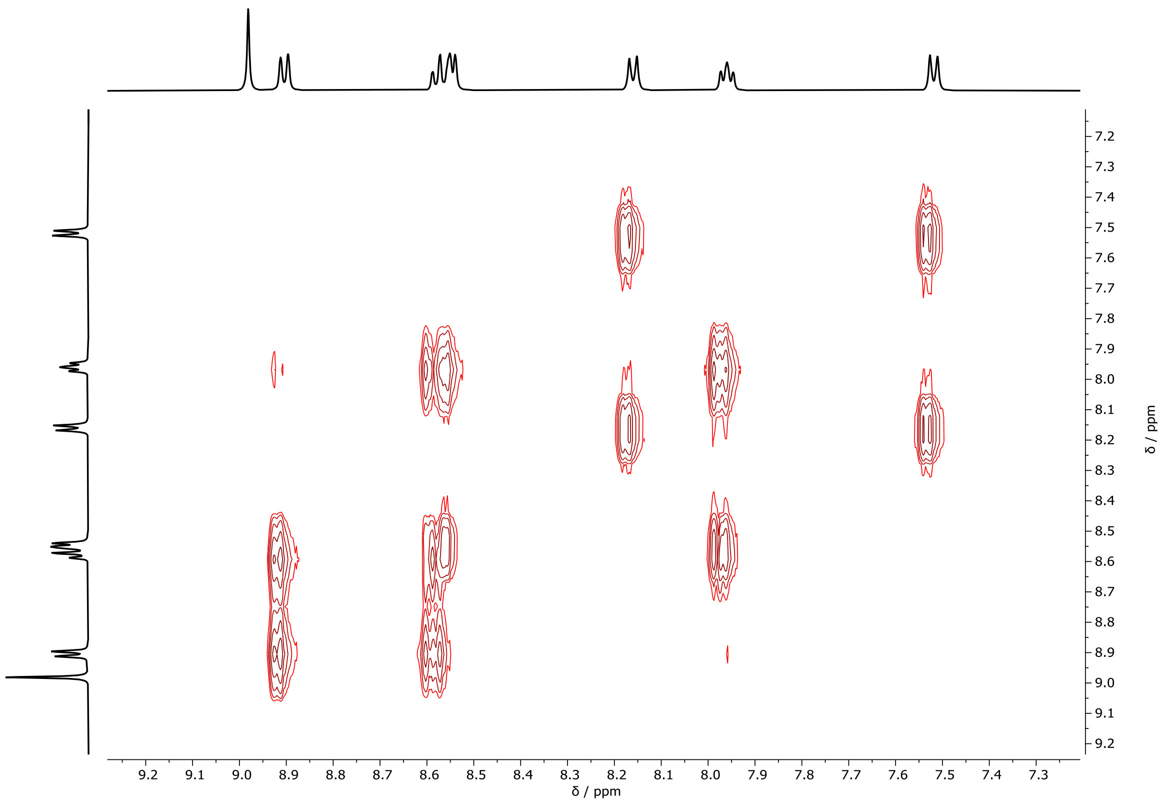

Figure S20 ^{1}H COSY NMR spectrum (500 MHz, DMSO-*d*$_6$, 298 K) of [Pd(ttpy)(MeCN)]$(BF_4)_2$.

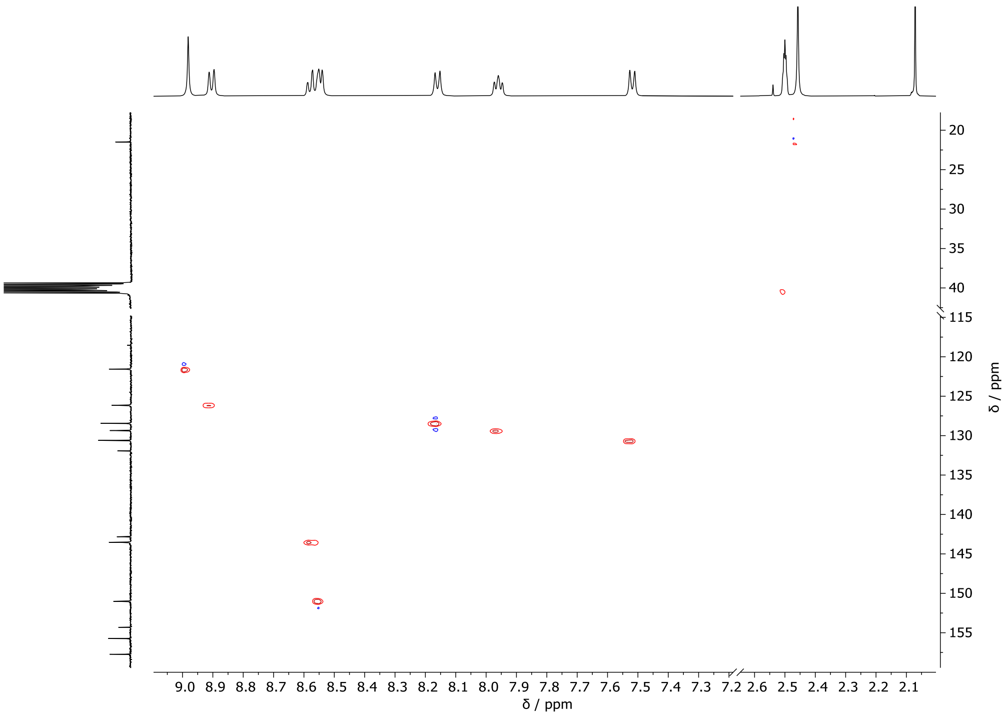

Figure S21 ^{1}H-^{13}C HSQC NMR spectrum (500 MHz, DMSO-d_6, 298 K) of [Pd(ttpy)(MeCN)](BF_4)$_2$.

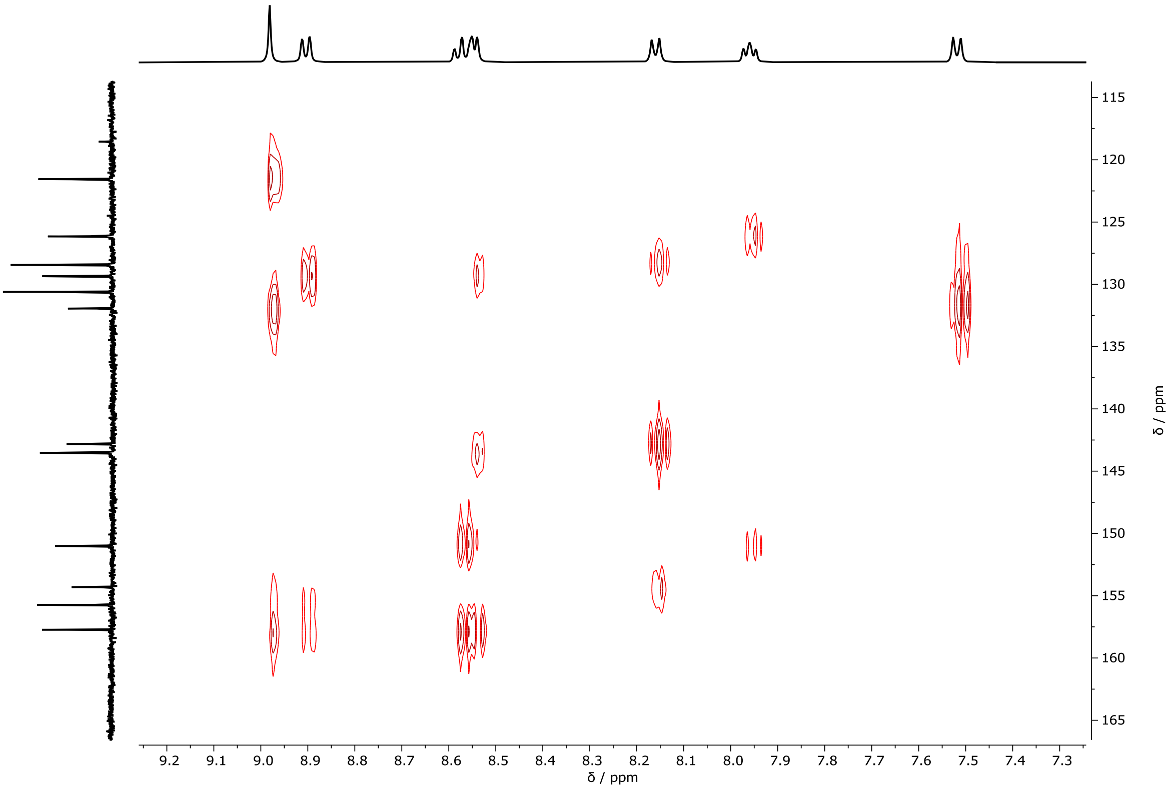

Figure S22 ^{1}H-^{13}C HMBC NMR spectrum (500 MHz, DMSO-d_6, 298 K) of [Pd(ttpy)(MeCN)](BF_4)$_2$.

3.4. Synthesis of [Pd(ttpy)(*E*-3)]$(BF_4)_2$

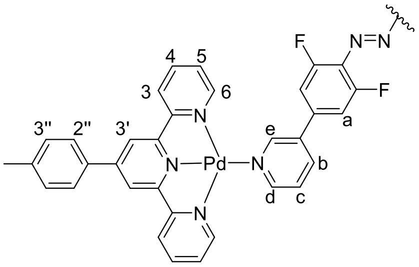

A solution of [Pd(ttpy)(CH_3CN)]$(BF_4)_2$ (300 µL, 12.8 mM, 3.84 µmol) in DMSO-d_6 and a solution of *E*-**3** (300 µL, 25.5 mM, 76.5 µmol) in DMSO-d_6 were combined in an NMR tube. The sample was heated at 40 °C for 20 min before being monitored by ^{1}H NMR.

3.5. 2D NMR spectra of [Pd(ttpy)(*E*-3)]$(BF_4)_2$

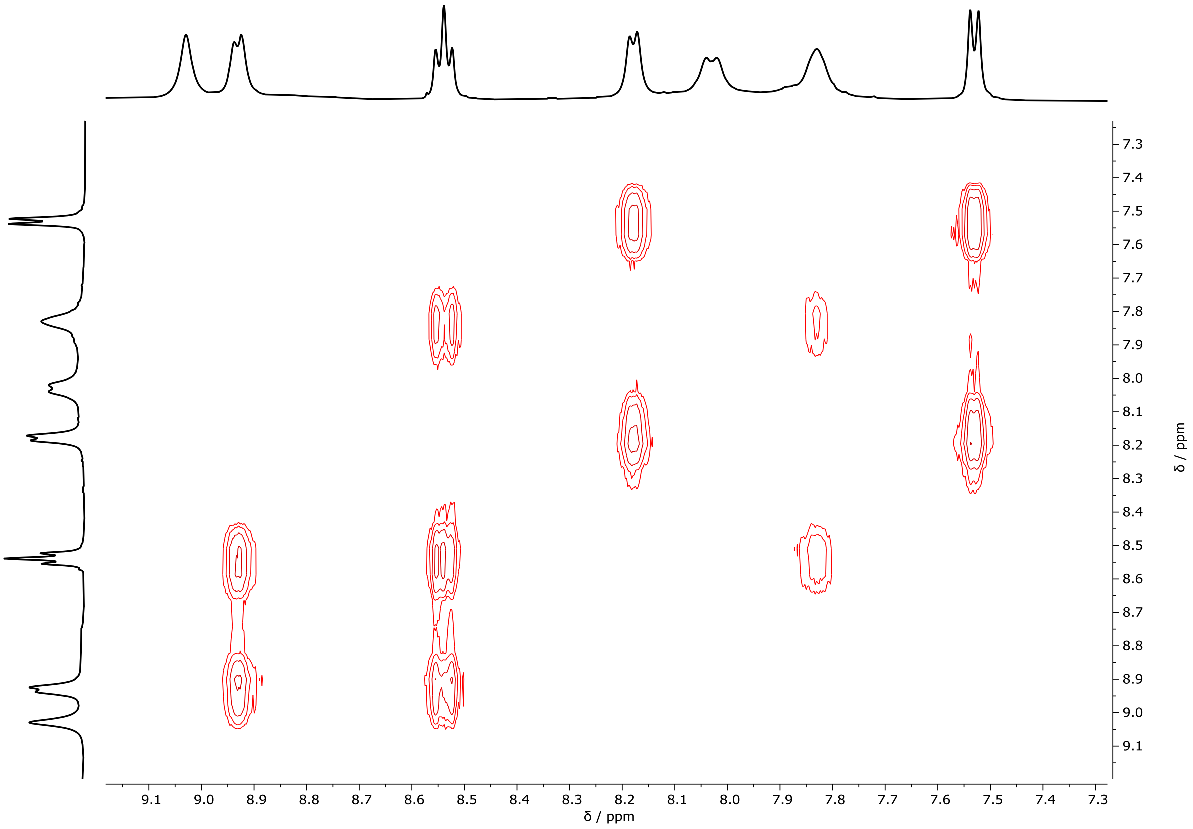

Figure S23 ^{1}H COSY NMR spectrum (500 MHz, DMSO-d_6, 298 K) of [Pd(ttpy)(*E*-**3**)]$(BF_4)_2$.

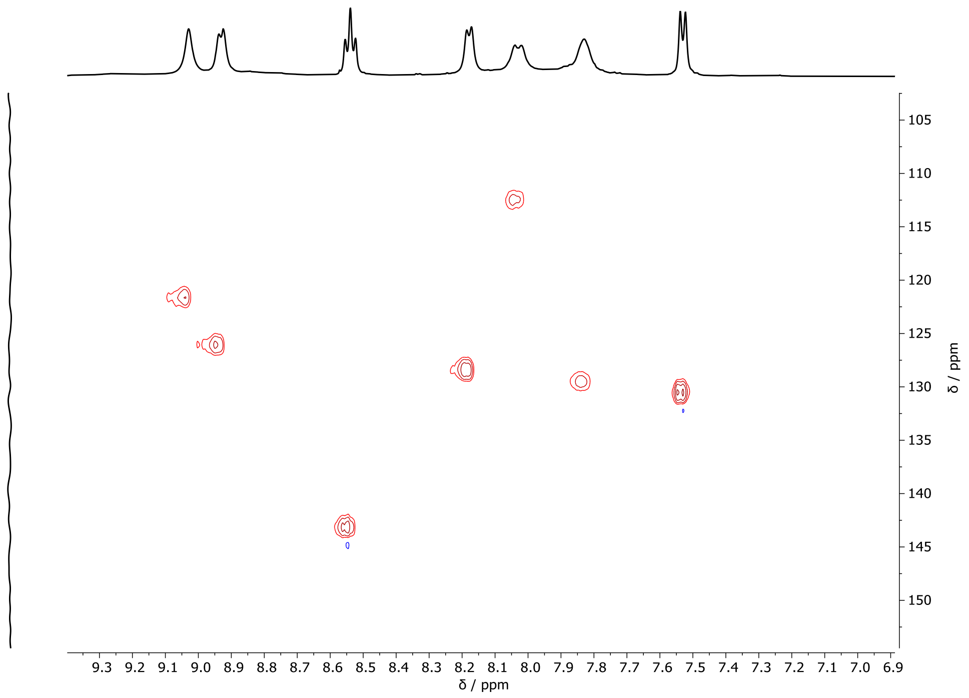

Figure S24 ^{1}H-^{13}C HSQC NMR spectrum (500 MHz, DMSO-d_6, 298 K) of [Pd(ttpy)(*E*-**3**)](BF_4)$_2$. A ^{13}C NMR spectrum of the product could not be collected due to the low concentration of the sample.

3.6. Synthesis of [Pd(ttpy)(3-methylpyridine)]$(BF_4)_2$

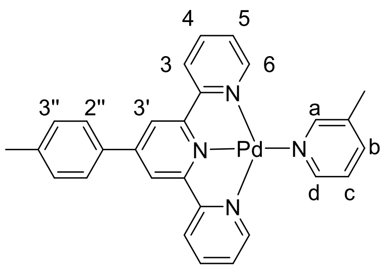

A solution of 3-methylpyridine (115 µL, 822 mM, 94.5 µmol) in DMSO-*d₆* was added to a solution of [Pd(ttpy)(CH_3CN)]$(BF_4)_2$ (600 µL, 16.0 mM, 9.57 µmol). The sample was monitored by ^{1}H NMR spectroscopy.

^{1}H NMR (500 MHz, DMSO) δ 9.07 (s, 2H), 9.01 – 8.93 (m, 2H), 8.55 (td, *J* = 7.9, 1.5 Hz, 2H), 8.41 (s, 7H), 8.37 (s, 7H), 8.24 – 8.17 (m, 2H), 7.81 (ddd, *J* = 7.5, 5.6, 1.3 Hz, 2H), 7.71 (dd, *J* = 5.6, 1.5 Hz, 2H), 7.63 – 7.57 (m, 7H), 7.56 – 7.50 (m, 2H), 7.27 (s, 4H), 2.47 (s, 3H), 2.29 (s, 25H), 2.07 (s, 2H) ppm.

3.7. 2D NMR spectra of [Pd(ttpy)(3-methylpyridine)]$(BF_4)_2$

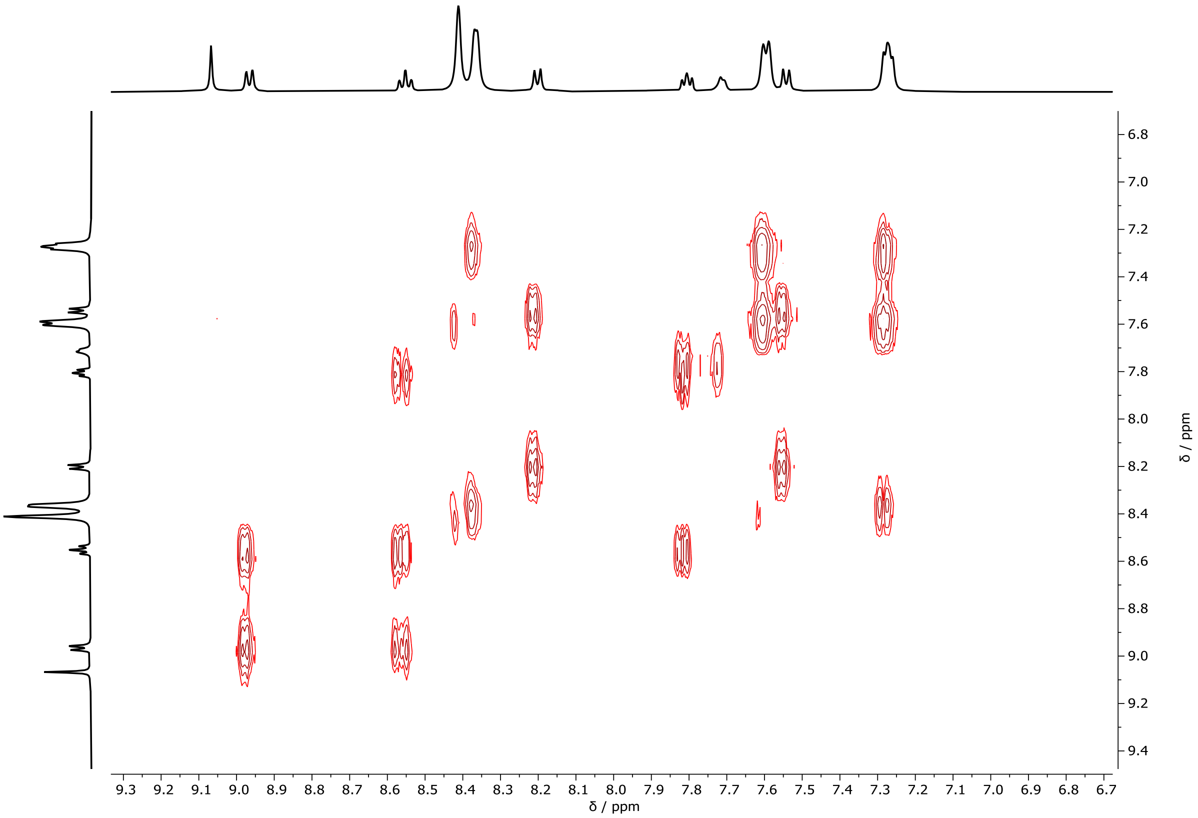

Figure S25 ^{1}H COSY NMR spectrum (500 MHz, DMSO-d_6, 298 K) of [Pd(ttpy)(3-methylpyridine)]$(BF_4)_2$.

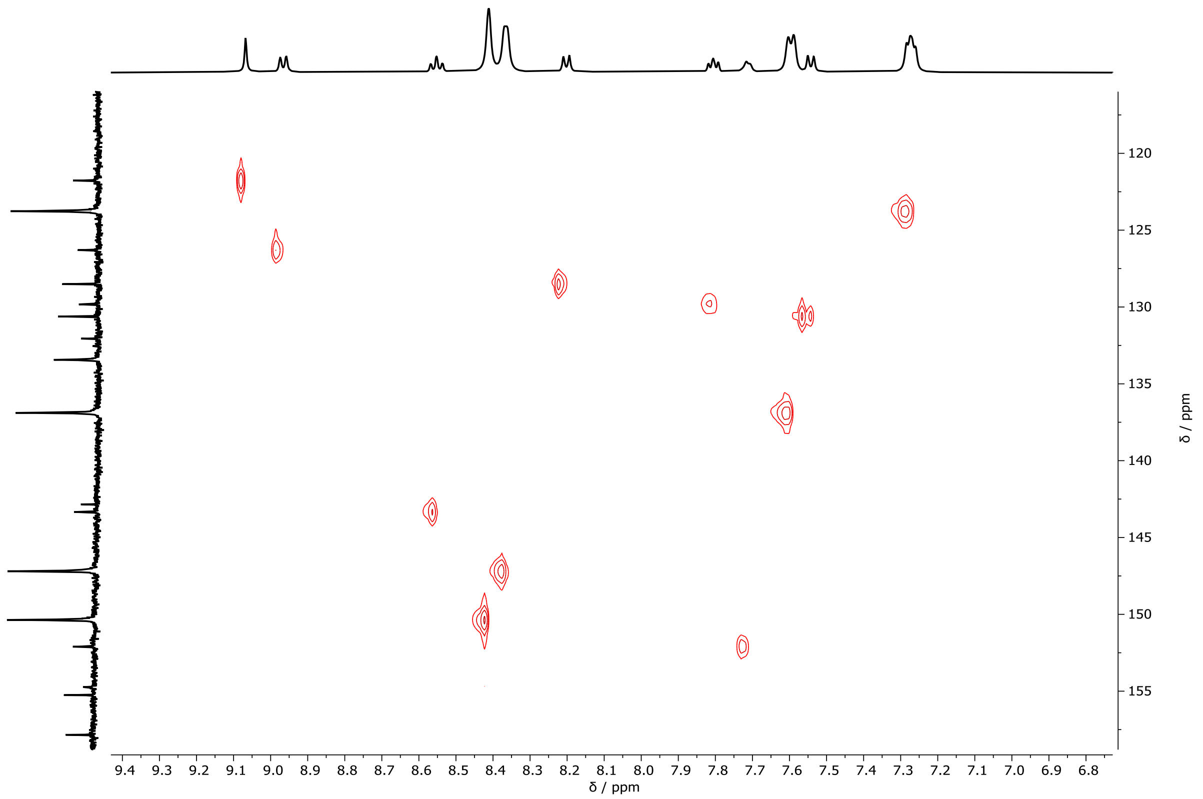

Figure S26 ^{1}H-^{13}C HSQC NMR spectrum (500 MHz, DMSO-d_6, 298 K) of [Pd(ttpy)(3-methylpyridine)]$(BF_4)_2$.

4. Photoswitching of ligand 3

4.1. ^{1}H and ^{19}F NMR spectra of photoswitching of 3

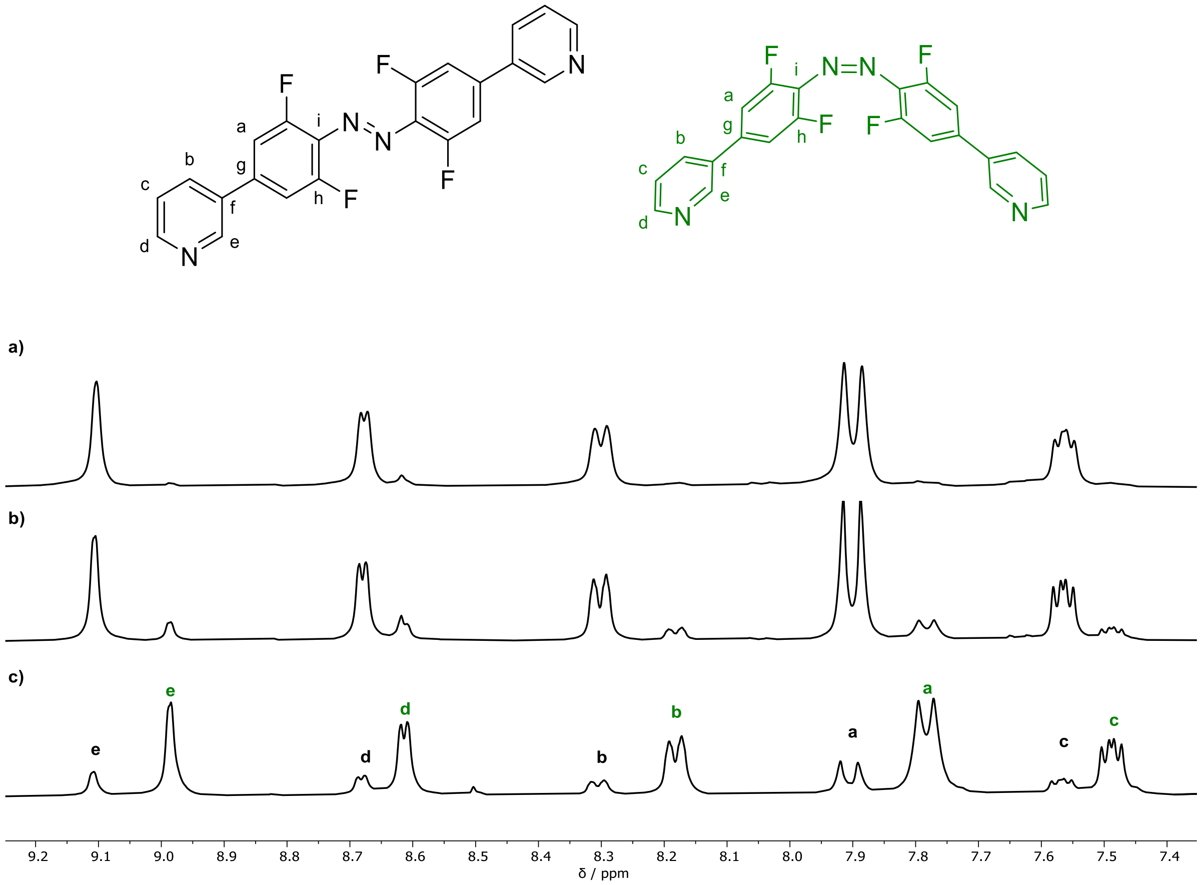

Figure S27 Photoswitching of **3** in DMSO-d_6 monitored with ^{1}H NMR (400 MHz, 298 K). a) Initial spectrum; b) after 15 minutes *ex situ* irradiation with a 410 nm LED (15% *Z*-**3**); c) after 85 minutes *ex situ* irradiation with a 530 nm LED (80% *Z*-**3**).

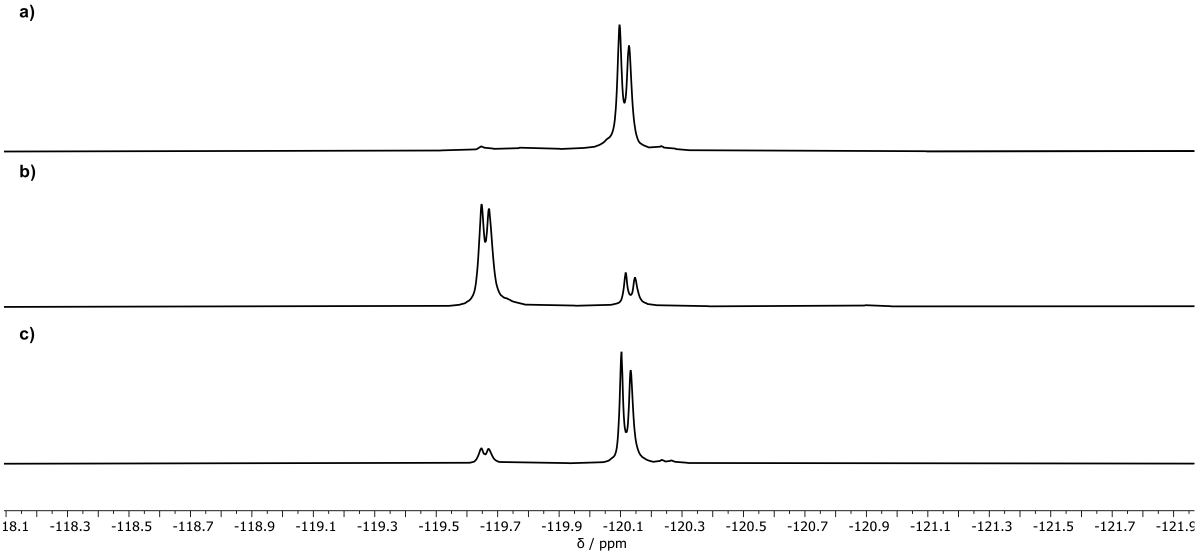

Figure S28 Photoswitching of **3** in DMSO-d_6 monitored with ^{19}F NMR (564.6 MHz, 298 K). a) Initial spectrum; b) after 85 minutes *ex situ* irradiation with a 530 nm LED (80% *Z*-**3**); c) after 15 minutes *ex situ* irradiation with a 410 nm LED (15% *Z*-**3**).

4.2. UV-visible absorption spectra of ligand 3

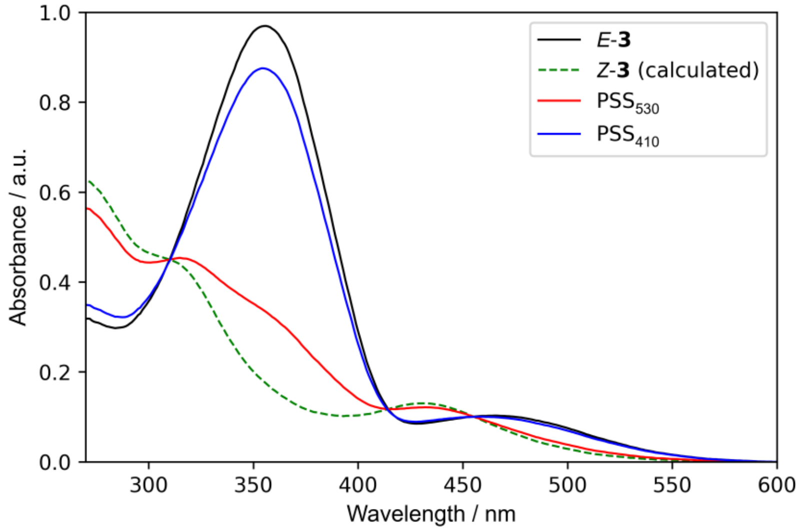

Figure S29 UV-visible absorbance spectra at 298 K in DMSO. Irradiation at 410 nm generates a PSS containing 15% *Z*-**3**. Irradiation at 530 nm generates a PSS containing 80% *Z*-**3**. Isomer ratios were calculated from ^{19}F NMR signal integrations. c) UV-Vis spectroscopy at 298 K in DMSO. The spectrum of *Z*-**3** was calculated using the isomer ratio determined by ^{19}F NMR signal integrations of a sample irradiated at 530 nm.

4.3. Determination of the thermal half-life of 3 by NMR spectroscopy

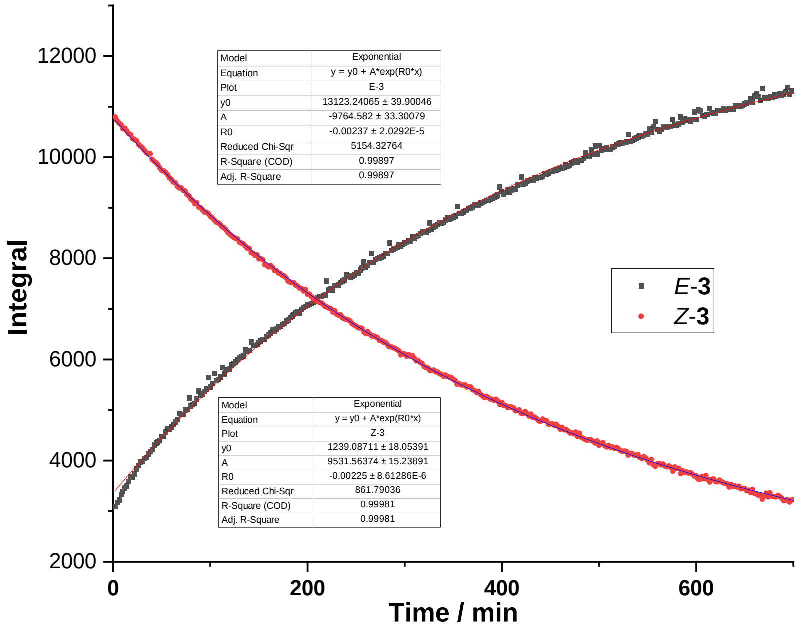

Figure S30 Thermal equilibration of *Z*-**3** after irradiation with 530 nm light. Sample was kept at 333 K during the measurement and the data was fit to a mono-exponential curve to give a thermal half-life of ~5 hour (~25 days at rt).

4.4. The effect of palladium(II) on the switching properties of ligand 3

To test whether binding to palladium(II) affects the photoswitching of **3**, [Pd(ttpy)(MeCN)]$(BF_4)_2$ was used as a source of palladium to prevent the palladium center bridging between multiple ligands and therefore prevent the formation of larger structures. Combining **3** and [Pd(ttpy)(MeCN)]$(BF_4)_2$ in DMSO-d_6 results in a moderate downfield shift of the of the NMR signals of **3**, and considerable lineshape broadening. The broadening of signals is attributed to the rate of exchange of **3** on [Pd(ttpy)(MeCN)]$(BF_4)_2$ occurring at a rate close to the NMR timescale.

While the ^{1}H NMR signals for *E*-**3** become broad when [Pd(ttpy)(MeCN)]$(BF_4)_2$ is added, the same effect is not observed when $[Pd(MeCN)_4](BF_4)_2$ is added. Instead, well-resolved signals corresponding to the two self-assembled products are observed. This contrast indicates that the rate at which *E*-**3** binds and dissociates to palladium is significantly slower when *E*-**3** is a part of the self-assembled structures.

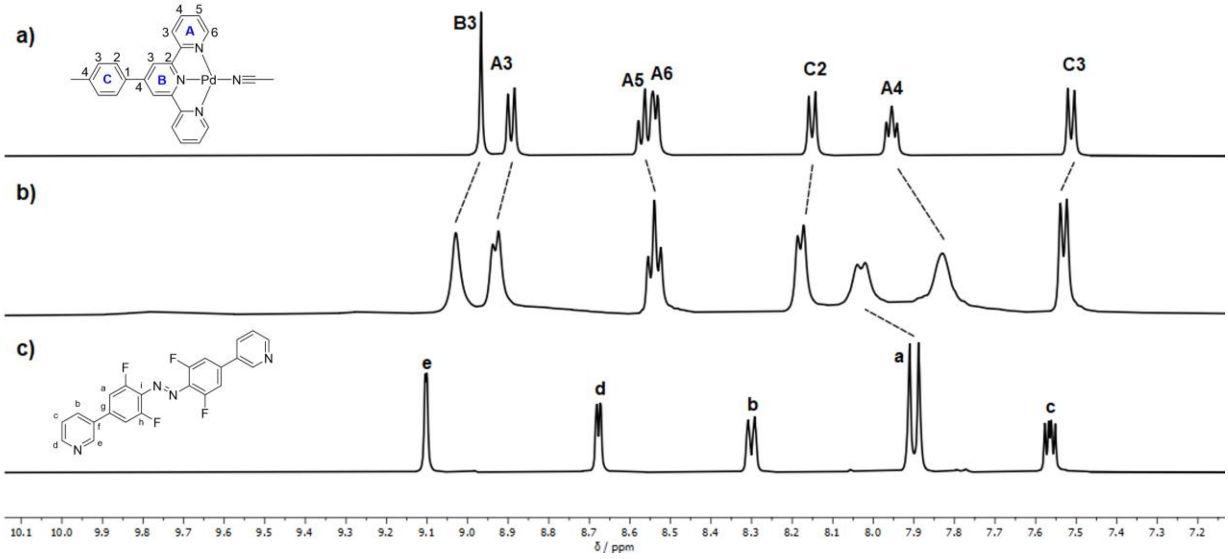

Figure S31 ^{1}H NMR spectra (500 MHz, DMSO-d_6, 298 K) of a) [Pd(ttpy)(MeCN)]$(BF_4)_2$, b) [Pd(ttpy)(MeCN)]$(BF_4)_2$ and **3** and c) *E*-**3**.

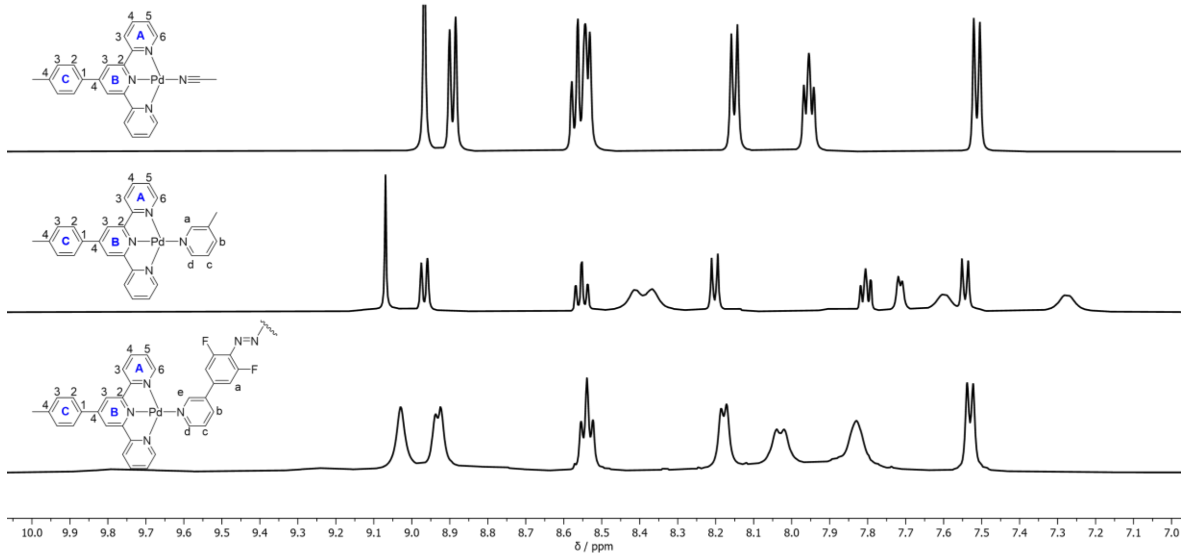

Figure S32 ^{1}H NMR spectra (500 MHz, DMSO-d_6, 298 K) of a) [Pd(ttpy)(MeCN)]$(BF_4)_2$, b) [Pd(ttpy)(MeCN)]$(BF_4)_2$ with 3-methylpyridine, and c) [Pd(ttpy)(MeCN)]$(BF_4)_2$ with *E*-**3**.

The mixture of [Pd(ttpy)(MeCN)]$(BF_4)_2$ and 3-methylpyridine resulted in broad signals corresponding to the 3-methyl pyridine. To resolve these signals a variable temperature NMR spectroscopy experiment was performed on a mixture of [Pd(ttpy)(MeCN)]$(BF_4)_2$ (5.6 µmol, 500 µL, 11 mM) with 5 added equivalents of 3-methylpyridine (Figure S33). NMR spectra of the mixture were collected at 298 K, 313 K, 328 K and 343 K. Increasing the temperature did not resolved the broad signals.

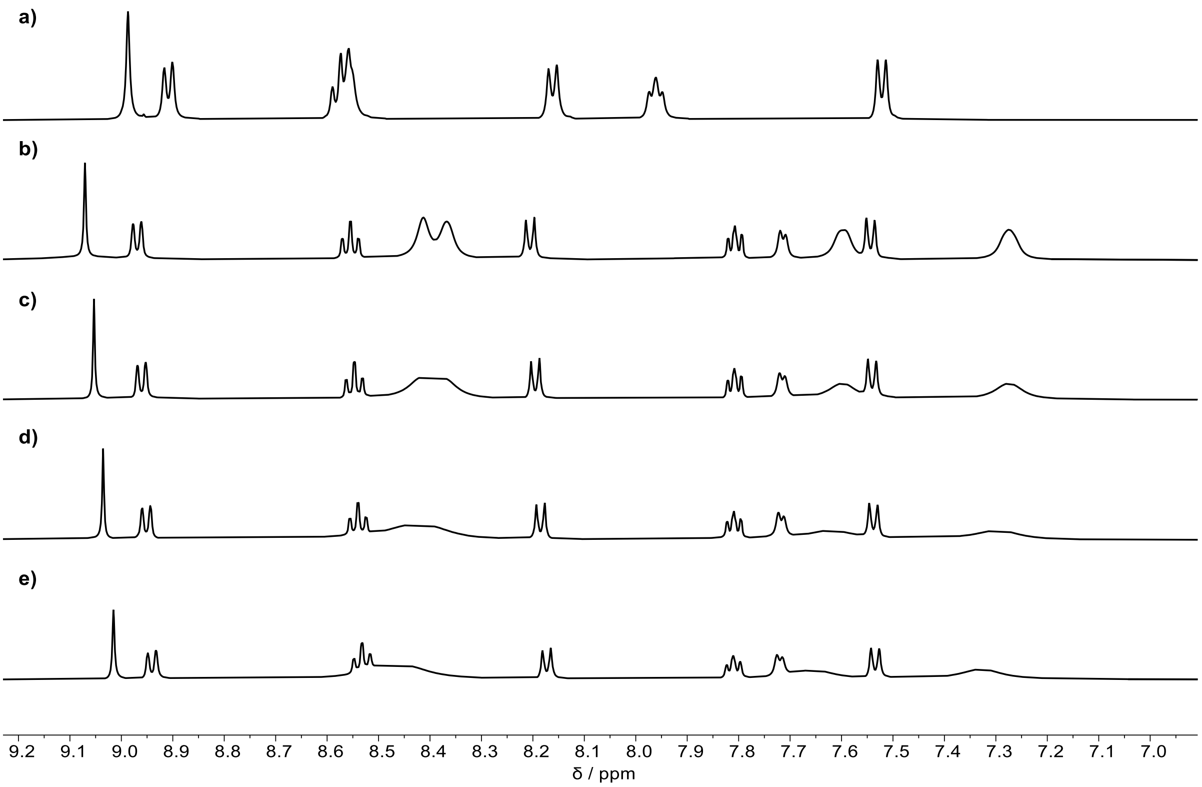

Figure S33 ^{1}H NMR spectra (500 MHz, DMSO-*d*$_6$) of a) [Pd(ttpy)(MeCN)]$(BF_4)_2$ at 298 K, b) [Pd(ttpy)(MeCN)]$(BF_4)_2$ + 5 equivalents of 3-methylpyridine at 298 K, c) [Pd(ttpy)(MeCN)]$(BF_4)_2$ + 5 equivalents of 3-methylpyridine at 313 K, d) [Pd(ttpy)(MeCN)]$(BF_4)_2$ + 5 equivalents of 3-methylpyridine at 328 K, and e) [Pd(ttpy)(MeCN)]$(BF_4)_2$ + 5 equivalents of 3-methylpyridine at 343 K.

To test if the thermal isomerization of *Z*-**3** to *E*-**3** is affected by binding to palladium(II), the thermal half-life of *Z*-**3** was measured in the presence of [Pd(ttpy)(DMSO)]$(BF_4)_2$. The palladium(II) complex was prepared by repeated cycles of dissolving in DMSO and removing the solvent under reduced pressure and heating to remove the acetonitrile. A sample of **3** (1 equiv., 0.13 µmol, 50 µM) was enriched in *Z*-**3** was by irradiating with 530 nm light. [Pd(ttpy)(DMSO)]$(BF_4)_2$ (100 equivalent, 13 µmol, 5.1 mM) was added. The sample was left in the dark at 333K over 700 min and the absorbance at 500 nm was monitored. The absorbance was plotted against time and fit to a mono-exponential curve to calculate the rate constant. The rate constant was used to calculate a thermal half-life of 354 minutes at 333K and a corresponding apparent thermal barrier of 110 kJ·mol^{-1}. The calculated apparent thermal barrier of isomerization was the same with and without the added palladium (Figure S34). These results suggest that the thermal isomerization of ligand *Z*-**3** is not affected by palladium at these low concentrations.

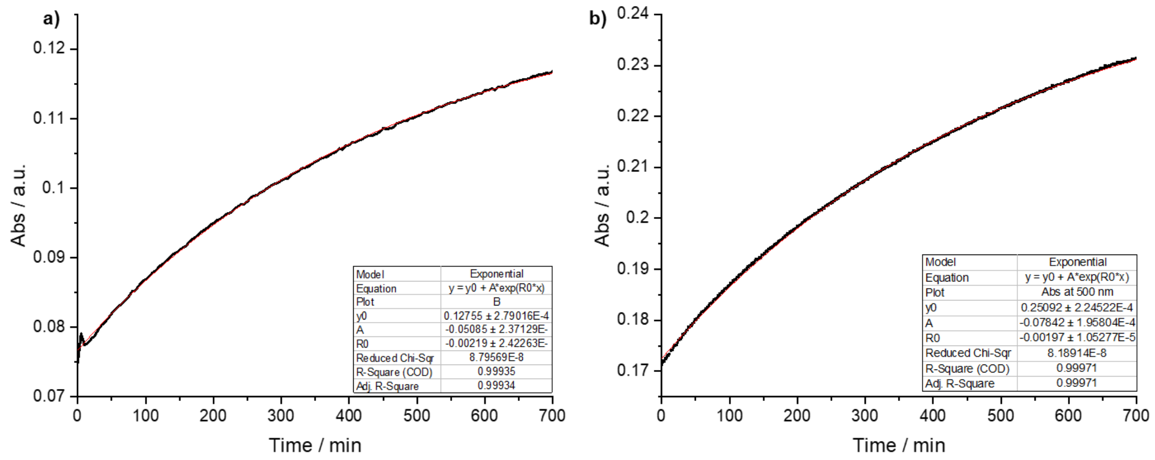

Figure S34 Thermal isomerization of a) *Z*-**3** (1 equivalent, 0.13 µmol, 50 µM) to *E*-**3** and b) *Z*-**3** to *E*-**3** with the addition of [Pd(ttpy)(DMSO)](BF_4)$_2$ (100 equivalent, 13 µmol, 5.1 mM) after irradiation with 530 nm light in DMSO. The absorbance was monitored at 500 nm and the samples were kept at 333 K in the dark during the measurements. Data were fit to mono-exponential curves to calculate rate constant.

Table S2 Summary of kinetic data for thermal isomerisation of *Z*-**3**.

Equivalents of Pd(ttpy)(MeCN)(BF_4)$_2$ added	k / ×10^{-5} s^{-1}	$τ_{1/2}$ / min	Apparent barrier / kJmol^{-1}
0	3.65	317	110
100	3.28	354	110

5. Photoswitching of compound 4

5.1. ^{1}H and ^{19}F NMR spectra of photoswitching of 4

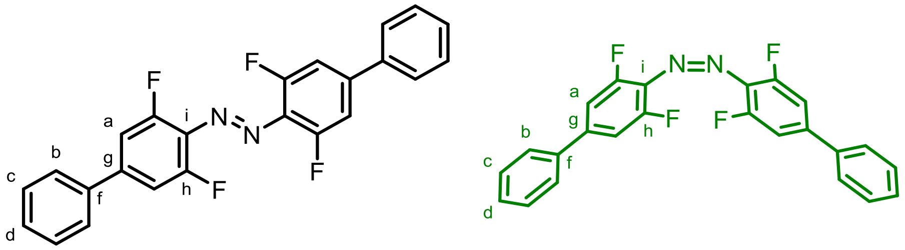

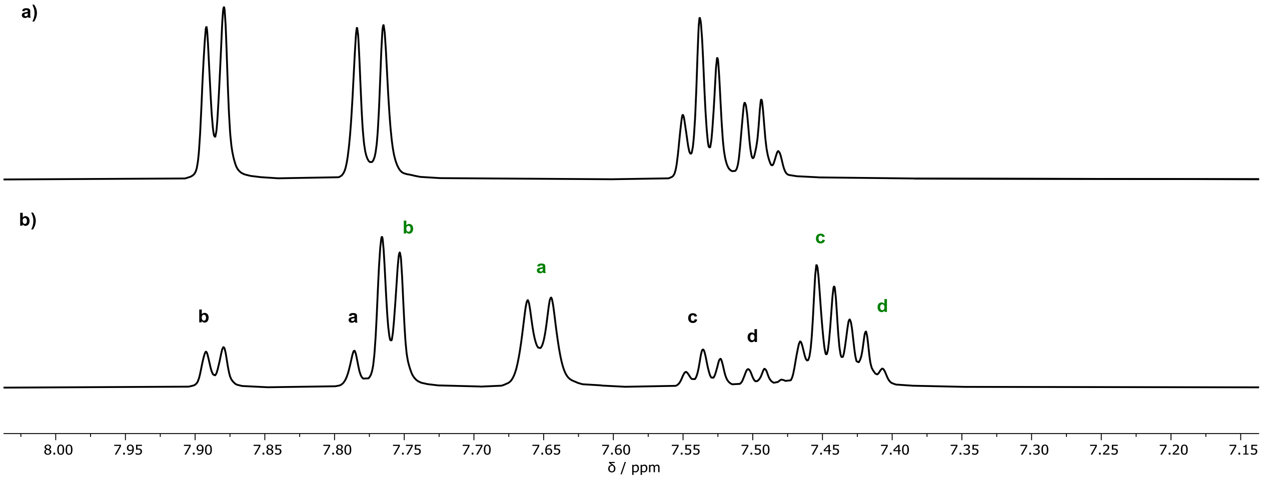

Figure S35 Photoswitching of **4** in DMSO-d_6 monitored with ^{1}H NMR (600 MHz, 298 K). a) Initial spectrum; b) after 65 minutes *ex situ* irradiation with a 530 nm LED (78% *Z*-**4**).

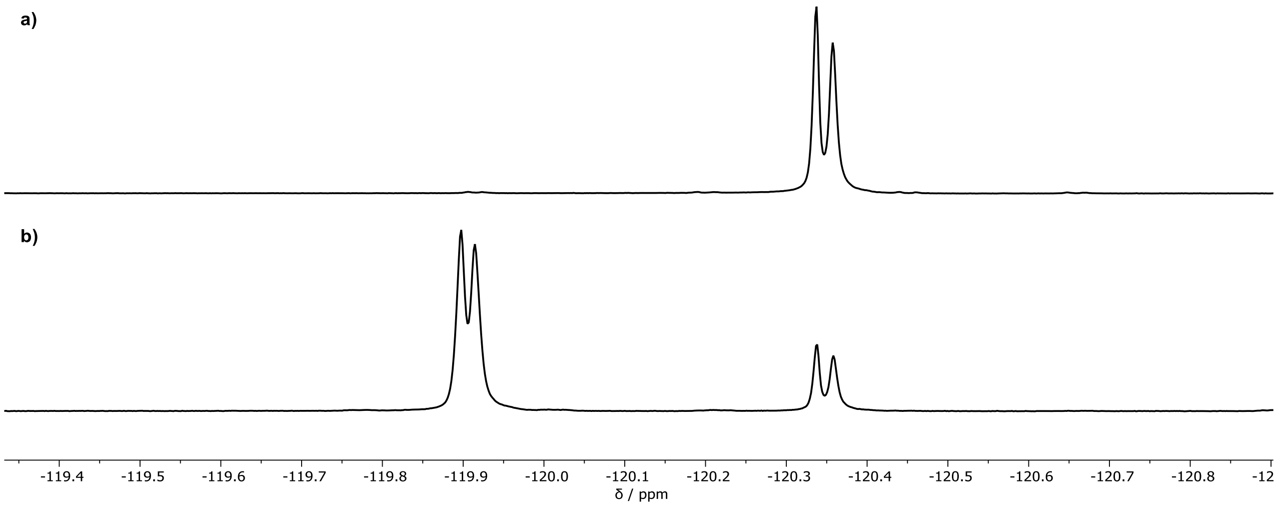

Figure S36 Photoswitching of **4** in DMSO-d_6 monitored with ^{19}F NMR (564.6 MHz, 298 K). a) Initial spectrum; b) after 65 minutes *ex situ* irradiation with a 530 nm LED (78% *Z*-**4**).

5.2. UV-visible absorption spectra of ligand 4

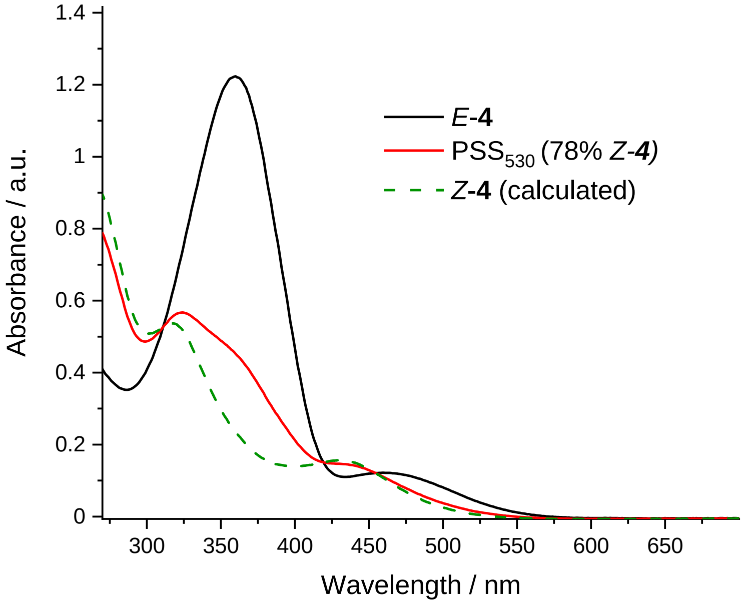

Figure S37 UV-visible absorbance spectra of compound **4** at 298 K in DMSO. Irradiation at 530 nm generates a PSS containing 78% *Z*-**4**. Isomer ratios were calculated from ^{19}F NMR signal integrations. c) UV-Vis spectroscopy at 298 K in DMSO. The spectrum of *Z*-**4** was calculated using the isomer ratio determined by ^{19}F NMR signal integrations of a sample irradiated at 530 nm.

5.3. Determination of the thermal half-life of 4 by UV-vis spectroscopy

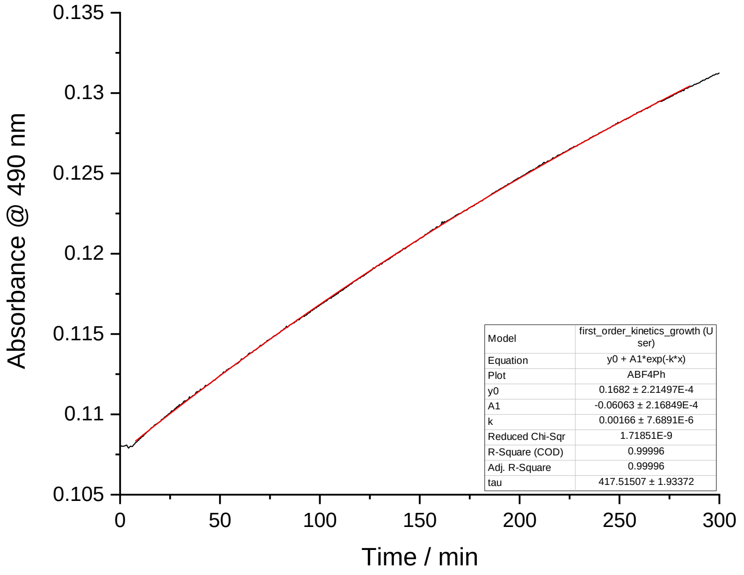

Figure S38 Thermal isomerization of **4** monitored by UV-vis absorption spectroscopy at 490 nm (333 K, DMSO, 22 µM).

5.4. The effect of palladium(II) on the switching properties of 4

To test whether palladium(II) ions have an effect on the distribution of *ortho*-fluoroazobenzene isomers in solution, $[Pd(CH_3CN)_4](BF_4)_2$ (2.4 mg, 5.5 µmol, 11 mM) was added to a thermally equilibrated solution containing *E*-**4** (4.4 mg, 11 µmol, 22 mM) and a solution containing 78% *Z*-**4** (generated by irradiation at 530 nm for 65 minutes). Both the ^{1}H and ^{19}F NMR spectra show no additional peaks over the course of 24 h, with the only change being ascribed to thermal isomerization of *Z*-**4** to *E*-**4** (Figure S39 and Figure S40).

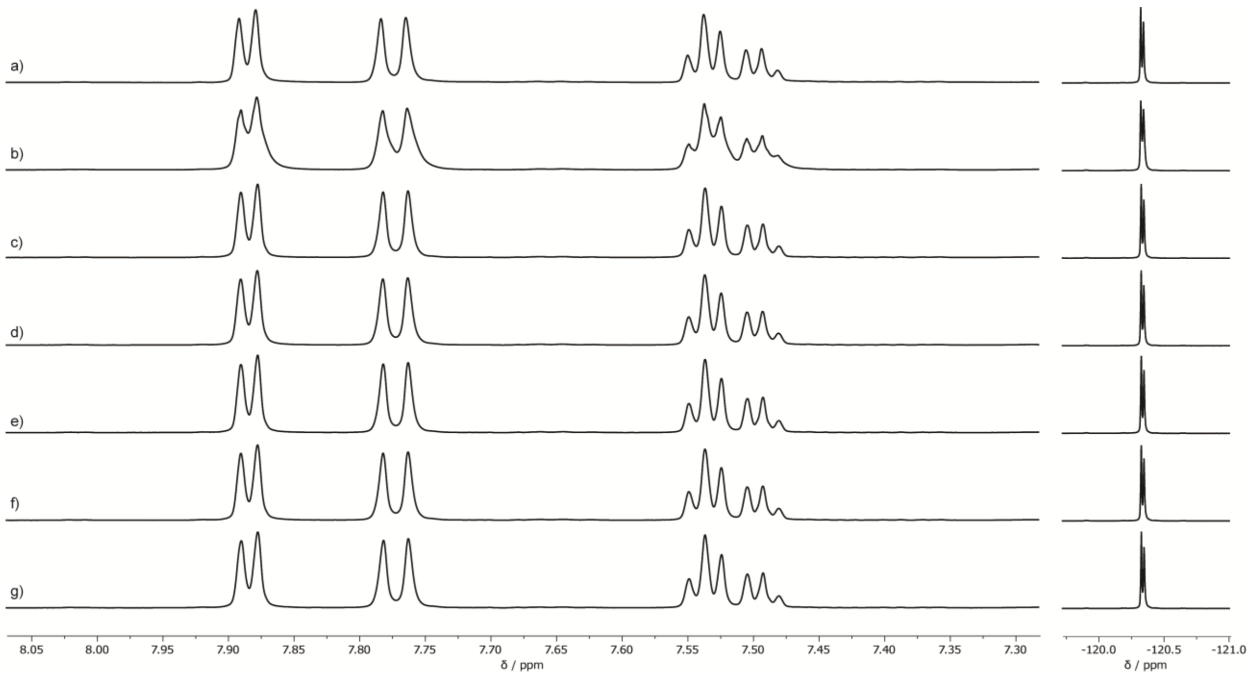

Figure S39 ^{1}H (600 MHz) and ^{19}F (564.6 MHz) NMR comparison of a) *E*-**4** b) after addition of 0.5 equivalents of $[Pd(CH_3CN)_4](BF_4)_2$; c) left in dark for 45 minutes; d) 2 h; e) 5 h; f) 6 h and g) 23 h.

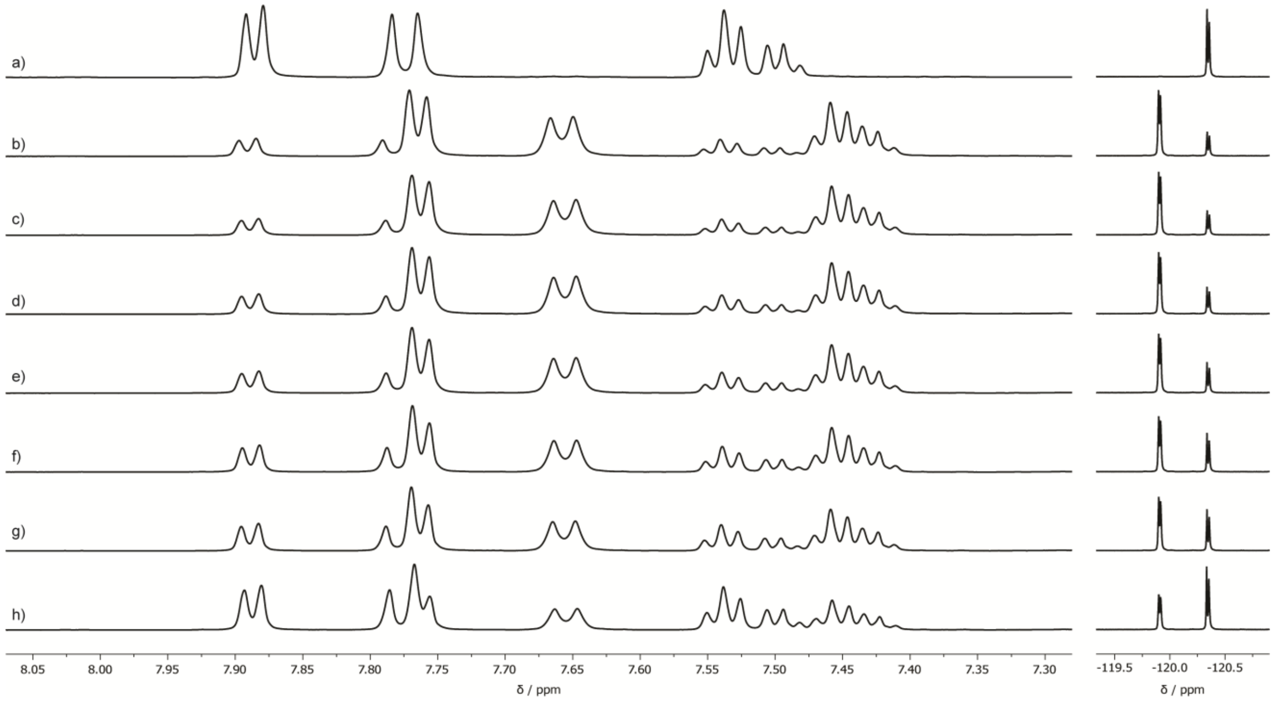

Figure S40 ^{1}H (600 MHz) and ^{19}F (564.6 MHz) NMR comparison of a) *E*-**4**; b) a mixture of *Z*-**4** (78%) and *E*-**4** (22%) generated by irradiation at 530 nm for 65 minutes; c) after addition of 0.5 equivalents of $[Pd(CH_3CN)_4](BF_4)_2$; c) left in dark for 45 minutes; d) 2 h; e) 5 h; f) 6 h and g) 23 h.

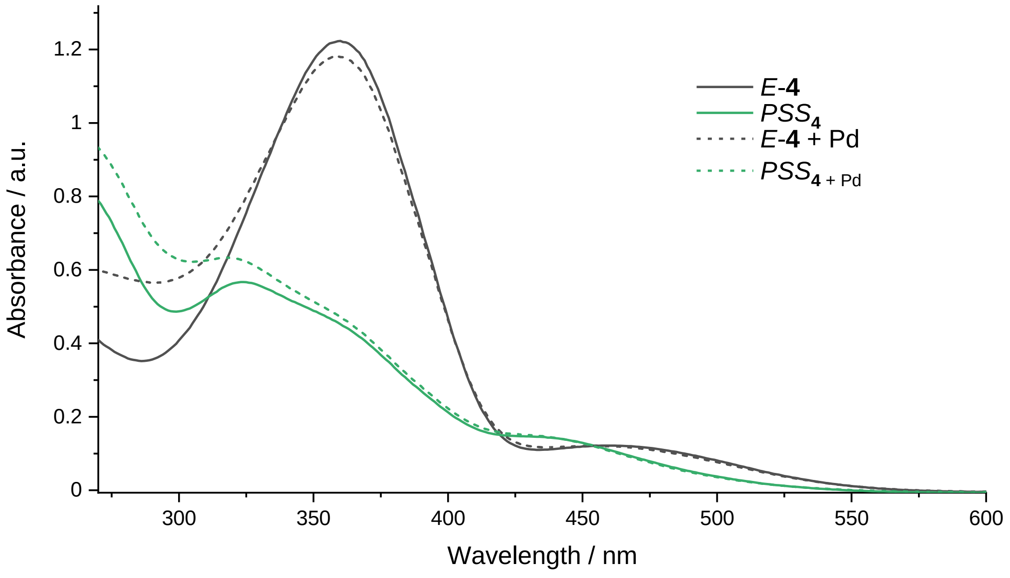

Figure S41 Photoswitching of **4** and **4** + $[Pd(CH_3CN)_4](BF_4)_2$ monitored by UV-vis spectroscopy (333K, DMSO, 36 µM). PSS**4** refers to the state containing 78% *Z*-**4** upon irradiation with 530 nm for 45 minutes. *E*-**4** + Pd refers to the mixture of **4** and 2 equivalents of $[Pd(CH_3CN)_4](BF_4)_2$ and PSS_{4+Pd} refers to the state generated upon irradiation at 530 nm for 45 minutes.

The thermal half-life of *Z*-**4** was measure using UV-vis absorption spectroscopy. A sample of *Z*-isomer enriched **4** was prepared by irradiating a sample of **4** (22 µM) with 530 nm light. The sample was left in the dark at 60 °C for 300 minutes and the absorbance at 490 nm was monitored. The absorbance was plotted against time and a mono-exponential curve was fit to the data to determine the rate constant used to calculate a half-life of 418 minutes (~7 hours) and apparent barrier of 111 kJ·mol^{-1} (Figure S42).
To test whether palladium(II) ions have an effect on the thermal isomerization of *Z*-**4**, the previous procedure was repeated with the addition of 0.5 equivalents of $[Pd(CH_3CN)_4](BF_4)_2$. This resulted in a half-life of 383 minutes and the same apparent barrier of 111 kJ·mol^{-1}, indicating that the presence of palladium(II) ions have no effect on the thermal isomerization of *Z*-**4**.

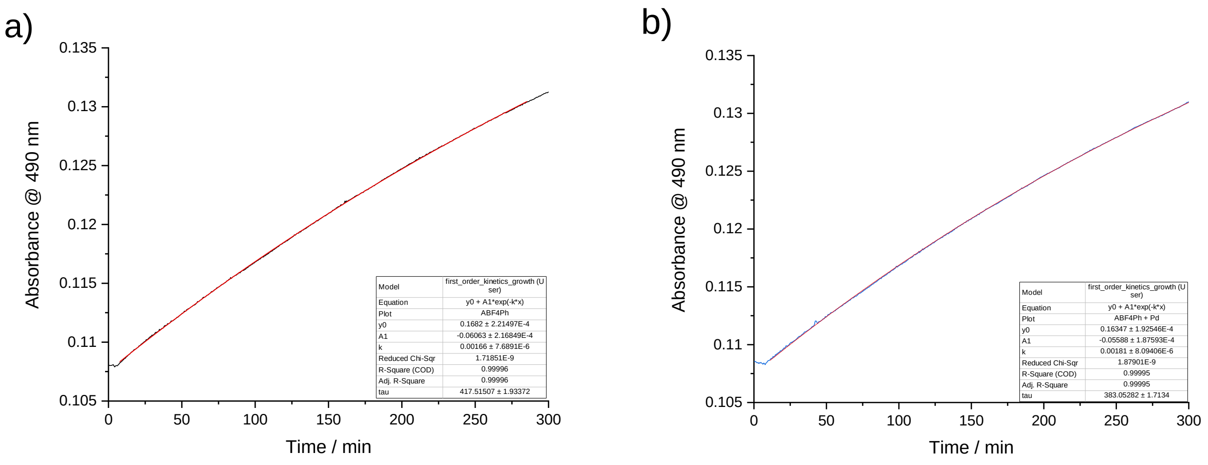

Figure S42 Thermal isomerization of a) **4** (see **Figure S38**) and b) **4** after addition of 0.5 equivalents of $[Pd(CH_3CN)_4](BF_4)_2$ monitored by UV-vis spectroscopy at 490 nm (333 K, DMSO, 22 µM).

To test whether palladium(II) affects the photoswitching properties of compound **4**, a thermally equilibrated solution of *E*-**4** was prepared (12 µmol, 500 µL, 24 mM). The sample was irradiated with a 410 nm LED until a PSS was reached (PSS_{410}), then a 530 nm LED until a PSS was reached (PSS_{530}). At each PSS, ^{1}H and ^{19}F NMR spectra were collected (Figure S43 and Figure S44) and the integrals of the ^{19}F NMR signals were used to determine the respective relative PSS distributions. This was repeated after adding a solution of $[Pd(MeCN)_4](BF_4)_2$ (1.8 equivalent, 22 µmol, 100 µL, 220 mM). Adding palladium(II) had no effect on the isomer distributions. The relative distribution of *E*-**4** at PSS_{410} was 82% before adding palladium(II) and 80% after, while at PSS_{530} the relative distribution of *E*-**4** was 18% before adding palladium(II) and 19% after adding palladium(II).

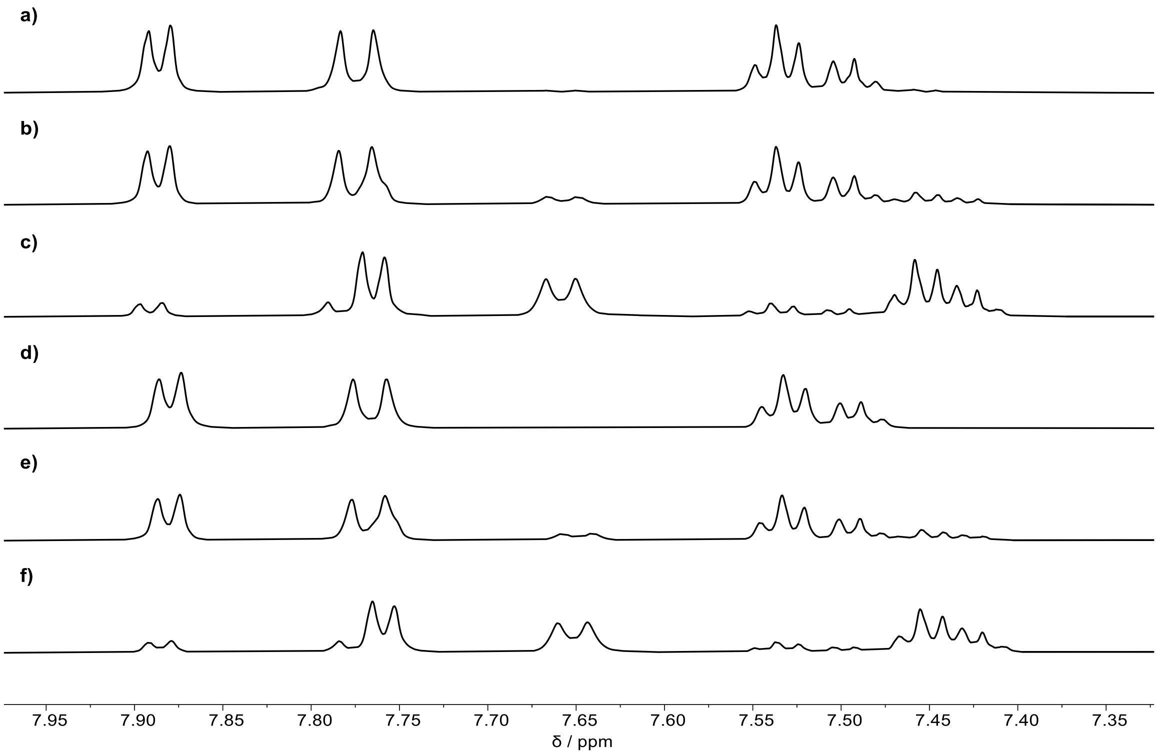

Figure S43 ^{1}H spectra (600 MHz, DMSO-d_6, 298 K) of a) compound *E*-**4** , b), compound **4** at PSS_{410} (82% *E*-**4**) c) compound **4** at PSS_{530} (18% *E*-**4**), d) compound *E*-**4** + 2 equivalents of $[Pd(MeCN)_4](BF_4)_2$, e) compound **4** + 2 equivalents of $[Pd(MeCN)_4](BF_4)_2$ at PSS_{410} (80% *E*-**4**), and f) compound **4** + 2 equivalents of $[Pd(MeCN)_4](BF_4)_2$ at PSS_{530} (19% *E*-**4**). Isomer distributions determined by integrating signals from ^{19}F NMR spectra.

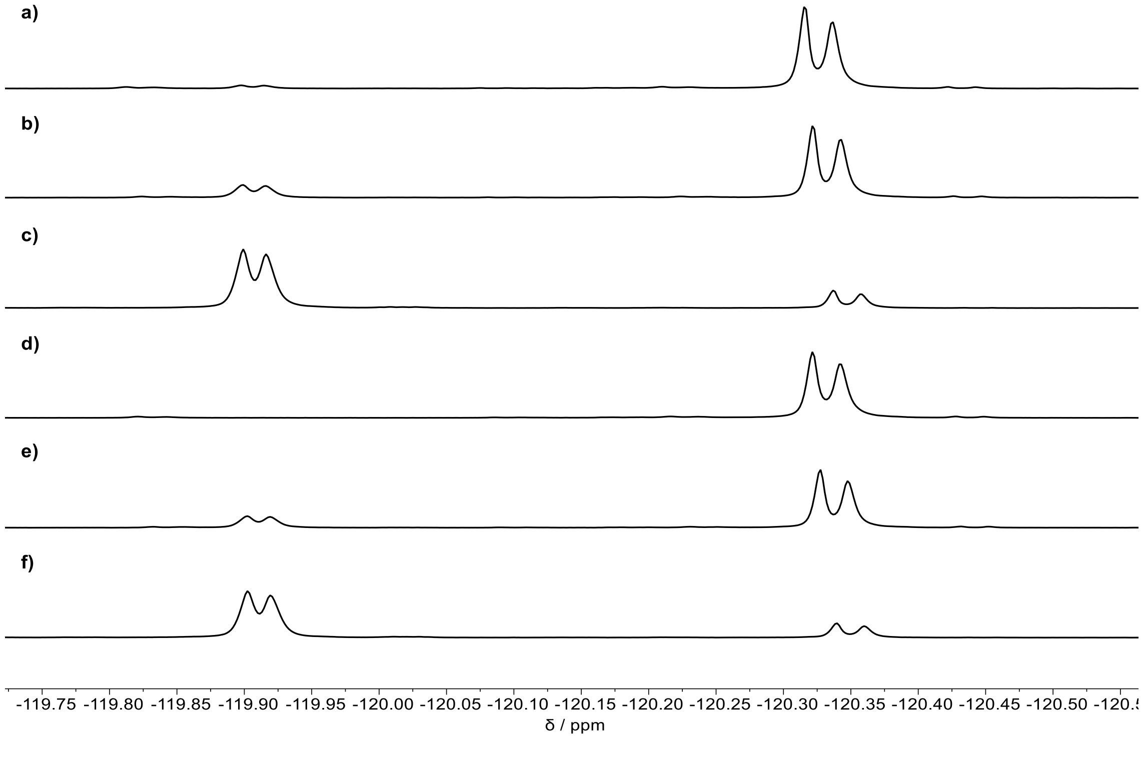

Figure S44 Partial ^{19}F spectra (564.6 MHz, DMSO-d_6, 298 K) of a) compound *E*-**4** , b), compound **4** at PSS_{410} (82% *E*-**4**) c) compound **4** at PSS_{530} (18% *E*-**4**), d) compound *E*-**4** + 2 equivalents of $[Pd(MeCN)_4](BF_4)_2$, e) compound **4** + 2 equivalents of $[Pd(MeCN)_4](BF_4)_2$ at PSS_{410} (80% *E*-**4**), and f) compound **4** + 2 equivalents of $[Pd(MeCN)_4](BF_4)_2$ at PSS_{530} (19% *E*-**4**). Isomer distributions determined by integrating signals from ^{19}F NMR spectra.

6. Formation of self-assembled products with ligand *E*-3

$[Pd(CH_3CN)_4](BF_4)_2$ (3.4 mg, 8 µmol) in DMSO-d_6 (25 µL) was added to *E*-**3** (6.2 mg, 15 µmol) dissolved in DMSO-d_6 (500 µL) in an NMR tube, leading to an immediate color change to deep red. The sample was equilibrated in the dark at room temperature and the reaction monitored by ^1H and ^{19}F NMR spectroscopy over 3 days.

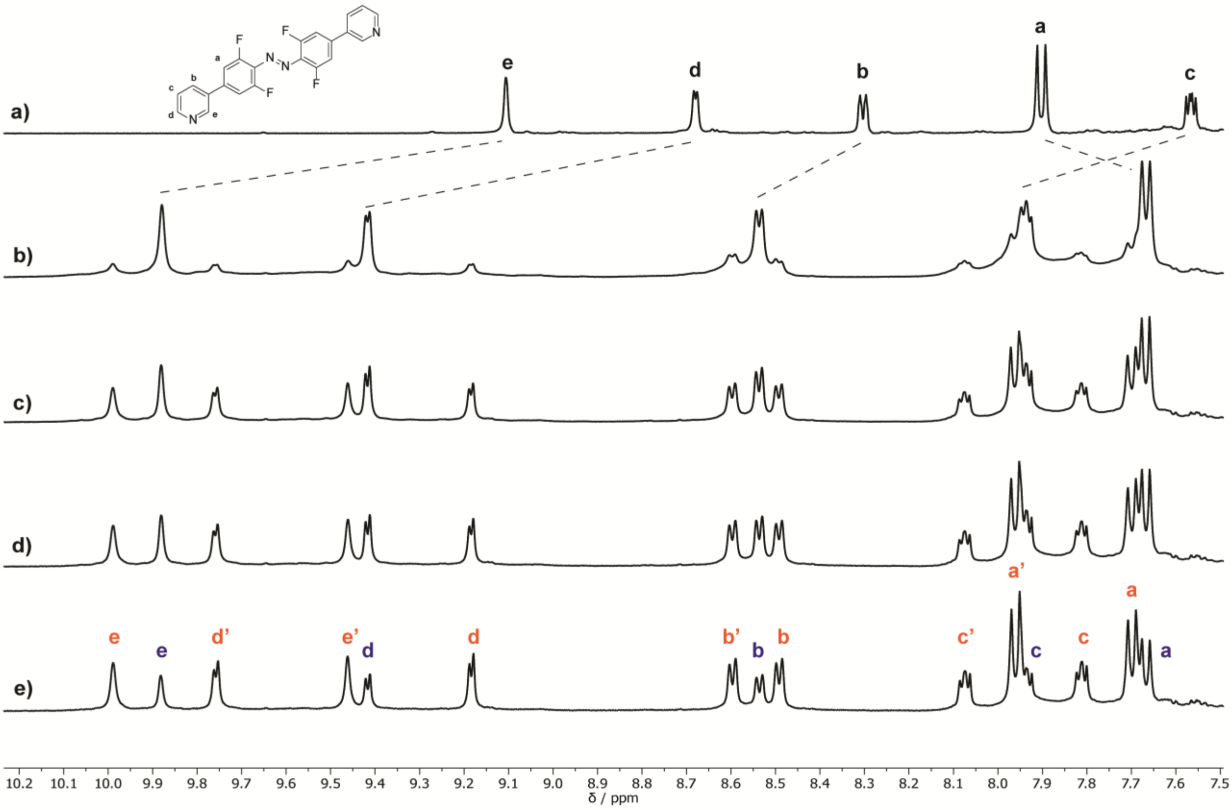

Figure S45 Reaction between *E*-**3** and $[Pd(CH_3CN)_4](BF_4)_2$ monitored by ^1H NMR (DMSO-d_6, 600 MHz, 298 K). a) *E*-**3** in DMSO-d_6; b) 5 minutes after addition of $[Pd(CH_3CN)_4](BF_4)_2$; c) after 190 minutes; d) 5 hours and e) 22 hours.

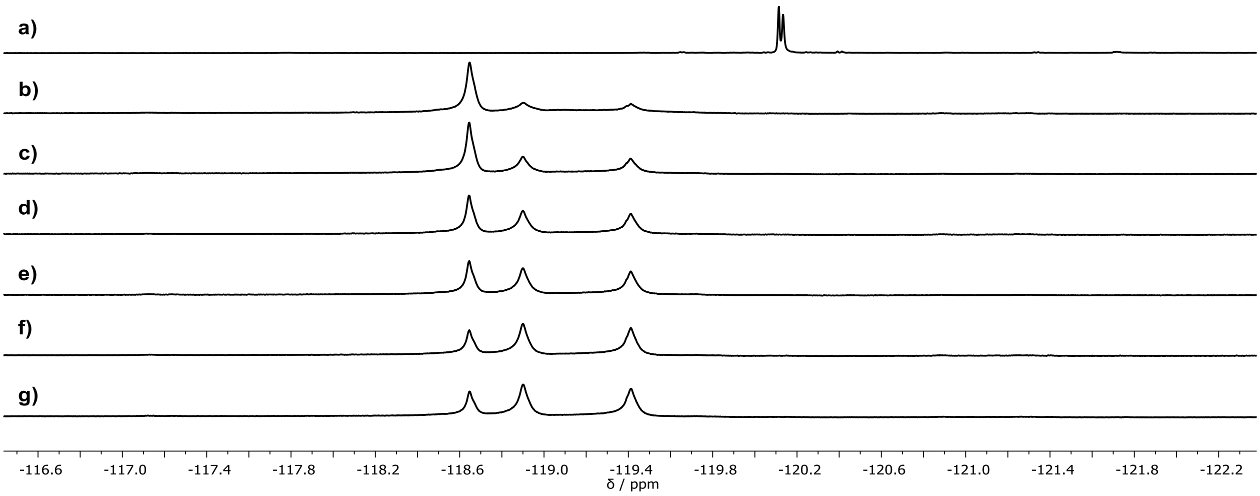

Figure S46 Formation of a mixture of $[Pd_3(\mathbf{3})_6]^{6+}$ and $[Pd_4(\mathbf{3})_8]^{8+}$ monitored by ^{19}F NMR in DMSO-d_6 (564.6 MHz, 298 K). a) *E*-**3** in DMSO-d_6; b) 5 minutes after addition of $[Pd(CH_3CN)_4](BF_4)_2$; c) 70 minutes; d) 190 minutes e) 5 hours; f) 18 hours and g) 22 hours.

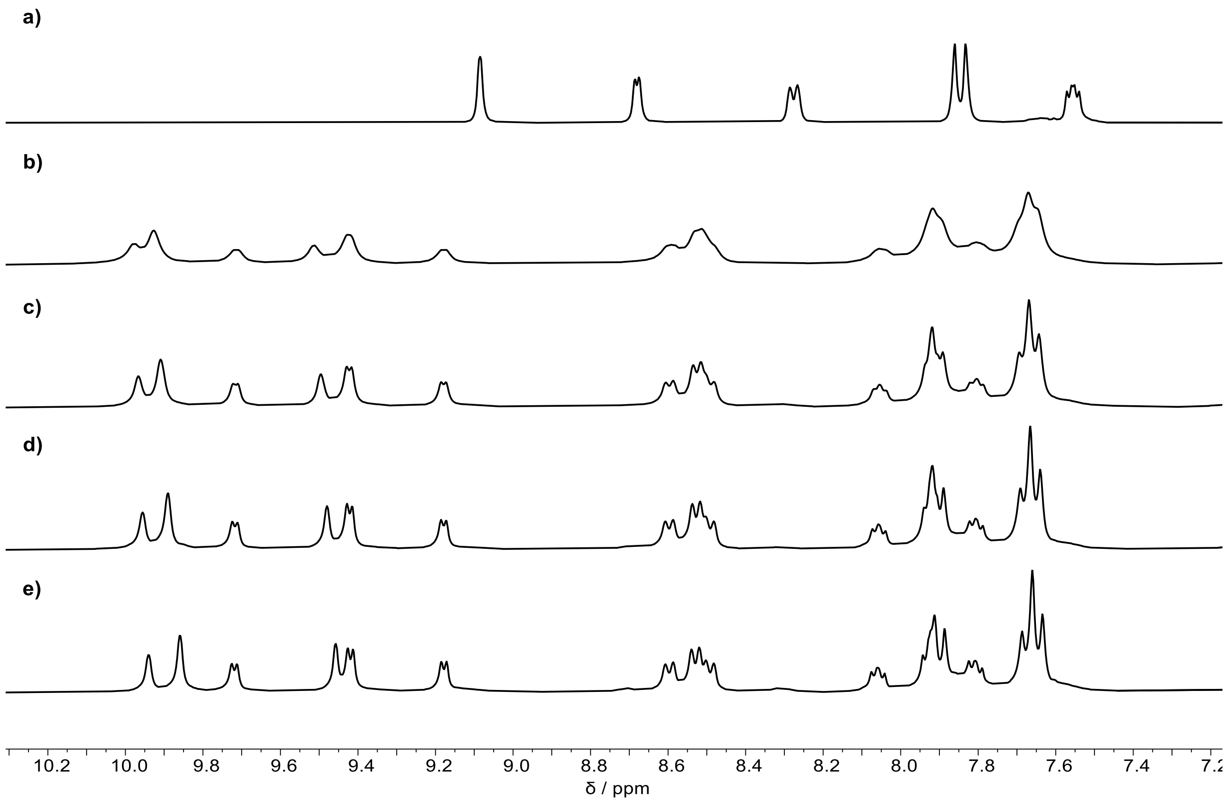

Figure S47 Formation of a mixture of $[Pd_3(\mathbf{3})_6]^{6+}$ and $[Pd_4(\mathbf{3})_8]^{8+}$ at 60 °C monitored by ^{1}H NMR (600 MHz, 333 K, DMSO-d_6) a) sample of thermally equilibrated *E*-**3**, b) mixture of $[Pd_3(\mathbf{3})_6]^{6+}$ and $[Pd_4(\mathbf{3})_8]^{8+}$, 5 minutes after adding $[Pd(CH_3CN)_4](BF_4)_2$; c) 10 minutes; d) 2 hours; e) 20 hours.

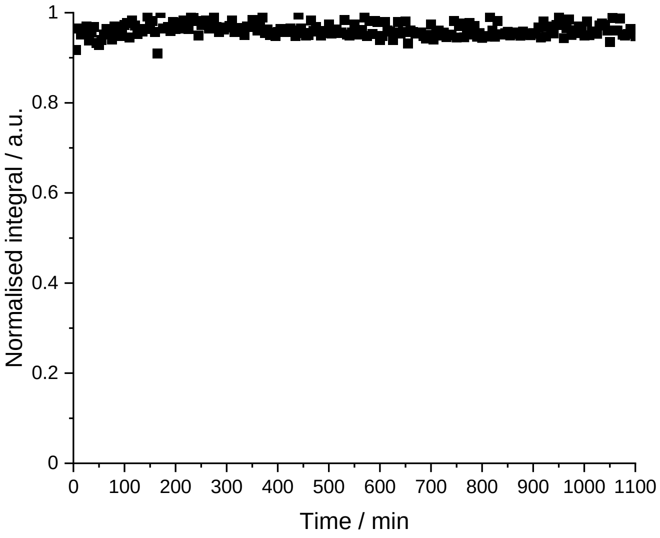

Figure S48 Normalised ^{1}H NMR integrals (600 MHz, 333 K, DMSO-d_6) monitoring the formation of $[Pd_4(\mathbf{3})_8]^{8+}$ at 60 °C following reaction of *E*-**3** with $[Pd(MeCN)_4](BF_4)_2$. Integrals of signal corresponding to $[Pd_3(\mathbf{3})_6]^{6+}$ could not be included due to overlap of signals.

7. Formation of a mixture of $[Pd_3(3)_6](BAr_F)_6$ and $[Pd_4(3)_8](BAr_F)_8$

Attempts to assemble $[Pd_3(\mathbf{3})_6]^{6+}$ and $[Pd_4(\mathbf{3})_8]^{8+}$ in CD_3CN were unsuccessful due to the poor solubility of **3**. However, if the two products were assembled first in DMSO-d_6, the products could be precipitated and redissolved in CD_3CN. We combined $[PdPy^*_4](BAr_F)_2$ (100 µL, 78.4 mM, 7.84 µmol) and *E*-**3** (500 µL, 31.3 mM, 15.7 µmol) in DMSO-d_6. The sample was left to equilibrate for 1 day. H_2O was added to precipitate a red solid. The solid was collected and washed with chloroform to remove excess Py*. The solid was dried under reduced pressure and then dissolved in CD_3CN to be monitored by ^{1}H NMR.

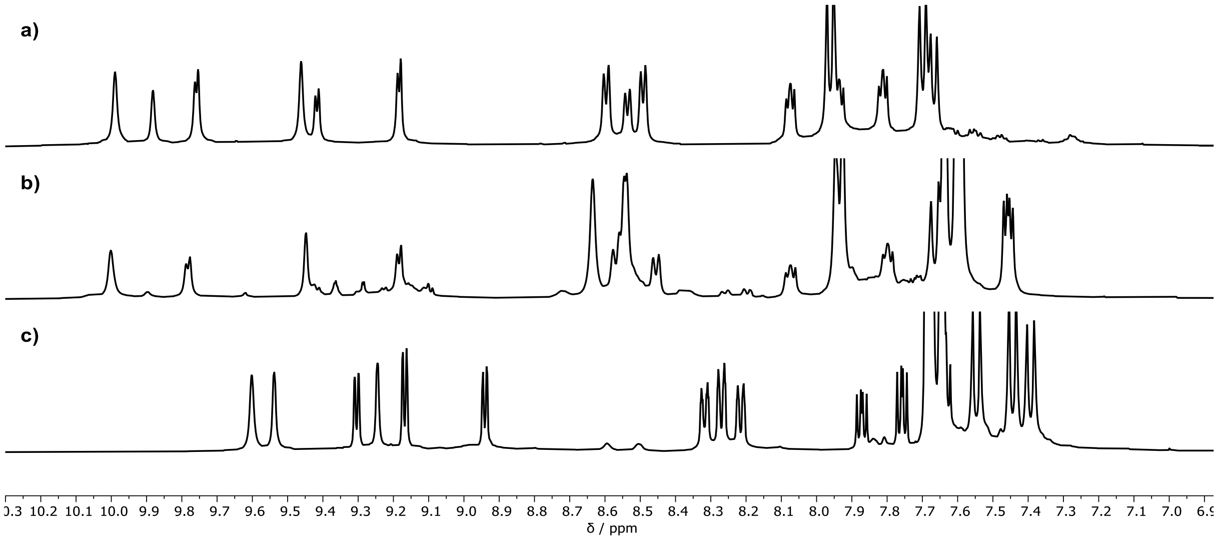

Figure S49 ^{1}H NMR spectra (500 MHz, 298 K) of a) *E*-**3** and $[Pd(MeCN)_4](BF_4)_2$ in DMSO-d_6 b) *E*-**3** and $[PdPy^*_4](BAr_F)_2$ in DMSO-d_6 c) *E*-**3** and $[PdPy^*_4](BAr_F)_2$ in CD_3CN.

8. NMR characterization of $[Pd_3(3)_6](BF_4)_6$ and $[Pd_4(3)_8](BF_4)_8$

8.1. ^{1}H, ^{13}C and ^{19}F NMR Spectra

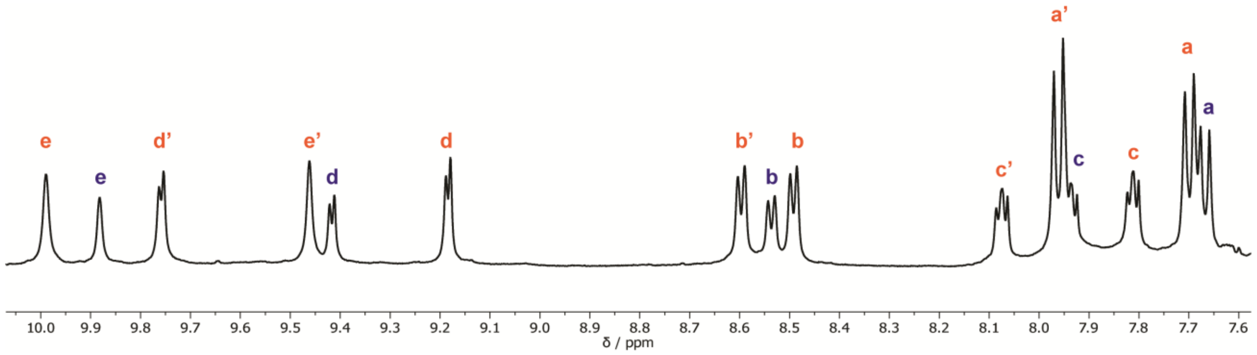

Figure S50 Partial ^{1}H NMR spectrum (DMSO-d_6, 600 MHz, 298 K) of a mixture of $[Pd_3(\mathbf{3})_6]^{6+}$ (▲) and $[Pd_4(\mathbf{3})_8]^{8+}$ (●).

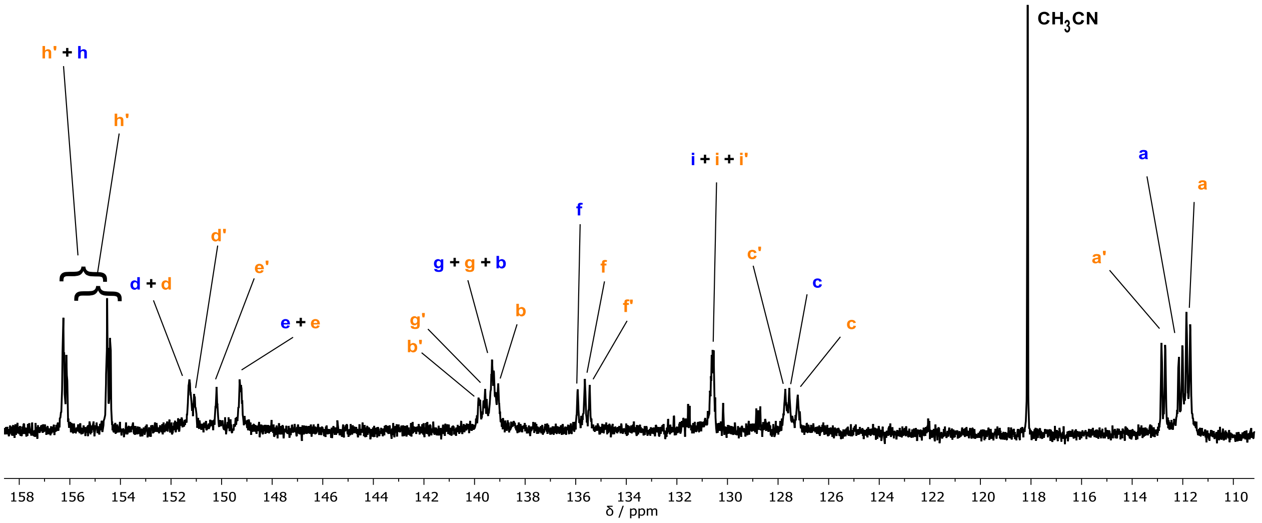

Figure S51 Partial $^{13}C\{^1H\}$ NMR spectrum (DMSO-d_6, 151 MHz, 298 K) of a mixture of $[Pd_3(\mathbf{3})_6]^{6+}$ (▲) and $[Pd_4(\mathbf{3})_8]^{8+}$ (●).

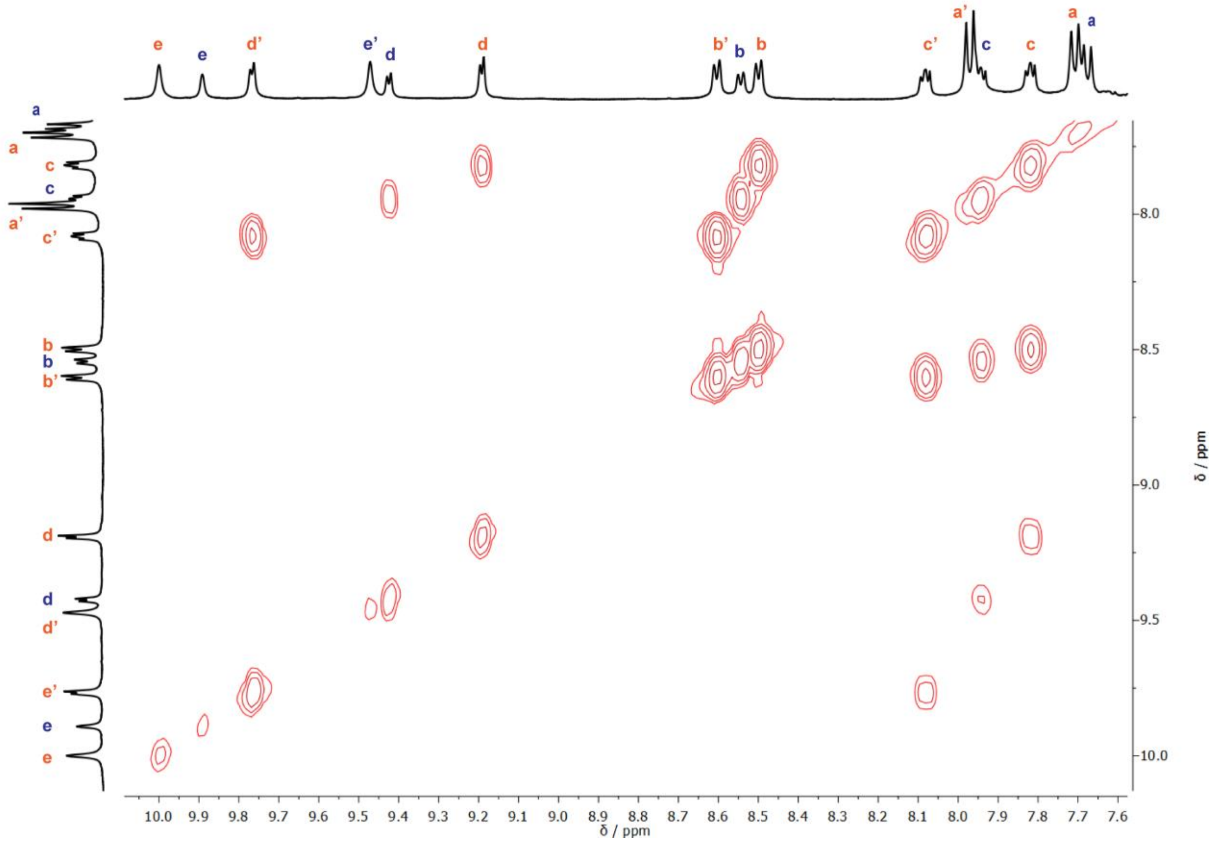

Figure S52 ^{1}H-^{1}H COSY spectrum (DMSO-d_6, 600 MHz, 298 K) of a mixture of $[Pd_3(\mathbf{3})_6]^{6+}$ and $[Pd_4(\mathbf{3})_8]^{8+}$.

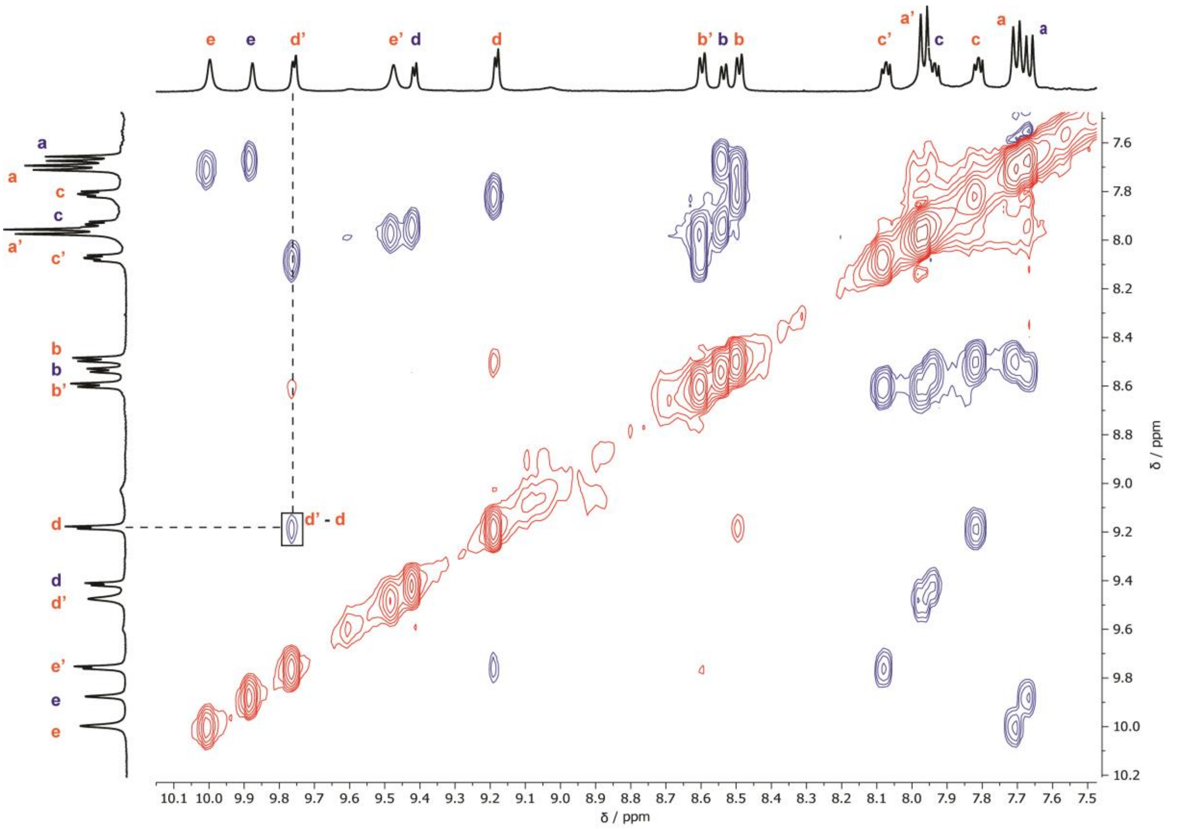

Figure S53 ^{1}H-^{1}H ROESY spectrum (DMSO-d_6, 600 MHz, 298 K) of a mixture of $[Pd_3(\mathbf{3})_6]^{6+}$ and $[Pd_4(\mathbf{3})_8]^{8+}$.

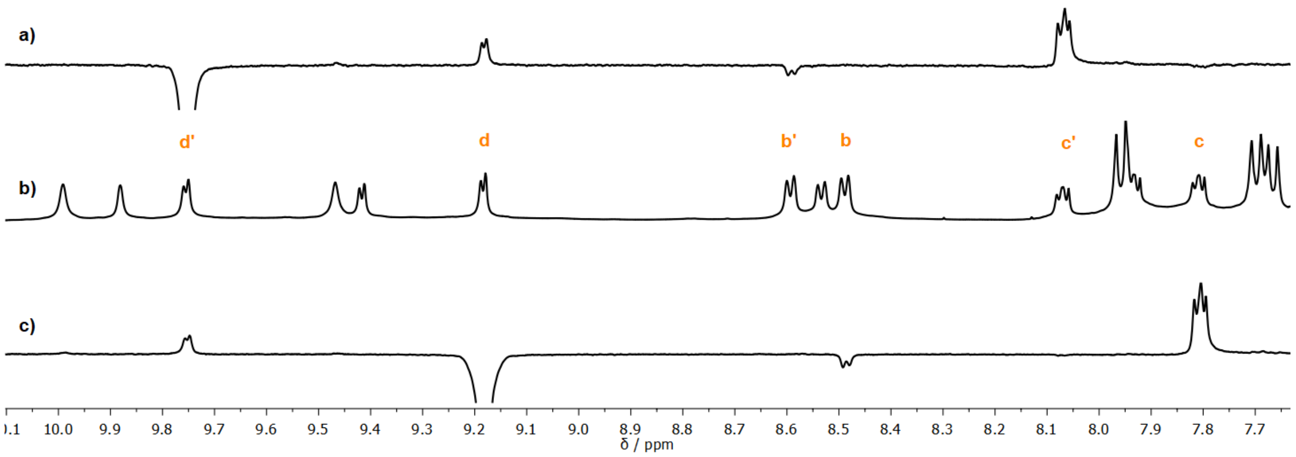

Figure S54 Selective 1D ROESY spectra (DMSO-d_6, 600 MHz, 298 K) of a mixture of $[Pd_3(\mathbf{3})_6]^{6+}$ and $[Pd_4(\mathbf{3})_8]^{8+}$. a) ROE interactions for proton $H^{d'}$. Note: the in-phase peak for proton $H^{b'}$ was assigned as a TOCSY coupling artifact;[11] b) ^1H NMR spectrum of the mixture and c) ROE interactions for proton H^d. Note: the in-phase peak for proton H^b was assigned as a TOCSY artifact.[11]

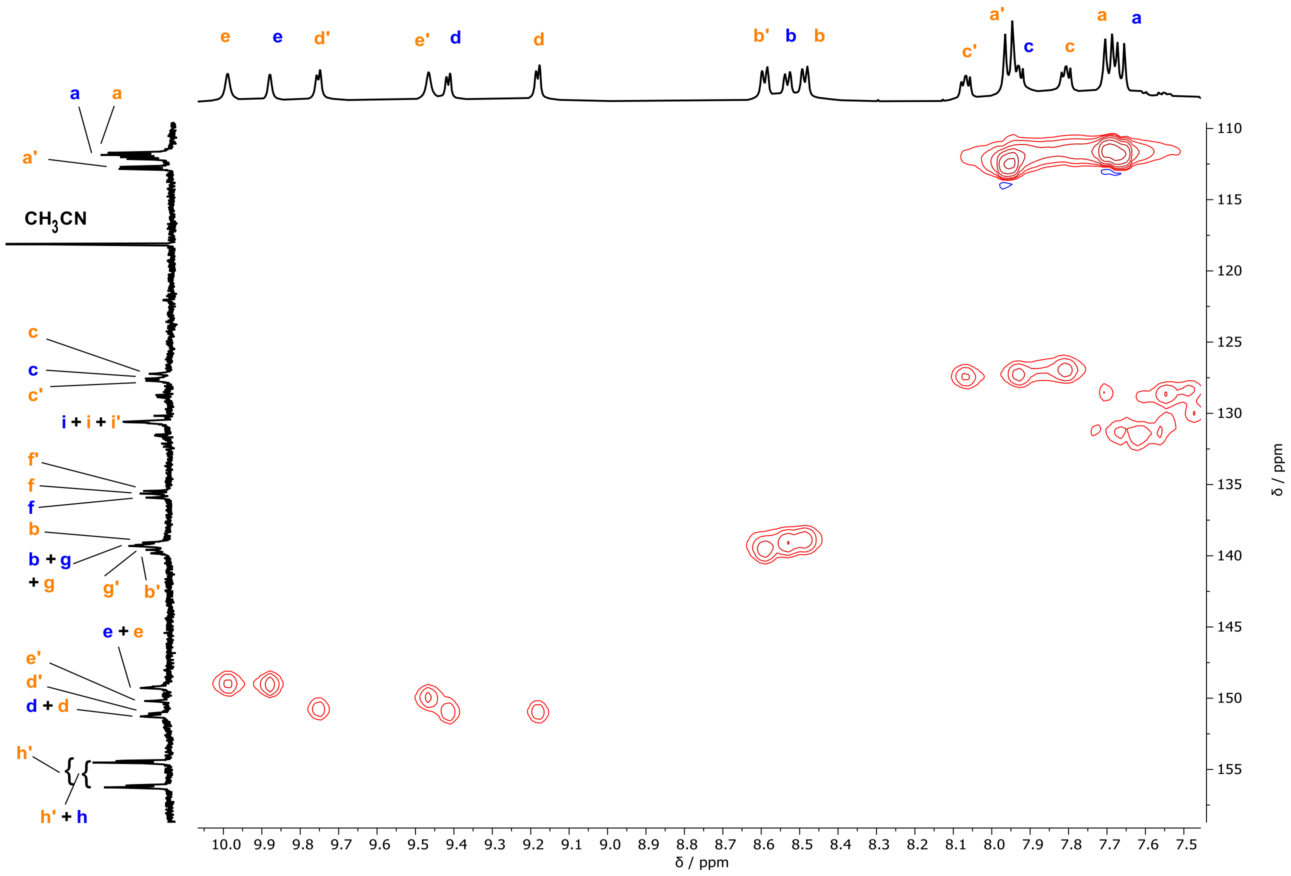

Figure S55 ^1H-^{13}C HSQC (DMSO-d_6, 600 MHz, 298 K) spectrum of a mixture of $[Pd_3(\mathbf{3})_6]^{6+}$ and $[Pd_4(\mathbf{3})_8]^{8+}$.

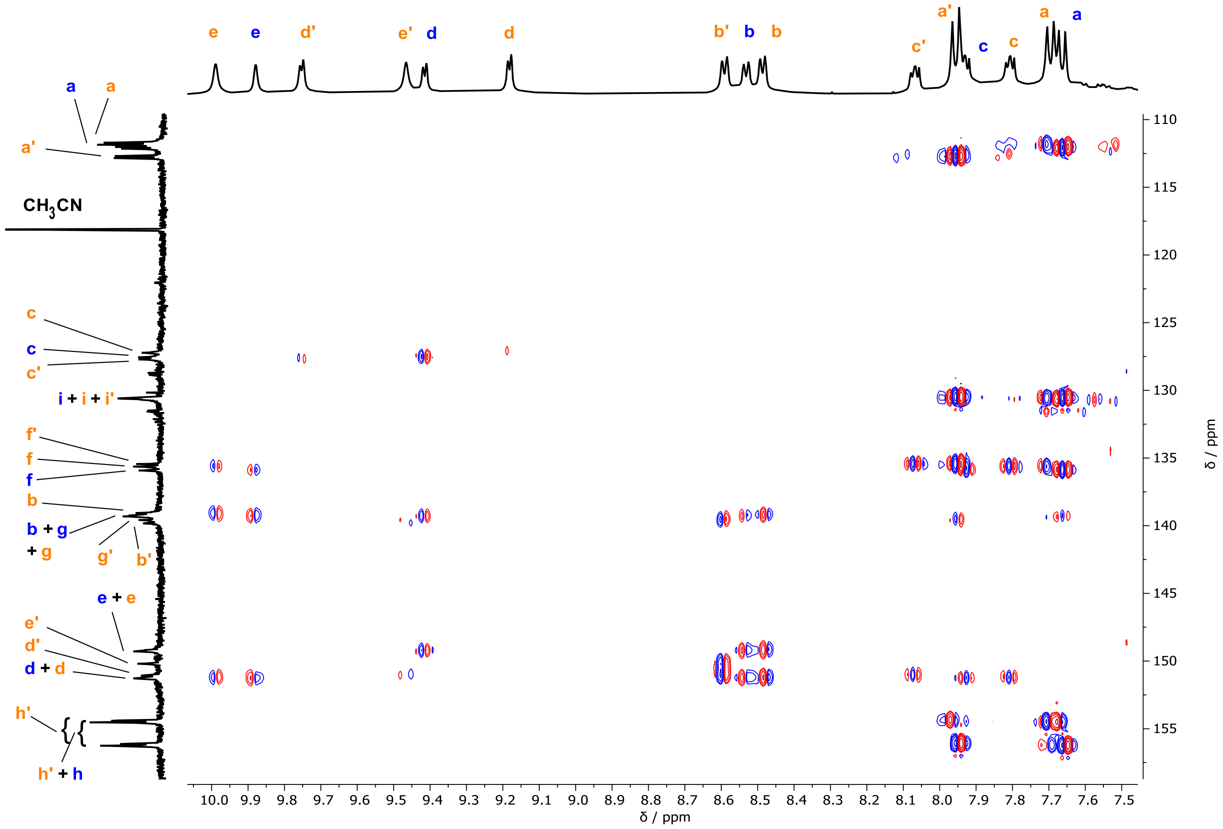

Figure S56 ^{1}H-^{13}C HMBC (DMSO-d_6, 600 MHz, 298 K) spectrum of a mixture of $[Pd_3(\mathbf{3})_6]^{6+}$ and $[Pd_4(\mathbf{3})_8]^{8+}$.

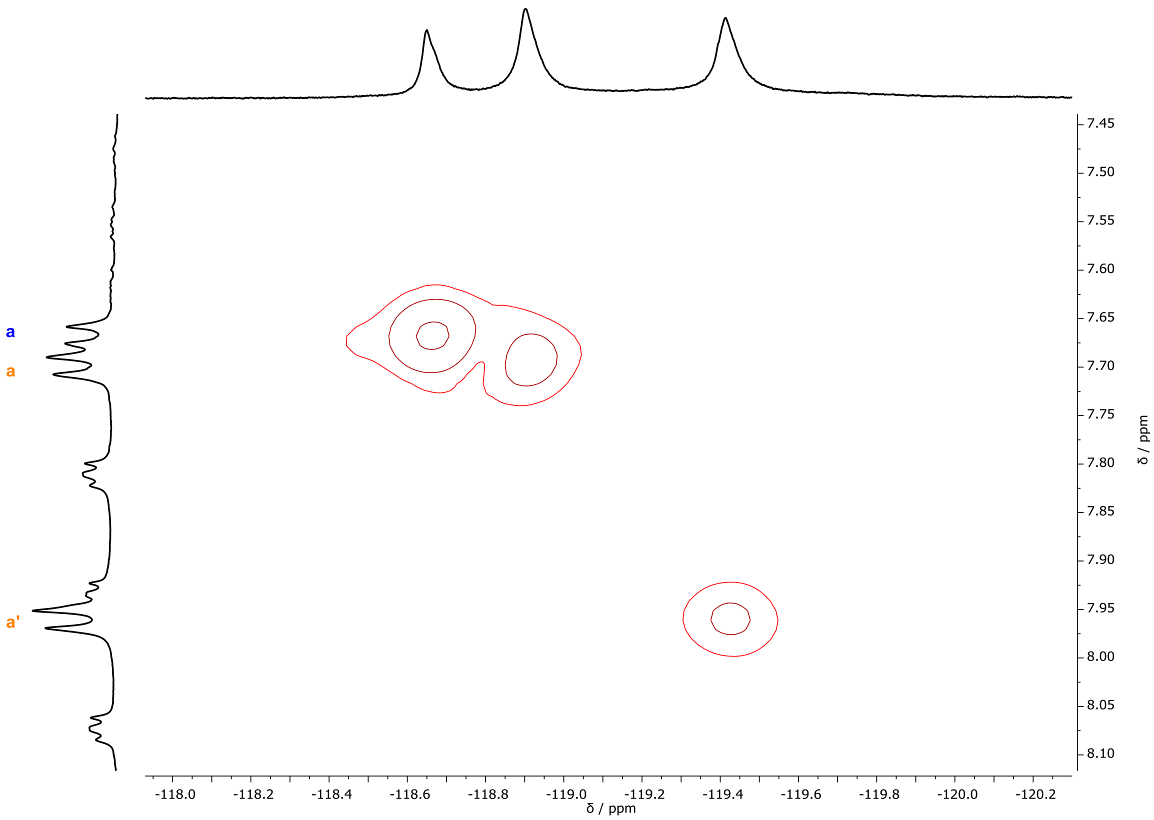

Figure S57 ^{19}F-^{1}H HETCOR (DMSO-d_6, 600 MHz, 298 K) spectrum of a mixture of $[Pd_3(\mathbf{3})_6]^{6+}$ and $[Pd_4(\mathbf{3})_8]^{8+}$.

8.2. ^{1}H diffusion NMR spectroscopy of a mixture of $[Pd_3(3)_6]^{6+}$ and $[Pd_4(3)_8]^{8+}$

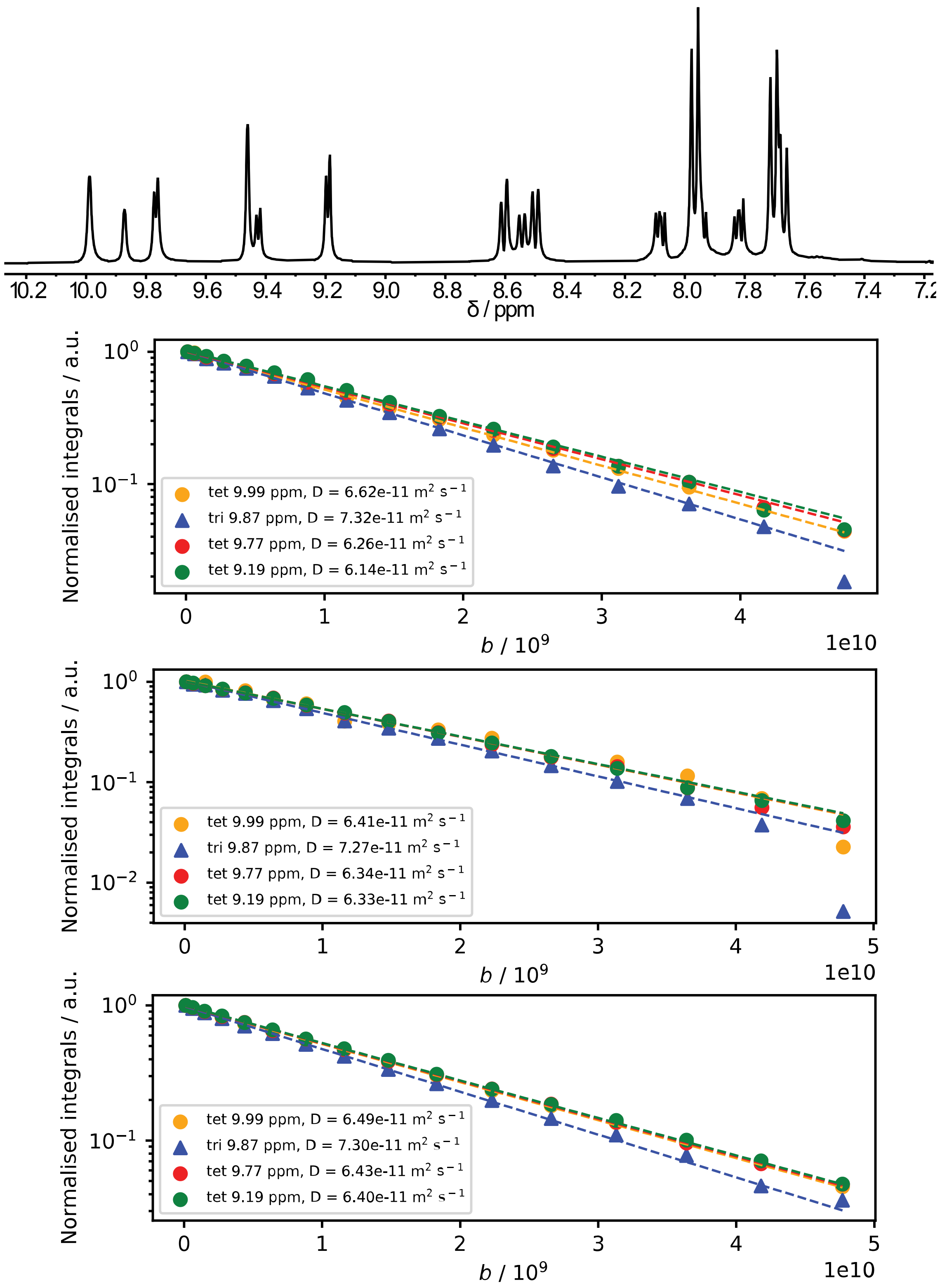

Figure S58 a) ^{1}H NMR spectrum (DMSO-d_6, 500 MHz, 298 K) of a mixture of $[Pd_3(\mathbf{3})_6]^{6+}$ and $[Pd_4(\mathbf{3})_8]^{8+}$, b) Fitting of integrals from ^{1}H NMR diffusion experiment (ste, Δ = 106 ms, δ = 5 ms), c) Fitting of integrals from ^{1}H NMR diffusion experiment (ste, Δ = 75 ms, δ = 6 ms), and d) Fitting of integrals from ^{1}H NMR diffusion experiment (ste, Δ = 60 ms, δ = 7 ms). Data were fit to a mono-exponential function using corrected gradient values ($G_{corrected} = G_{app} \times 0.909$).

Table S3 Summary of diffusion data for sample containing mixture of $[Pd_3(\mathbf{3})_6]^{6+}$ and $[Pd_4(\mathbf{3})_8]^{8+}$.

Experiment	Signal	D / $\times 10^{-11}$ m^2 s^{-1}
1	9.99 ppm	6.49
1	9.87 ppm	7.30
1	9.77 ppm	6.43
1	9.19 ppm	6.40
2	9.99 ppm	6.62
2	9.87 ppm	7.32
2	9.77 ppm	6.26
2	9.19 ppm	6.14
3	9.99 ppm	6.41
3	9.87 ppm	7.27
3	9.77 ppm	6.34
3	9.19 ppm	6.33

Species	D / $\times 10^{-11}$ m^2 s^{-1}
$[Pd_3(\mathbf{3})_6]^{6+}$	6.38 ± 0.13
$[Pd_4(\mathbf{3})_8]^{8+}$	7.30 ± 0.02

8.3. Variable temperature ^{1}H NMR data of $[Pd_3(3)_6]^{6+}$ and $[Pd_4(3)_8]^{8+}$ in DMSO-d_6

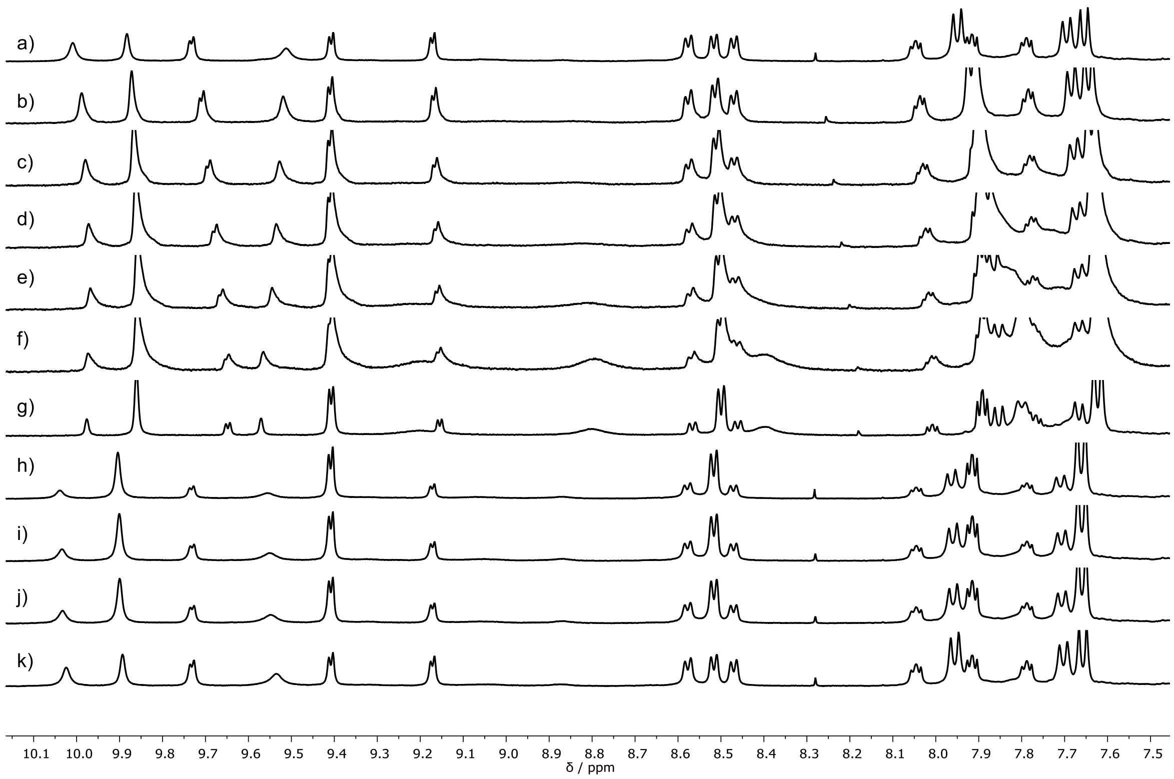

Figure S59 Variable temperature measurement of $[Pd_3(\mathbf{3})_6]^{6+}$ and $[Pd_4(\mathbf{3})_8]^{8+}$ in DMSO-d_6. a) Initial sample measured at 298 K; b) heated to 313 K; c) 323 K; d) 333 K; e) 343 K; f) 353 K; g) 353 K after re-shimming; h) returned to 298 K; i) 298 K for 1 h; j) 298 K for 1.5 h and k) 298 K after equilibration in the dark (18 h). At each temperature point the sample was equilibrated for 30 minutes.

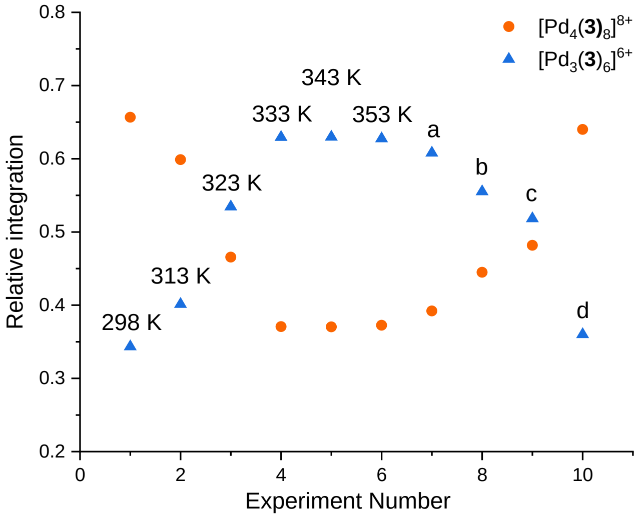

Figure S60 Variable temperature ^{1}H NMR measurement of a mixture of $[Pd_3(\mathbf{3})_6]^{6+}$ and $[Pd_4(\mathbf{3})_8]^{8+}$ in DMSO-d_6 (600 MHz). Integrals for the H^e protons on both species have been normalized relative to the overall sum and the integrations for protons H^e and $H^{e'}$ combined. Data plotted from Figure S59. Points a-d are all measured upon return to 298 K, at different time points. a) t = 0 h; b) 80 min; c) 120 min and d) 18 h.

9. ESI-MS characterization of $[Pd_3(3)_6](BF_4)_6$ and $[Pd_4(3)_8](BF_4)_8$

9.1. ESI-MS isotope patterns of $[Pd_3(3)_6]^{6+}$

Table S4 Zoom scans of select ESI-MS peaks, with simulated isotope patterns ESI-MS of [Pd_3(**3**)$_6$] double-walled triangle.

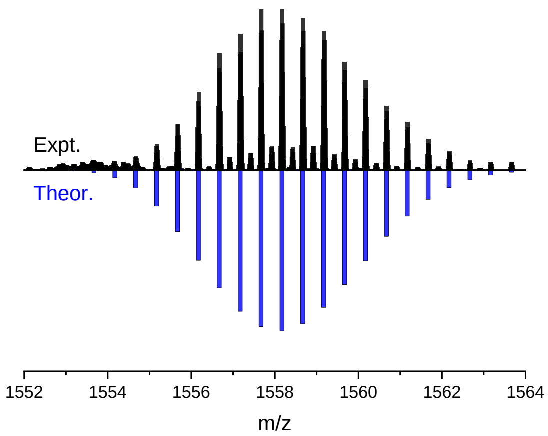	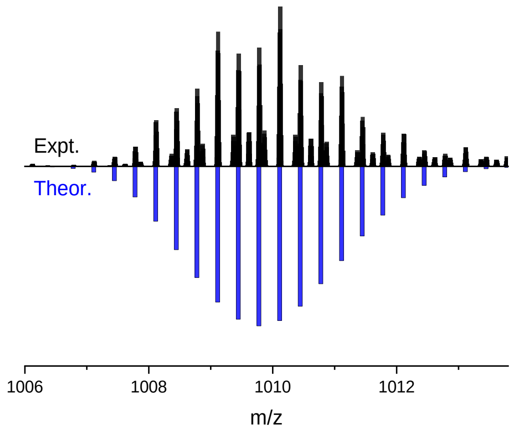
[Pd_3(**3**)$_6$ + 4BF_4]$^{2+}$ (major) and [Pd_6(**3**)$_{12}$ + 8BF_4]$^{4+}$ (minor)	$[Pd_3(3)_6 + 3BF_4]^{3+}$
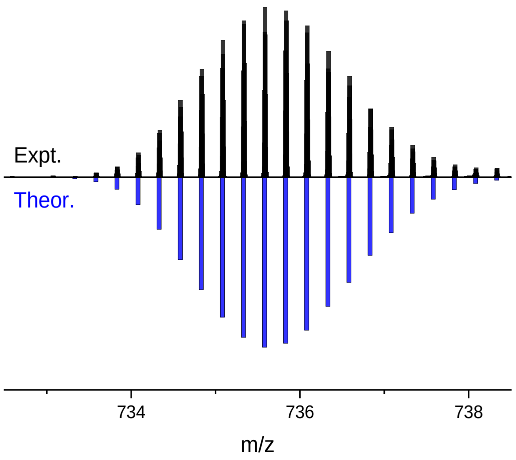	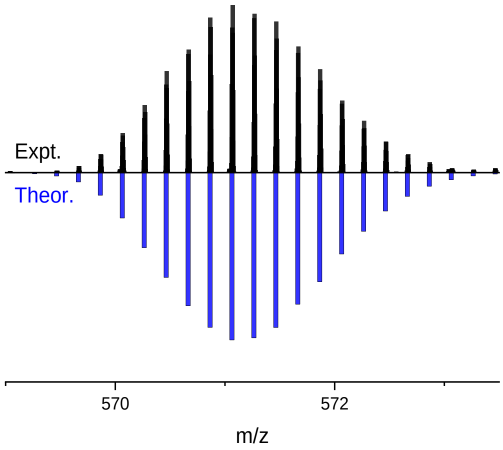
$[Pd_3(3)_6 + 2BF_4]^{4+}$	$[Pd_3(3)_6 + BF_4]^{5+}$

9.2. ESI-MS isotope patterns of $[Pd_4(3)_8]^{8+}$

Table S5 Zoom scans of select ESI-MS peaks, with simulated isotope patterns ESI-MS of $[Pd_4(\mathbf{3})_8]$ double-walled triangle.

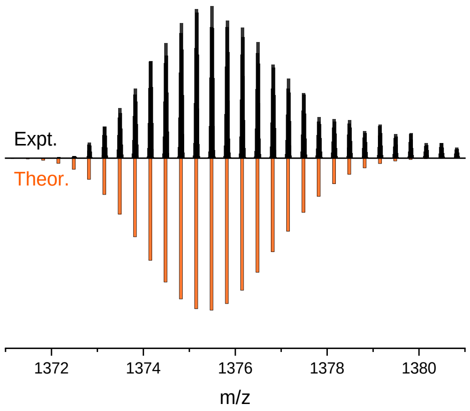	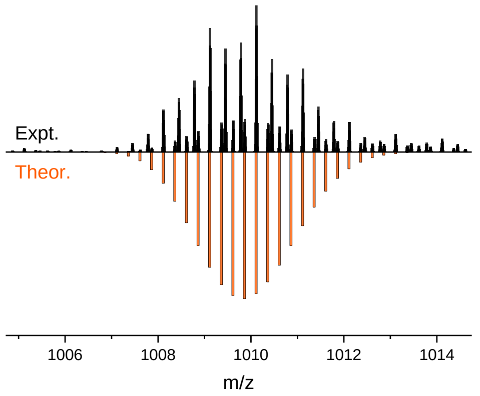
$[Pd_4(3)_8+5BF_4]^{3+}$	$[Pd_4(3)_8+4BF_4]^{4+}$
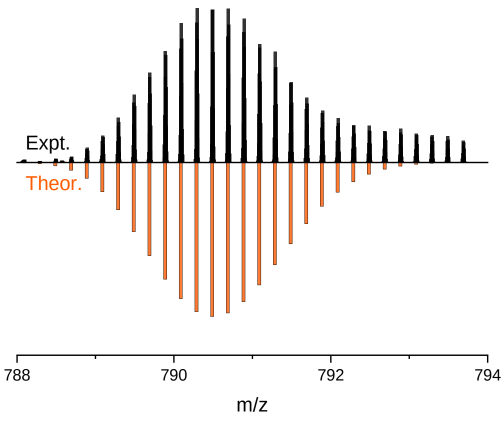	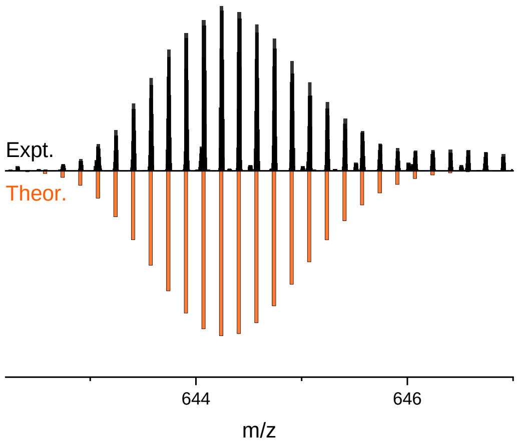
$[Pd_4(3)_8+3BF_4]^{5+}$	$[Pd_4(3)_8+2BF_4]^{6+}$
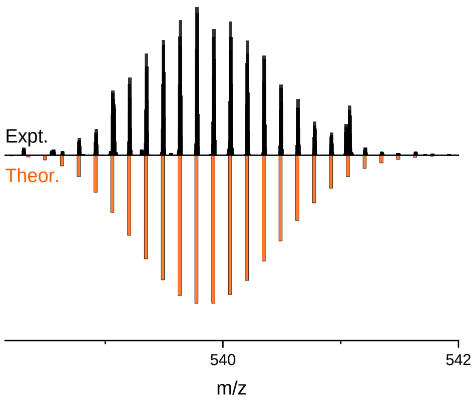	
$[Pd_{34}(3)_8+BF_4]^{7+}$	

10. Assigned characterisation of $[Pd_3(3)_6]^{6+}$

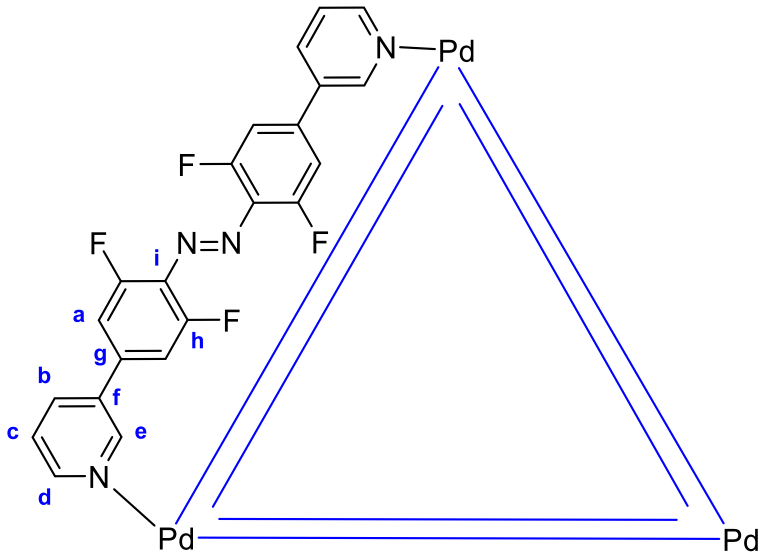

Table S6 ^{1}H, ^{19}F and ^{13}C NMR assignments for the self-assembled $[Pd_3(\mathbf{3})_6]^{6+}$ species.

^{1}H			^{13}C	
δ / ppm	Assignment	*J*-Coupling / Hz	δ / ppm	Assignment
9.88 (s, 12H)	H^e	-	155.4	C^h (*J* = 261, 5 Hz)
9.42 (d, 12H)	H^d	5.8	151.3	C^d
8.54 (d, 12H)	H^b	8.1	149.3	C^e
7.94 (dd, 12H)	H^c	-	139.4	C^{b+g}
7.67(d, 24H)	H^a	11	135.9	C^f
			130.6	C^i
^{19}F			127.6	C^c
-118.7			112.1	C^a (*J* = 22 Hz)

10.1. HR-ESI-MS for $([Pd_3(3)_6](BF_4)_{(6-n)})^{n+}$

Table S7 High resolution ESI-MS assignments for the self-assembled $[Pd_3(\mathbf{3})_6]^{6+}$ species

HR-ESI MS for $([Pd_3(3)_6](BF_4)_{(6-n)})^{n+}$		
Found m/z	Assignment	Required m/z
1558.1789	$[Pd_3(\mathbf{3})_6 + 4BF_4^-]^{2+}$	1558.1709
1010.1201(overlapping)	$[Pd_3(\mathbf{3})_6 + 3BF_4^-]^{3+}$	1009.77842
	$[Pd_4(\mathbf{3})_8 + 4BF_4^-]^{4+}$	1009.86176
735.5887	$[Pd_3(\mathbf{3})_6 + 2BF_4^-]^{4+}$	735.58219
571.2690	$[Pd_3(\mathbf{3})_6 + BF_4^-]^{5+}$	571.26445

11. Assigned characterization of $[Pd_4(3)_8]^{8+}$

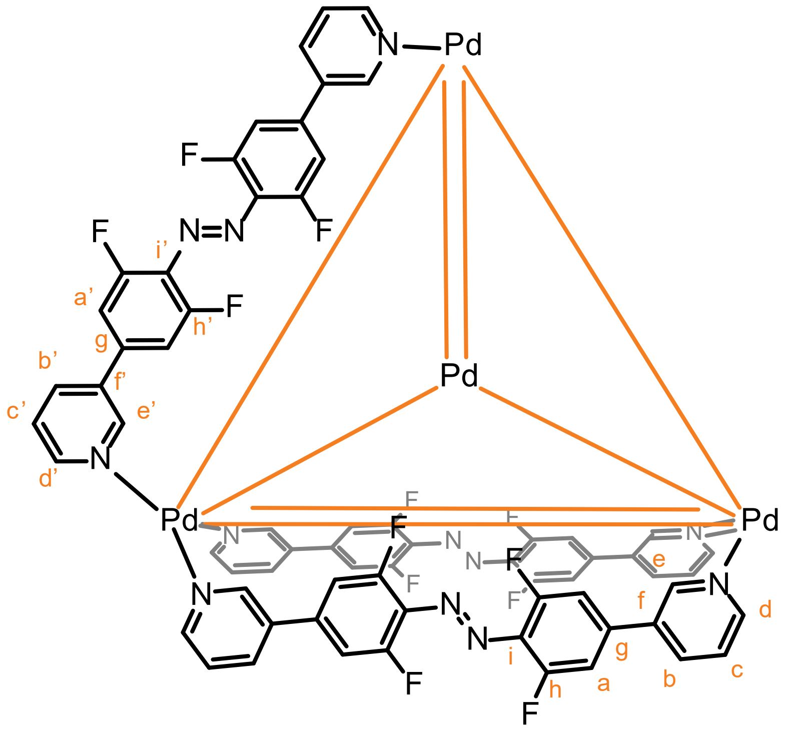

Table S8 ^{1}H, ^{19}F and ^{13}C NMR assignments for the self-assembled $[Pd_4(\mathbf{3})_8]^{8+}$ species

^{1}H			^{13}C	
δ / ppm	Assignment	*J*-Coupling / Hz	δ / ppm	Assignment
9.99 (s, 1H)	H^{e}	-	155.4	C^{h} (*J* = 261, 5 Hz)
9.76 (d, 1H)	$H^{d'}$	5.7	155.3	$C^{h'}$ (*J* = 261, 5 Hz)
9.42 (s, 1H)	$H^{e'}$	-	151.3	C^{d}
9.18 (d, 1H)	H^{d}	5.7	151.1	$C^{d'}$
8.60 (d, 1H)	$H^{b'}$	8.1	150.2	$C^{e'}$
8.49 (d, 1H)	H^{b}	8.0	149.3	C^{e}
8.07 (dd, 1H)	$H^{c'}$	8.1, 5.7	139.8	$C^{b'}$
7.96 (m, 2H)	$H^{a'}$	11	139.1	C^{b}
7.81 (dd, 1H)	H^{c}	8.0, 5.7	135.6	C^{f}
7.69 (m, 2H)	H^{a}	10	135.4	$C^{f'}$
			130.6	C^{g} + $C^{g'}$
			127.7	$C^{c'}$
^{19}F			127.2	C^{c}
-118.9 (F)			112.8	$C^{a'}$
-119.4 (F')			111.8	C^{a}

11.1. HR-ESI-MS for ($[Pd_4(3)_8](BF_4)_{(8-n)})^{n+}$

Table S9 High resolution ESI-MS assignments for the self-assembled $[Pd_4(\mathbf{3})_8]^{8+}$ species

HR-ESI MS for ($[Pd_4(3)_8](BF_4)_{(8-n)})^{n+}$		
Found m/z	Assignment	Required m/z
1375.4916	$[Pd_4(\mathbf{3})_8 + 5BF_4^-]^{3+}$	1375.4845.
1010.1201 (overlapping)	$[Pd_3(\mathbf{3})_6 + 3BF_4^-]^{3+}$	1009.77842
	$[Pd_4(\mathbf{3})_8 + 4BF_4^-]^{4+}$	1009.86176
790.4952	$[Pd_4(\mathbf{3})_8 + 3BF_4^-]^{5+}$	790.4881
644.4114	$[Pd_4(\mathbf{3})_8 + 2BF_4^-]^{6+}$	644.2390
539.7804	$[Pd_4(\mathbf{3})_8 + BF_4^-]^{7+}$	539.7753

12. Controlled degradation of self-assembled species with DMAP

To assign the isomer of **3** in the self-assembled structures a degradation experiment was performed, based on the affinity of 4-dimethylaminopyridine (DMAP) for palladium(II). $[Pd(CH_3CN)_4](BF_4)_2$ (2.2 mg, 4.9 µmol) was added to *E*-**3** (4.0 mg, 9.7 µmol) dissolved in DMSO-*d6* (500 µL) and allowed to equilibrate in the dark to give a mixture of $[Pd_3(\mathbf{3})_6]$ and $[Pd_4(\mathbf{4})_8]$. DMAP (4.8 mg, 39 µmol, 8 equiv.) dissolved in DMSO-*d6* (25 µL) was added to the NMR tube and the reaction monitored by ^{1}H and ^{19}F NMR. Upon addition of DMAP the signals corresponding to *E*-**3** are regenerated, with additional signals corresponding to $[Pd(DMAP)_4]^{2+}$ and free DMAP in solution.

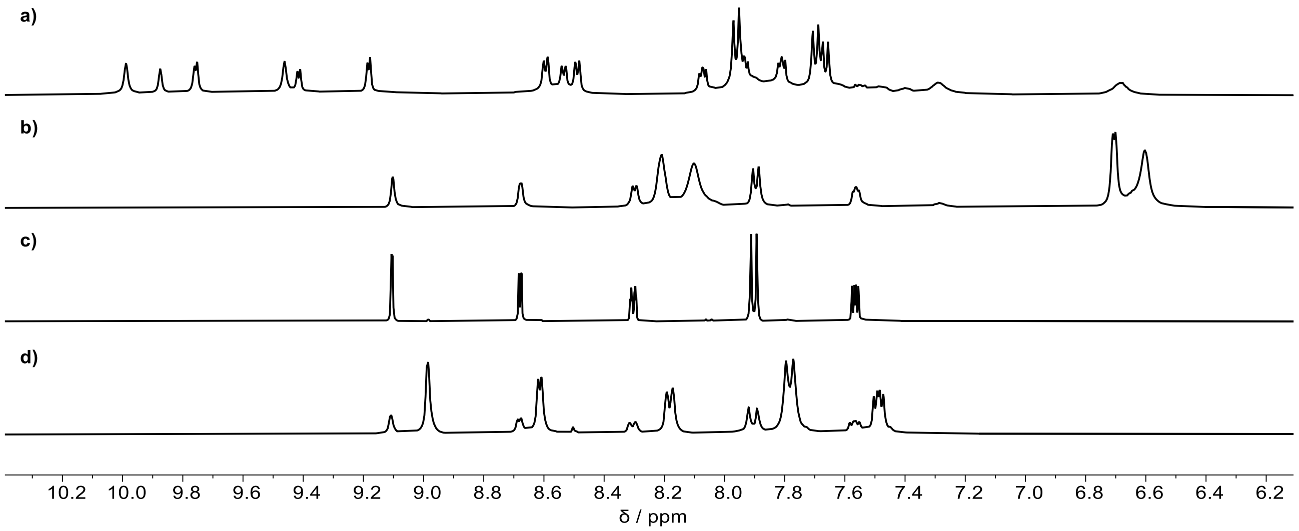

Figure S61 ^{1}H NMR (DMSO-*d*$_6$, 600 MHz, 298 K) showing the degradation of the self-assemblies to give only *E*-**3**. a) the mixture of $[Pd_3(\mathbf{3})_6]^{6+}$ and $[Pd_4(\mathbf{3})_8]^{8+}$ formed after addition of $[Pd(CH_3CN)_4](BF_4)_2$ to *E*-**3** and 24 h equilibration in the dark. Red asterisks indicate impurities present in the $[Pd(CH_3CN)_4](BF_4)_2$. b) Upon addition of DMAP and equilibration in the dark for 30 minutes, c) authentic sample of *E*-**3** and d) authentic sample of a mixture of *Z*-**3** and *E*-**3**.

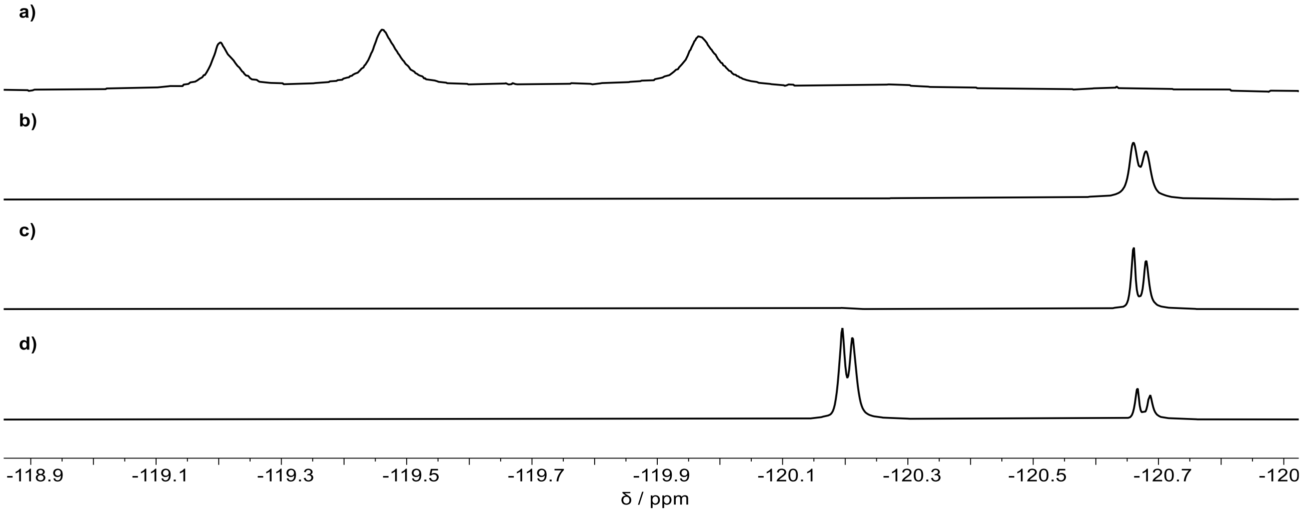

Figure S62 ^{19}F NMR (DMSO-*d*$_6$, 564.6 MHz, 298 K) showing the degradation of the self-assemblies. a) mixture of $[Pd_3(\mathbf{3})_6]^{6+}$ and $[Pd_4(\mathbf{3})_8]^{8+}$ formed after addition of $[Pd(CH_3CN)_4](BF_4)_2$ to *E*-**3** and 24 h equilibration in the dark. Red asterisks indicate impurities present in the $[Pd(CH_3CN)_4](BF_4)_2$. b) Upon addition of DMAP and equilibration in the dark for 30 minutes, c) authentic sample of *E*-**3** and d) authentic sample of a mixture of *Z*-**3** and *E*-**3**.

13. Formation of self-assembled products upon combination of $[Pd(CH_3CN)_4](BF_4)_2$ and *Z*-3

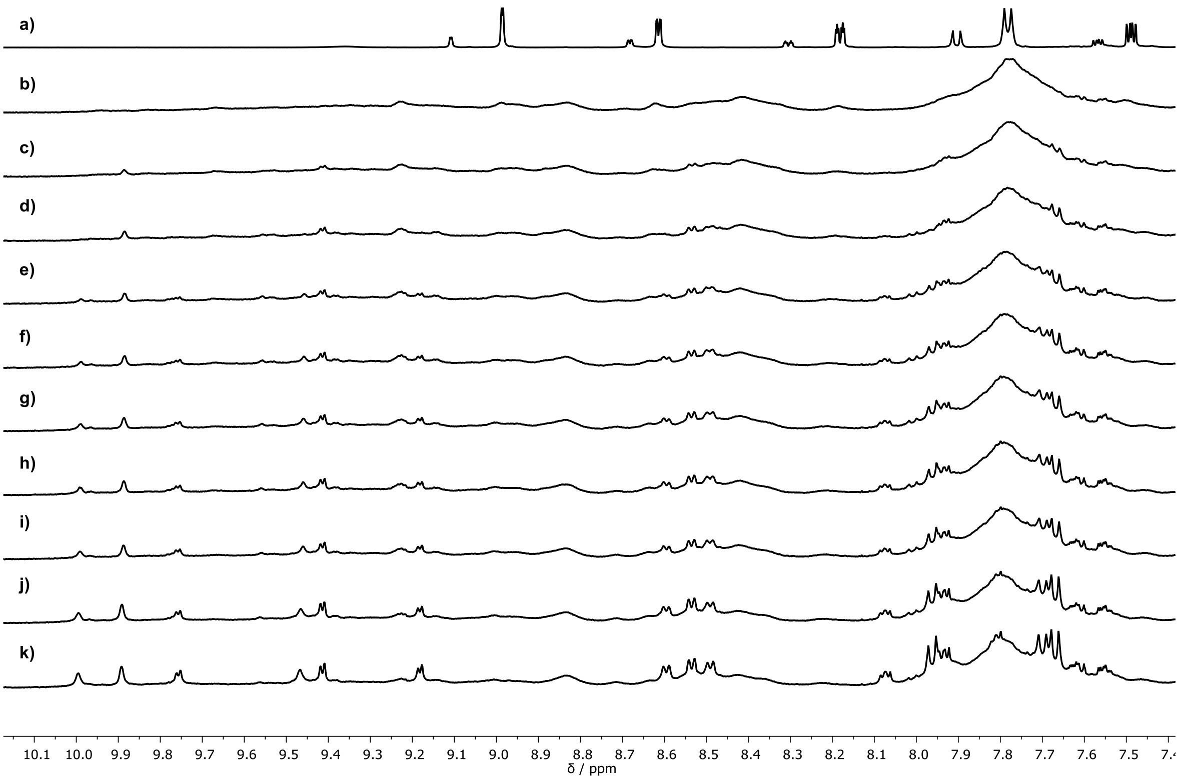

Figure S63 Combination of a *Z*-isomer enriched solution of **3** (1.0 equivalent, 4.0 mg, 9.8 µmol, 20 mmol) and $[Pd(CH_3CN)_4](BF_4)_2$ (0.51 equivalents, 2.2 mg, 5.0 µmol, 9.9 mmol) in DMSO-*d*6. a) ^1H NMR of **3** (600 MHz, 298 K) after irradiation with a 530 nm LED for 85 minutes. The mixture comprises 80% *Z*-**3** as measured by ^{19}F NMR integrations; b) upon addition of $[Pd(CH_3CN)_4](BF_4)_2$; c) 15 minutes after addition; d) after 75 minutes; e) 9.5 hours; f) 20 h; g) 51 h; h) 59 h; i) 69 h; j) 5 days and k) 9 days. Between measurements samples was kept at RT in the dark.

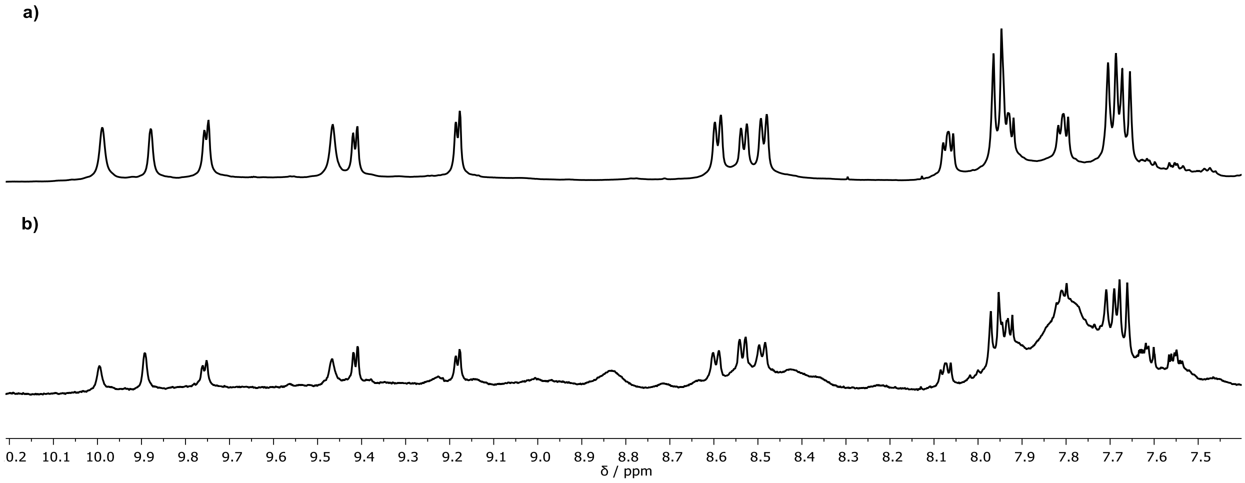

Figure S64 ^1H NMR spectra (DMSO-*d*6, 600 MHz, 298 K) comparing (a) a known mixture of $[Pd_3(\mathbf{3})_6]^{6+}$ and $[Pd_4(\mathbf{3})_8]^{8+}$ formed from *E*-**3** and (b) the mixture of *Z*-**3** and $[Pd(CH_3CN)_4](BF_4)_2$ after 9 days (k from Figure S56 above).

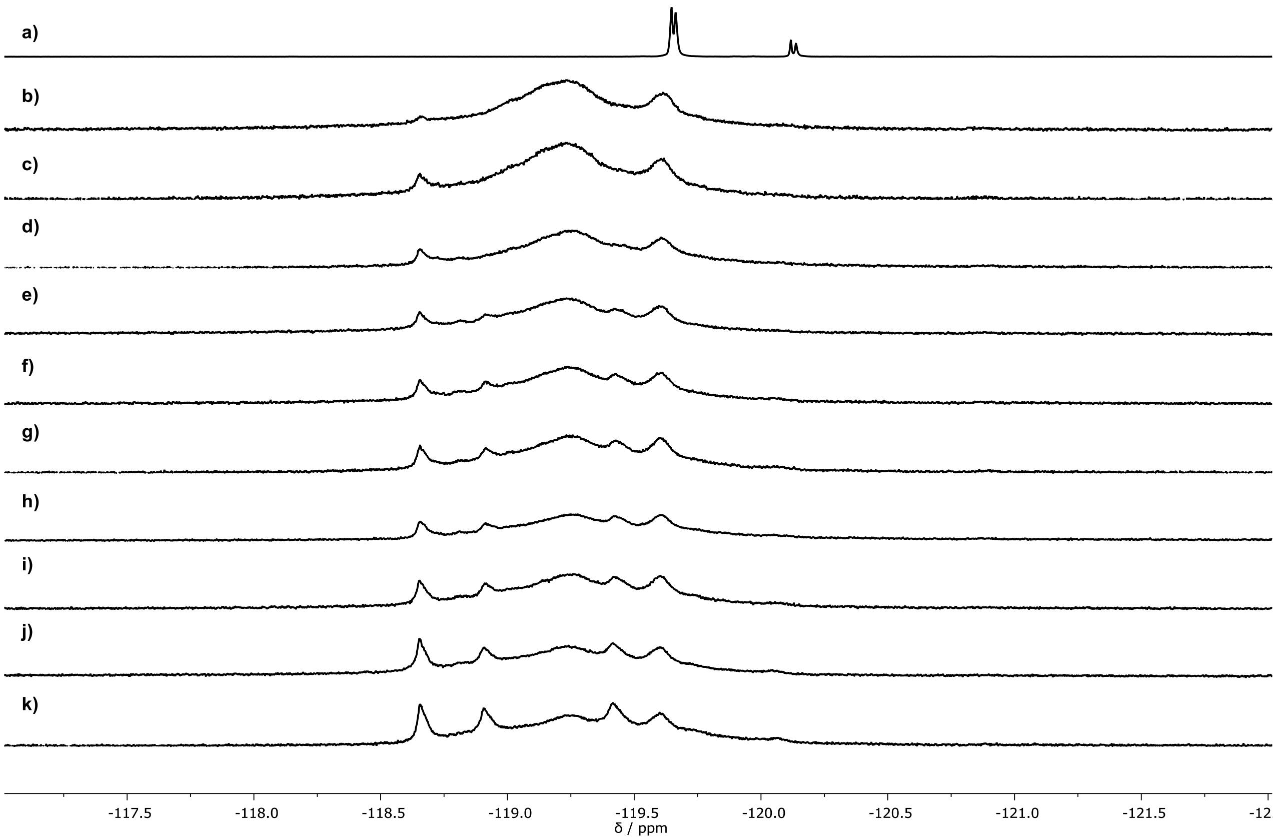

Figure S65 Combination of a *Z*-isomer enriched solution of **3** (1.0 equivalent, 4.0 mg, 9.8 µmol, 20 mmol) and $[Pd(CH_3CN)_4](BF_4)_2$ (0.51 equivalents, 2.2 mg, 5.0 µmol, 9.9 mmol) in DMSO-d_6. a) ^{19}F NMR of 3 (564.6 MHz, 298 K) after irradiation with a 530 nm LED for 85 minutes. The mixture comprises 81% *Z*-**3** as measured by NMR integration; b) upon addition of [Pd(CH3CN)4](BF4)2; c) 15 minutes after addition; d) 75 minutes; e) 9.5 hours; f) 20 h; g) 51 h; h) 59 h; i) 69 h; j) 5 days and k) 9 days. Between measurements sample was kept at RT in the dark.

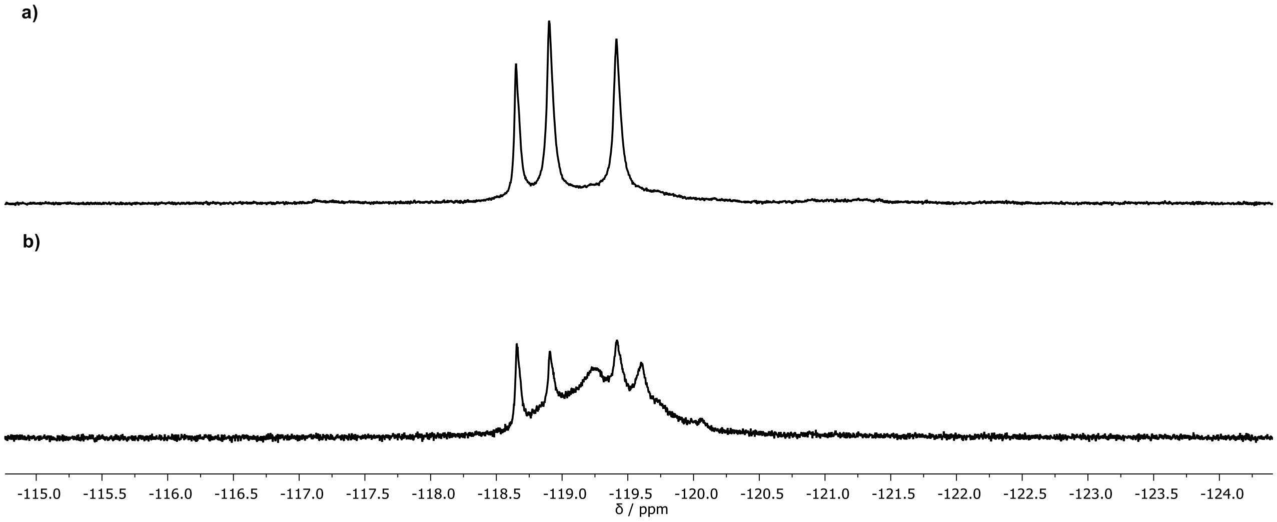

Figure S66 ^{19}F NMR (DMSO-d_6, 564.6 MHz, 298 K) comparison of (a) a known mixture of $[Pd_3(\mathbf{3})_6]^{6+}$ and $[Pd_4(\mathbf{3})_8]^{8+}$ formed from *E*-**3** and (b) the mixture of enriched $[Pd_3(\mathbf{3})_6]^{6+}$ and $[Pd_4(\mathbf{3})_8]^{8+}$ after 9 days.

14. Binding measurements between palladium(II) and 3-pyridyl ligands

14.1. Determination of binding constants using ITC

We determined the binding constant of between pyridyl ligands and palladium(II) using isothermal titration calorimetry (ITC) techniques (Table S10). A solution of 3-methylpyridine in DMSO was added to a solution of [Pd(ttpy)(DMSO)]$(BF_4)_2$ in DMSO. The experiment was repeated using DMSO as the titrant to determine the associated heat of solvation. The resulting heat transferred was measured and recorded as a thermograph. The thermographs were processed, and the parameters of binding were d etermined using NanoAnalyze software (Figure S67, Figure S68 and Figure S69). The baseline of the thermogram was automatically corrected and the integration region of each addition were manually set to provide the heat transferred per mole of titrant injected. The corresponding data was fit to the independent model provided by the NanoAnalyse software to determine the physical parameters of binding: K_a, ΔH, ΔG and ΔS at 298 K.

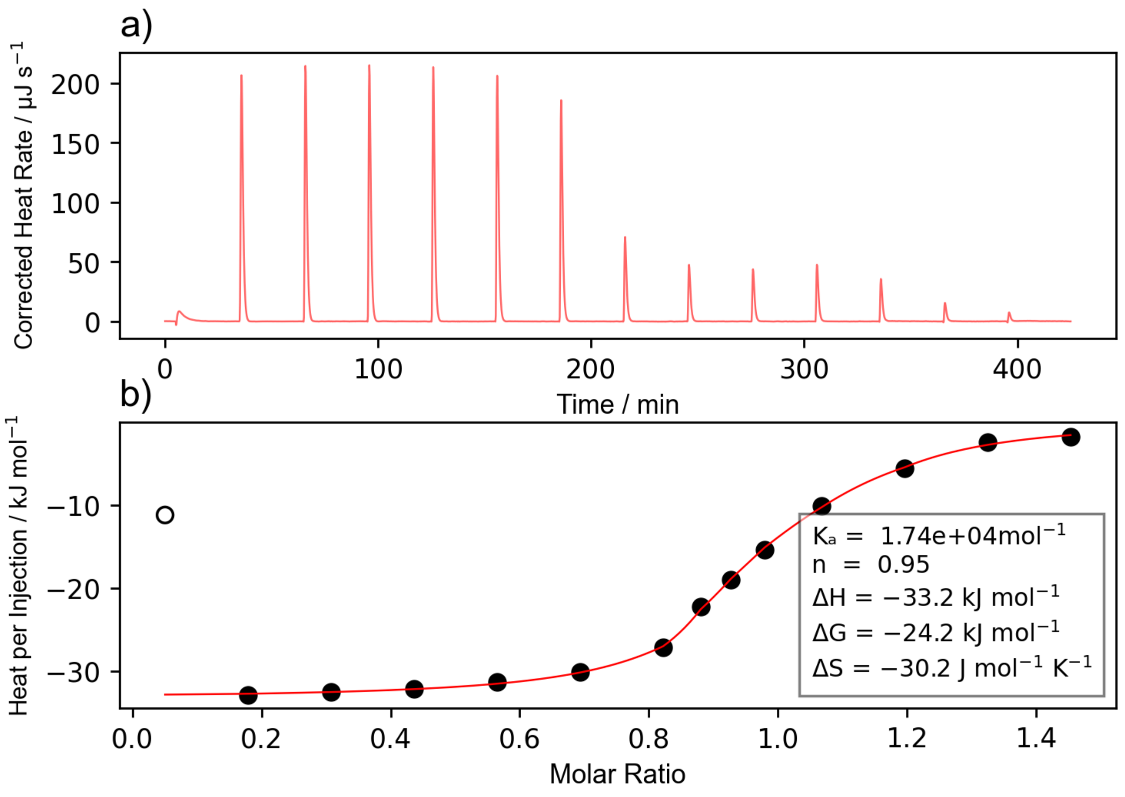

Figure S67 a) Raw thermograph and b) integrated heat data for titration of 3-methylpyridine (99 mM) into [Pd(ttpy)(DMSO)]$(BF_4)_2$ (5.0 mM) performed at 298 K. The first data point was not included for determining fit.

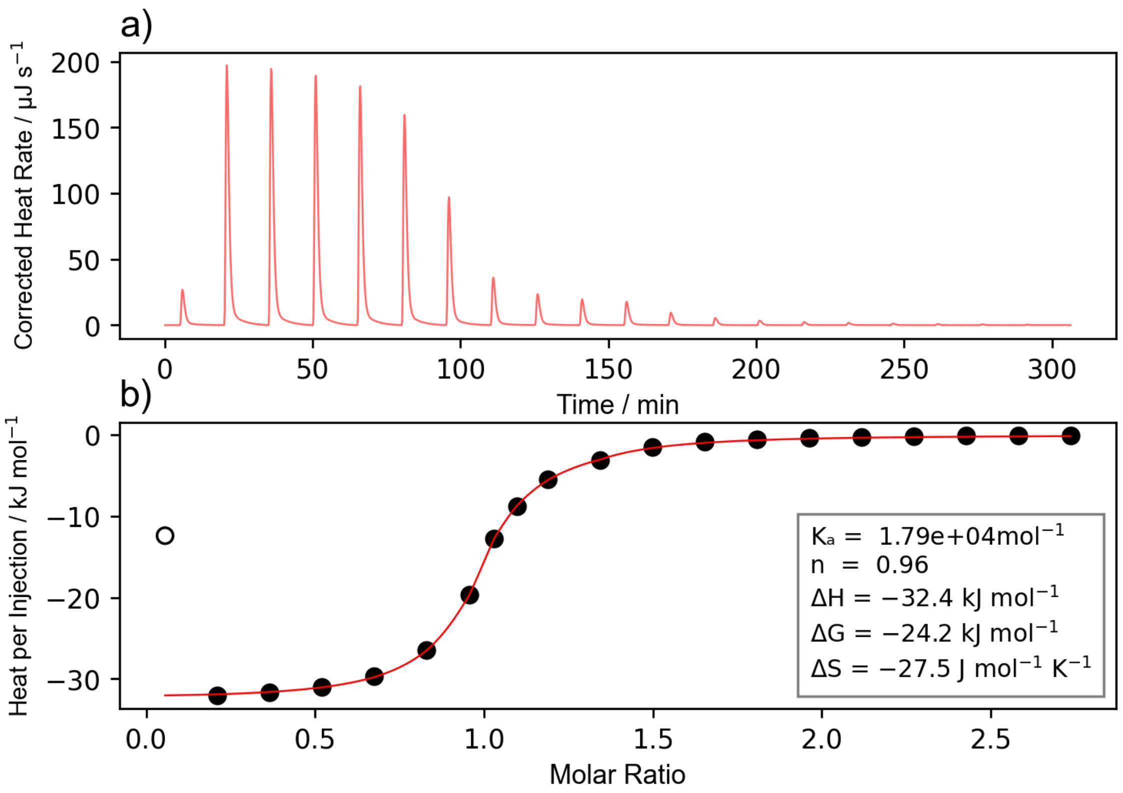

Figure S68 a) Raw thermograph and b) integrated heat data for titration of 3-methylpyridine (102 mM) into [Pd(ttpy)(DMSO)]$(BF_4)_2$ (4.3 mM) performed at 298 K. The first data point was not included for determining fit.

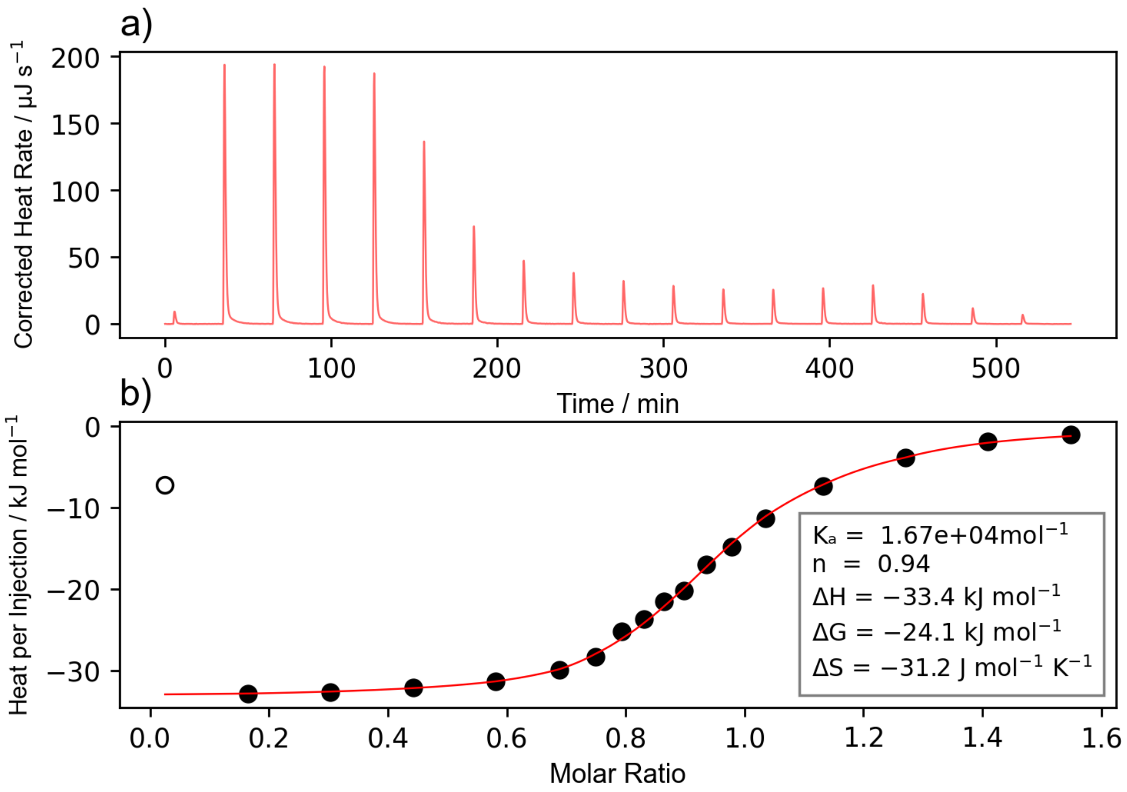

Figure S69 a) Raw thermograph and b) integrated heat data for titration of 3-methylpyridine (100 mM) into [Pd(ttpy)(DMSO)]$(BF_4)_2$ (4.6 mM) performed at 298 K. The first data point was not included for determining fit.

Table S10 Summary of ITC titration data collected for the addition of 3-methylpyridine into $[Pd(ttpy)(DMSO)](BF_4)_2$.

Experiment	K_a / mol^{-1}	ΔH / kJ·mol^{-1}	ΔG / kJ·mol^{-1}	ΔS / J·mol^{-1}·K^{-1}
1	1.74 ×10^4	-33.2	-24.2	-30.3
2	1.80 ×10^4	-32.5	-24.3	-27.6
3	1.67 ×10^4	-33.4	-24.0	-31.4
Average	1.73 (± 0.05) ×10^4	-33.0 ± 0.4	-24.2 ± 0.1	-29.8 ± 2.2

We used ITC to measure a binding between ligand **3** and palladium(II) (Figure S70). The resulting integrated heat data indicates that binding between the two species is weak. The poor solubility of **3** prevented the use of more concentrated samples of titrant and titrand. A solution of $[Pd(ttpy)(DMSO)](BF_4)_2$ was added to a solution of ligand **3**. Due to weak binding the sample of $[Pd(ttpy)(DMSO)](BF_4)_2$ was prepared with a high concentration which resulted in the precipitation of the Pd(II) complex. The consequence of this was observed when fitting the data to an independent model using NanoAnalyse, where the determined number of binding sites, n, was unreasonable. To correct for this, an additional variable was added to the fitting model: concentration correction factor.

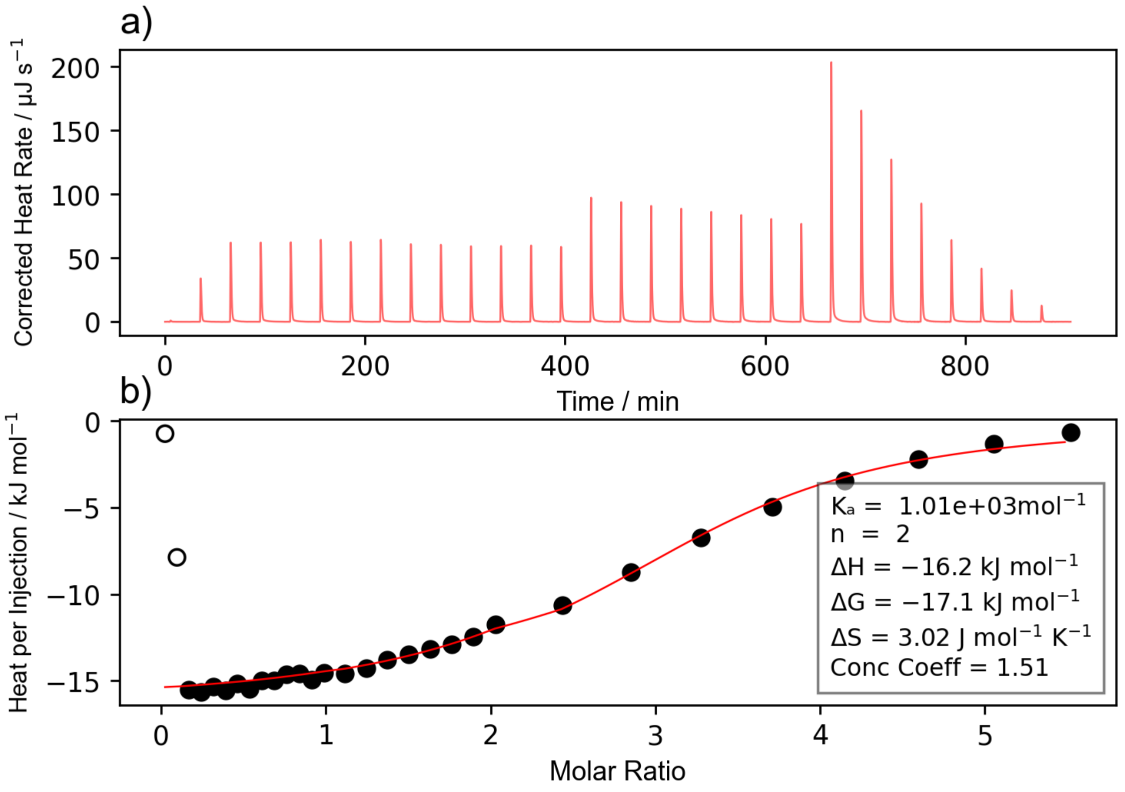

Figure S70 a) Raw thermograph and b) integrated heat data for titration of $[Pd(ttpy)(DMSO)](BF_4)_2$ (109 mM) into **3** (5.7 mM) performed at 298 K. The first and second data point were not included for fitting.

The affinity of **3** and 3-methylpyridine to palladium(II) was directly compared with an NMR competition experiment (Figure S71). The addition of 3-methylpyridine (1.0 equivalent, 15 µmol, 30 mM) to a sample of **3** (1 equivalent, 15 µmol, 30 mM) and $[Pd(ttpy)(DMSO)](BF_4)_2$ (0.36 equivalent, 5.6 µmol, 11 mM) results in the signals for **3** shifting upfield and becoming more resolved, signifying a larger proportion of unbound **3**. This demonstrates that **3** has a lower affinity to palladium(II) than 3-methylpyridine.

14.2. Competitive binding between ligand 3 and 3-methylpyridine with palladium(II)

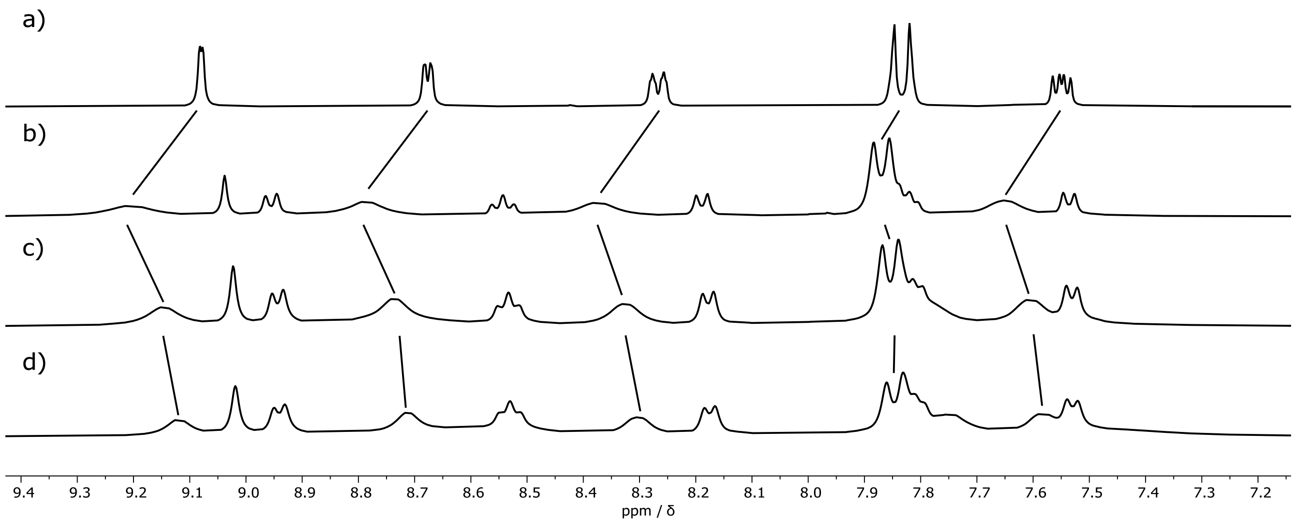

Figure S71 ^{1}H NMR (400 MHz, DMSO-d_6, 298 K) of a) **3** b) **3** (1.0 equiv, 14 µmol, 25 mM) and [Pd(ttpy)(DMSO)]$(BF_4)_2$ (0.40 equiv, 5.6 µmol, 9.8 mM) c) **3** (1.0 equivalent), [Pd(ttpy)(DMSO)]$(BF_4)_2$ (0.40 equiv, 5.6 µmol, 9.8 mM), and 3-methylpyridine (0.60 equiv, 15 µmol, 1.3 mM) d) **3** (1.0 equiv, 14 µmol, 25 mM), [Pd(ttpy)(DMSO)]$(BF_4)_2$ (0.40 equiv, 5.6 µmol, 9.8 mM), and 3-methylpyridine (1.2 equiv, 30 µmol, 25 mM).

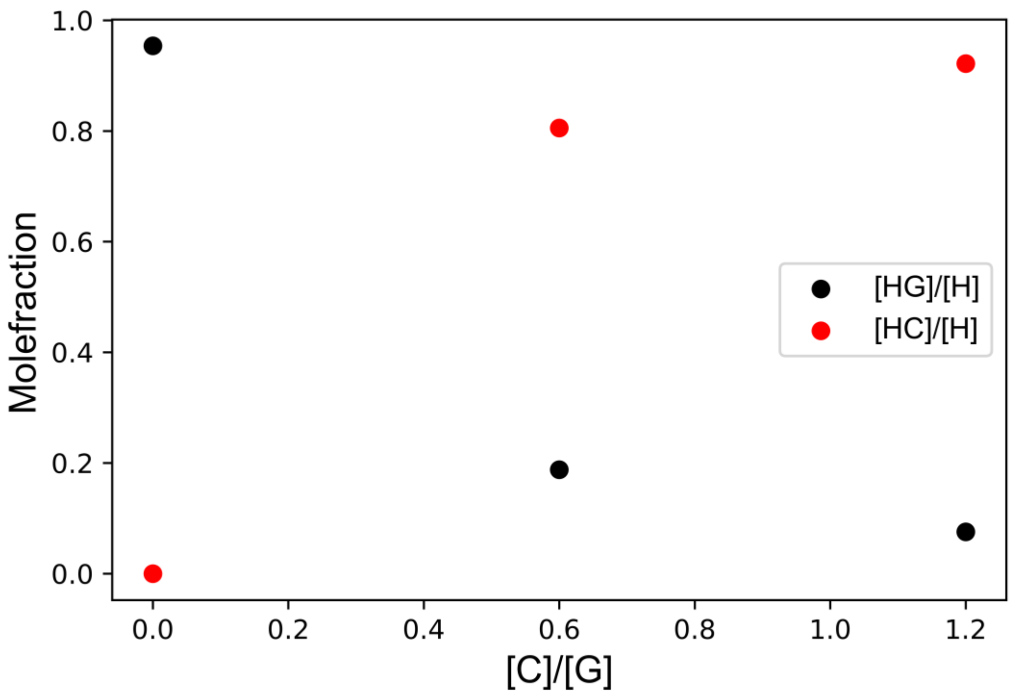

Figure S72 Proportion of guest (G = ligand **3**) and competitor (C = 3-methylpyridine) bound to host (H = [Pd(ttpy)(DMSO)]$(BF_4)_2$) after each addition in Figure S66.

15. Photoswitching of self-assembled structures

15.1. Monitoring of a mixture of $[Pd_3(3)_6]^{6+}$ and $[Pd_4(3)_8]^{8+}$ using UV-vis spectroscopy

For the below UV-vis and NMR experiments the same solution was used to remove any concentration effects on the population of species.

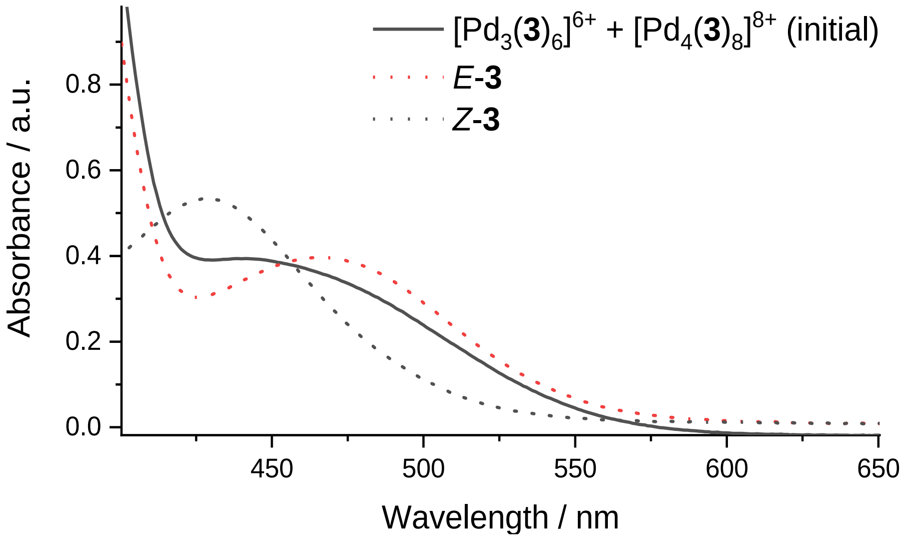

Figure S73 Comparison of the UV-spectra of a mixture of $[Pd_3(\mathbf{3})_6]^{6+}$ and $[Pd_4(\mathbf{3})_8]^{8+}$ in DMSO-d_6 with that of *E*-**3** and *Z*-**3** (calculated).

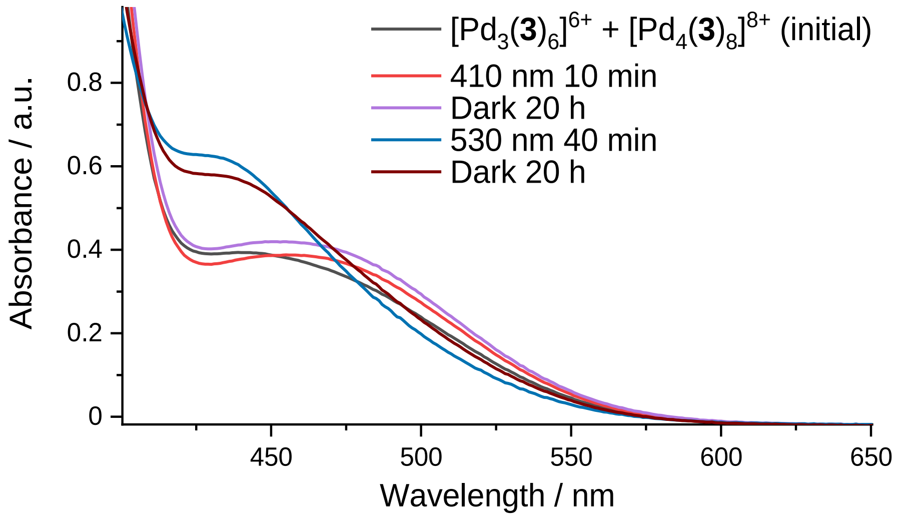

Figure S74 Photoswitching of a mixture of $[Pd_3(\mathbf{3})_6]^{6+}$ and $[Pd_4(\mathbf{3})_8]^{8+}$ in DMSO-d_6 monitored by UV-vis spectroscopy.

15.2. Monitoring of a mixture of $[Pd_3(3)_6]^{6+}$ and $[Pd_4(3)_8]^{8+}$ using NMR spectroscopy

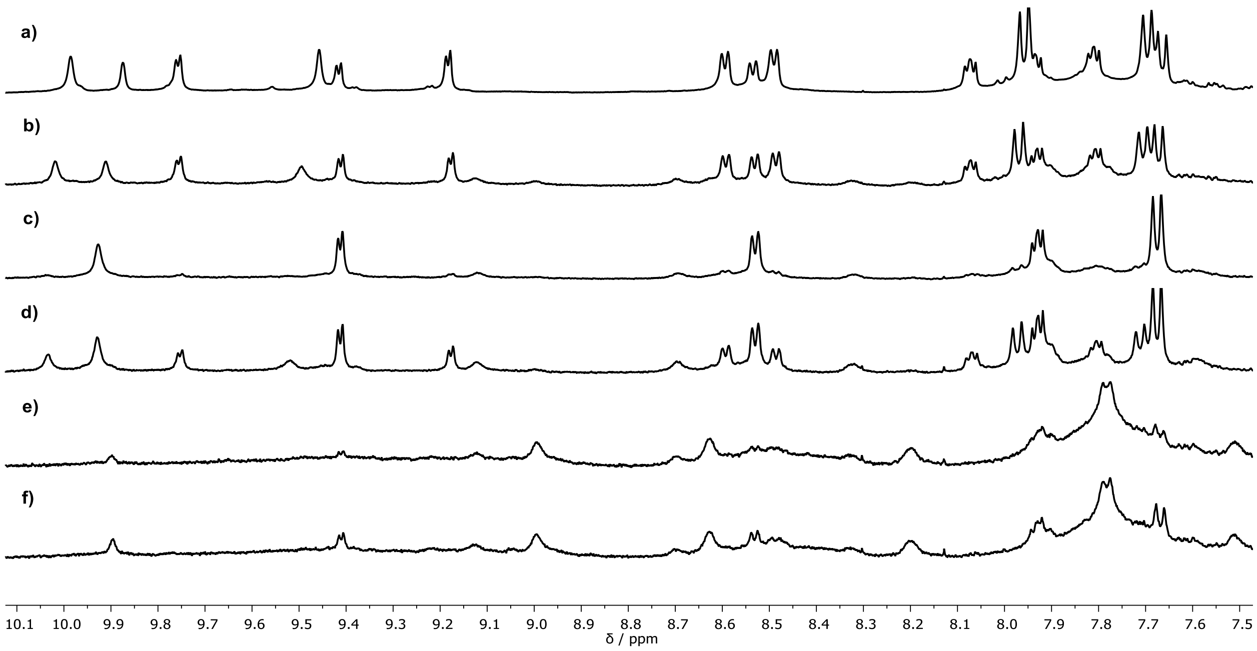

Figure S75 Photoswitching of a mixture of $[Pd_3(\mathbf{3})_6]^{6+}$ and $[Pd_4(\mathbf{3})_8]^{8+}$ in DMSO-d_6. a) Initial mixture of $[Pd_3(\mathbf{3})_6]^{6+}$ and $[Pd_4(\mathbf{3})_8]^{8+}$ (25:75 Pd_3L_6:Pd_4L_8); b) 10× dilution in DMSO-d_6 (32:68 Pd_3L_6:Pd_4L_8); c) Irradiation with 410 nm LED for 10 minutes (≈ 90% Pd_3L_6); d) After 19 h in dark; e) after irradiation with a 530 nm LED (50 minutes) and f) left in dark for 1 h. The thermal isomerisation of the ligand (half-life ~25 days at room temperature) is much slower than the rearrangement of the cages (19h). This is due to the cages being re-formed from the remaining ligands in the *E*-isomer form. i.e. the *Z*-isomer is not thermally switching back any faster than in the absence of palladium(II).

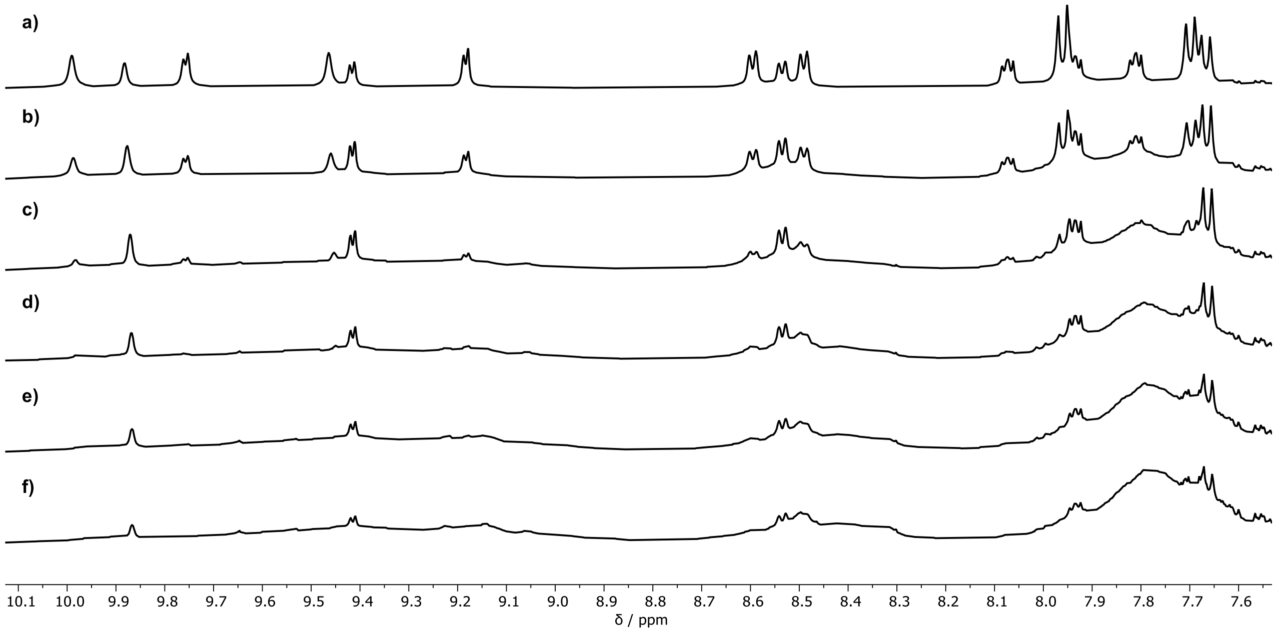

Figure S76 Irradiation of a mixture of $[Pd_3(\mathbf{3})_6]^{6+}$ and $[Pd_4(\mathbf{3})_8]^{8+}$ in DMSO-d_6 with 530 nm light. a) Initial mixture of $[Pd_3(\mathbf{3})_6]^{6+}$ and $[Pd_4(\mathbf{3})_8]^{8+}$ (25:75 Pd_3L_6:Pd_4L_8); b) Irradiation with a 530 nm LED for 10 minutes; c) 20 minutes; d) 30 minutes; e) 40 minutes and f) after 50 minutes irradiation.

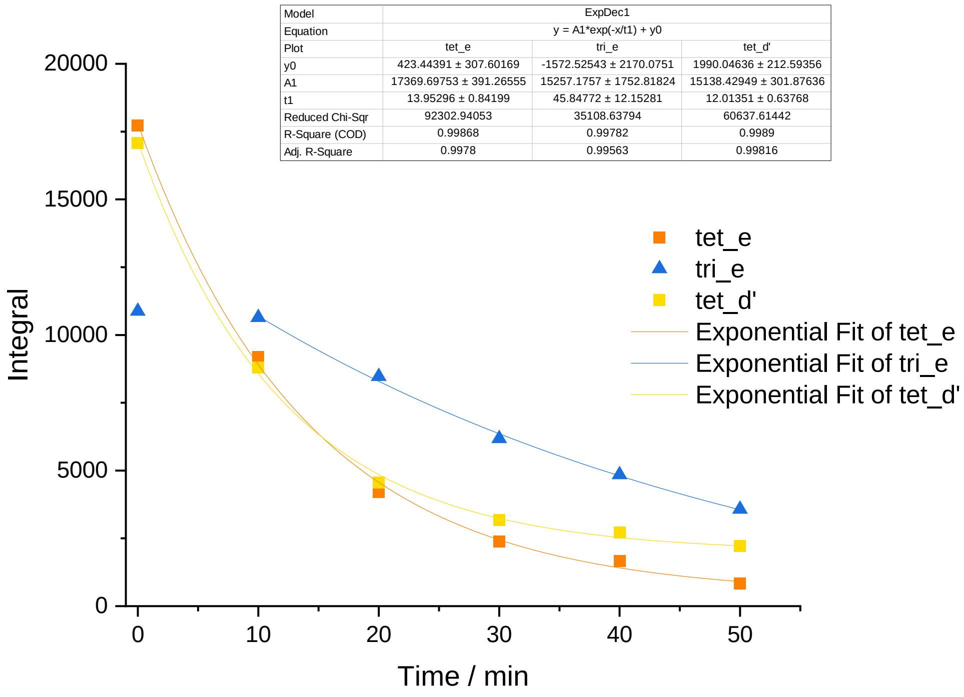

Model	ExpDec1		
Equation	y = A1*exp(-x/t1) + y0		
Plot	tet_e	tri_e	tet_d'
y0	423.44391 ± 307.60169	-1572.52543 ± 2170.0751	1990.04636 ± 212.59356
A1	17369.69753 ± 391.26555	15257.1757 ± 1752.81824	15138.42949 ± 301.87636
t1	13.95296 ± 0.84199	45.84772 ± 12.15281	12.01351 ± 0.63768
Reduced Chi-Sqr	92302.94053	35108.63794	60637.61442
R-Square (COD)	0.99868	0.99782	0.9989
Adj. R-Square	0.9978	0.99563	0.99816

Figure S77 Monitoring the consumption of the $[Pd_3(\mathbf{3})_6]^{6+}$ and $[Pd_4(\mathbf{3})_8]^{8+}$ species during irradiation with a 530 nm LED. Due to signal overlap only the most downfield peaks have been fit, using a mono-exponential model.

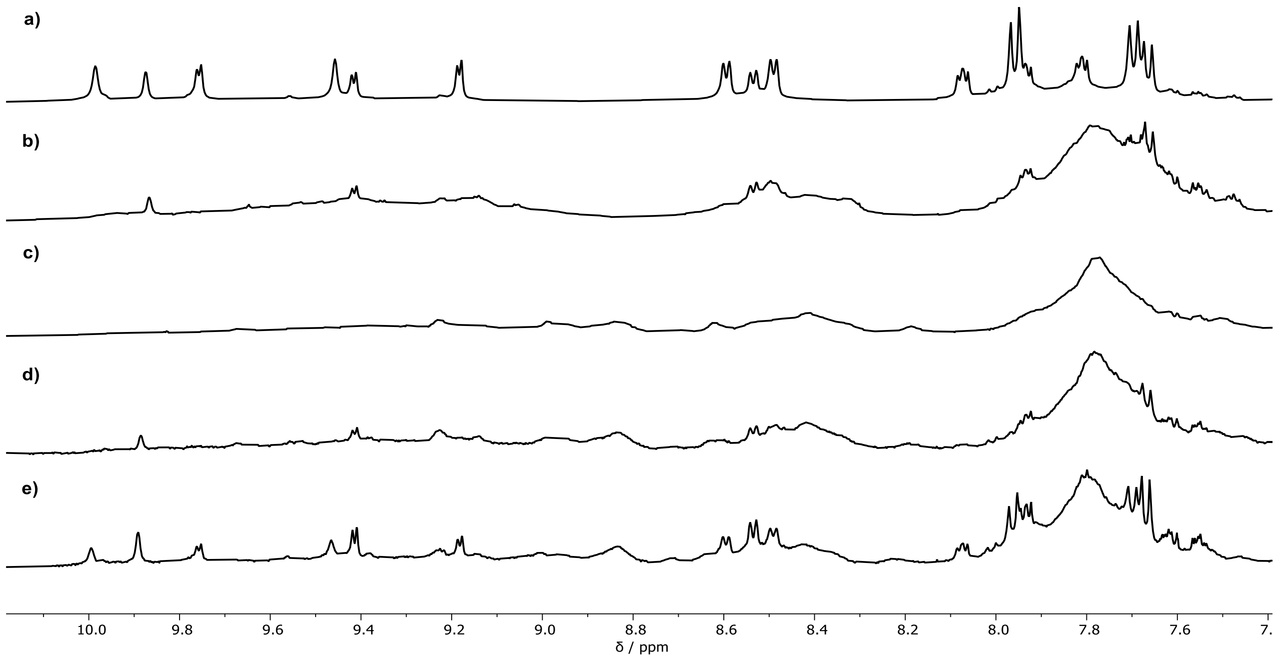

Figure S78 ^{1}H NMR comparison of a) an equilibrated mixture of $[Pd_3(\mathbf{3})_6]^{6+}$ and $[Pd_4(\mathbf{3})_8]^{8+}$ formed from *E*-**3**; b) after irradiation at 530 nm for 50 minutes; c) initial combination of enriched *Z*-**3** and $[Pd(CH_3CN)_4](BF_4)_2$ and equilibration of the previous sample in the dark d) 75 minutes and e) 5 days.

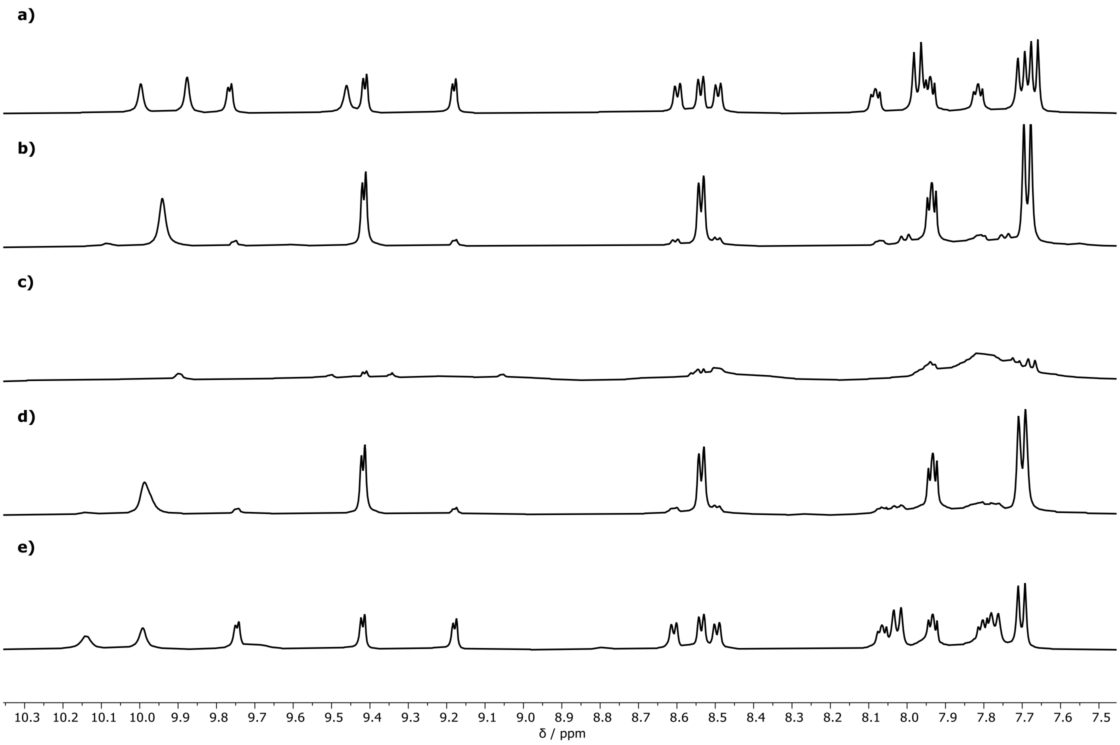

Figure S79 ^{1}H NMR spectra (DMSO-d_6, 600 MHz, 298 K) of a) a mixture $[Pd_3(\mathbf{3})_6]^{6+}$ and $[Pd_4(\mathbf{3})_8]^{8+}$ in DMSO-d_6 (44% $[Pd_3(\mathbf{3})_6]^{6+}$); b) the same sample after irradiation with 410 nm light for 10 minutes (82% $[Pd_3(\mathbf{3})_6]^{6+}$); c) the same sample after irradiation with 530 nm light for 10 minutes; d) the same sample after irradiation with 410 nm light for 10 minutes again; and e) the same sample after 2 days of being heated at 60 °C followed by 6 h of equilibrating at room temperature.

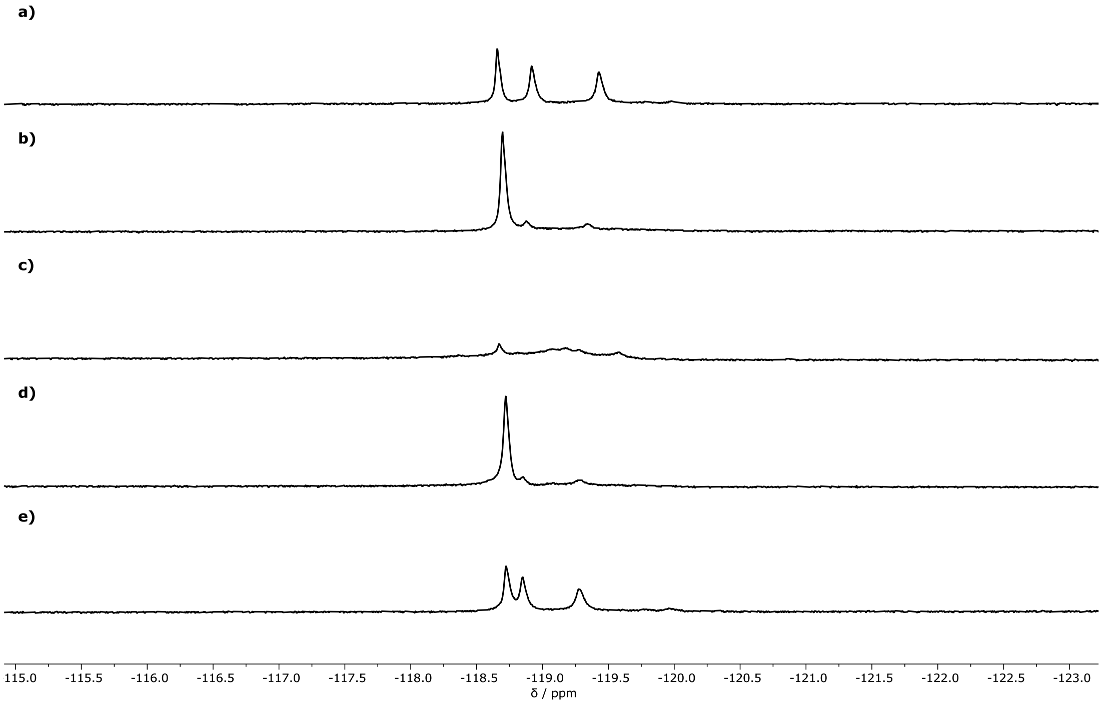

Figure S80 ^{19}F NMR spectra (DMSO-d_6, 565 MHz, 298 K) of a) a mixture $[Pd_3(\mathbf{3})_6]^{6+}$ and $[Pd_4(\mathbf{3})_8]^{8+}$ in DMSO-d_6 (44% $[Pd_3(\mathbf{3})_6]^{6+}$); b) the same sample after irradiation with 410 nm light for 10 minutes (82% $[Pd_3(\mathbf{3})_6]^{6+}$); c) the same sample after irradiation with 530 nm light for 10 minutes; d) the same sample after irradiation with 410 nm light for 10 minutes again; and e) the same sample after 2 days of being heated at 60 °C followed by 6 h of equilibrating at room temperature.

15.3. Mass spectrometry data during photoswitching

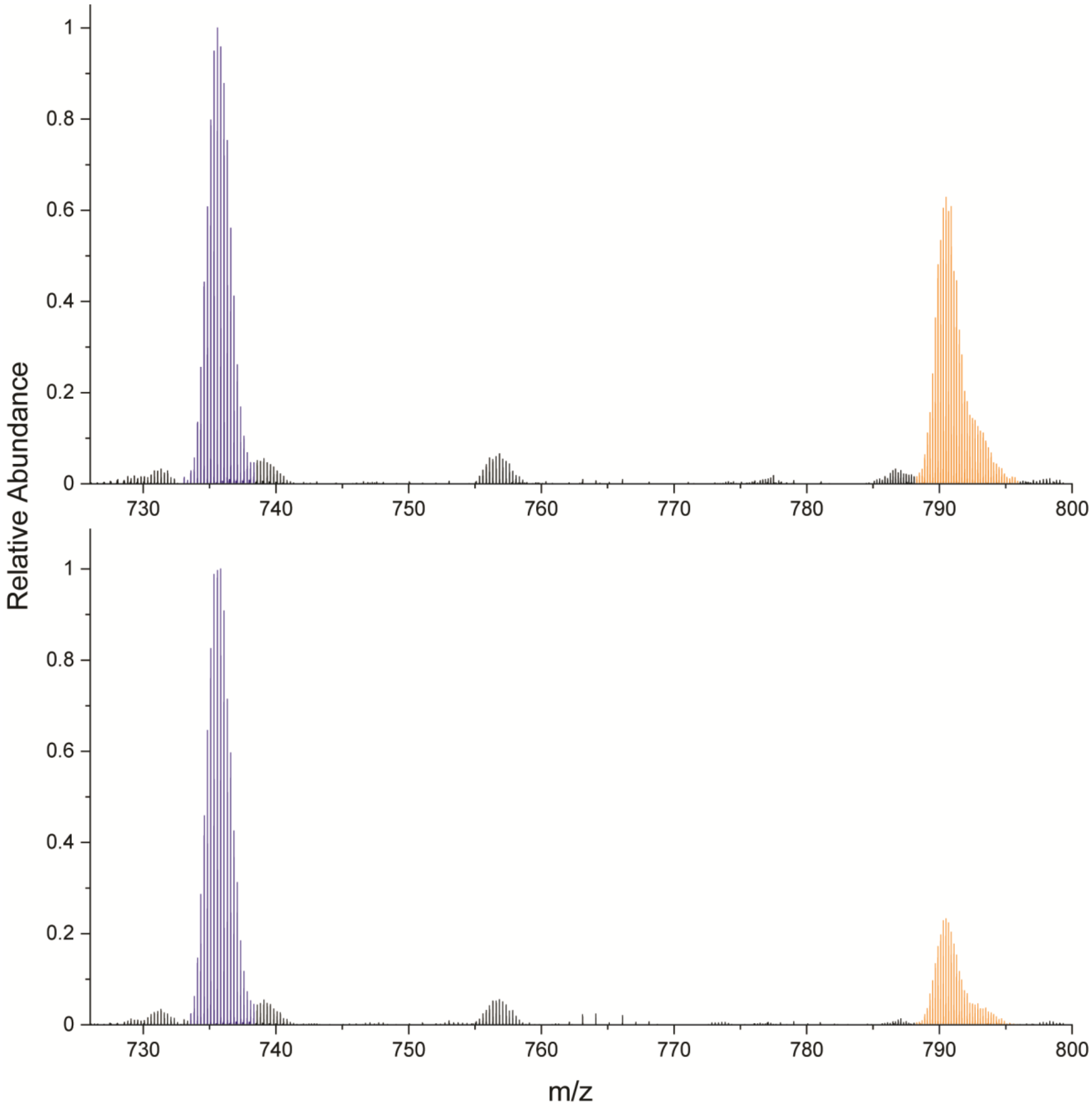

Figure S81 Perturbing the equilibrium of $[Pd_3(\mathbf{3})_6]^{6+}$ (blue) and $[Pd_4(\mathbf{3})_8]^{8+}$ (orange) in DMSO-d_6 monitored by HR ESI-MS. a) Isotope patterns of a mixture of $[Pd_3(\mathbf{3})_6 + 2(BF_4)]^{4+}$ and $[Pd_4(\mathbf{3})_8 + 3(BF_4)]^{5+}$; b) after 5 minutes irradiation with a 410 nm LED.

16. References

[1] C. Knie, M. Utecht, F. Zhao, H. Kulla, S. Kovalenko, A. M. Brouwer, P. Saalfrank, S. Hecht, D. Bléger, *Chem.– Eur. J.* **2014**, *20*, 16492-16501.
[2] D. Bléger, J. Schwarz, A. M. Brouwer, S. Hecht, *J. Am. Chem. Soc.* **2012**, *134*, 20597-20600.
[3] S. Iamsaard, E. Anger, S. J. Aßhoff, A. Depauw, S. P. Fletcher, N. Katsonis, *Angew. Chem. Int. Ed.* **2016**, *55*, 9908-9912.
[4] G. Sheldrick, University of Göttingen, Germany, **1996**.
[5] Apex3, Bruker AXS Inc, Madison, WI, USA, **2016**.
[6] G. Sheldrick, *Acta Crystallograph. Sect. A* **2015**, *71*, 3-8.
[7] O. V. Dolomanov, L. J. Bourhis, R. J. Gildea, J. A. K. Howard, H. Puschmann, *J. Appl. Crystallogr.* **2009**, *42*, 339-341.
[8] S. Kai, Y. Sakuma, T. Mashiko, T. Kojima, M. Tachikawa, S. Hiraoka, *Inorg. Chem.* **2017**, *56*, 12652-12663.
[9] T. Tateishi, S. Takahashi, A. Okazawa, V. Martí-Centelles, J. Wang, T. Kojima, P. J. Lusby, H. Sato, S. Hiraoka, *J. Am. Chem. Soc.* **2019**, *141*, 19669-19676.
[10] M. W. Cooke, J. Wang, T. Isabelle, G. S. Hannan, *Synth. Commun.* **2006**, *36*, 1721-1726.
[11] J. Schleucher, J. Quant, S. J. Glaser, C. Griesinger, in *Encyclopedia of Magnetic Resonance* (Ed.: R. L. W. R.K. Harris), **2007**.
